# Yap1 regulates motility and vertebral development and prevents kyphoscoliosis in zebrafish

**DOI:** 10.1371/journal.pgen.1012172

**Published:** 2026-05-28

**Authors:** Victoria C. Williams-Ward, Kees Wanders, Simon M. Hughes

**Affiliations:** Randall Centre for Cell and Molecular Biophysics, New Hunt’s House, Guy’s Campus, King’s College London, London, United Kingdom; Fred Hutchinson Cancer Research Center, UNITED STATES OF AMERICA

## Abstract

Scoliosis affects 2–3% of people, often developing during and after adolescence, and currently has a lifetime chance of surgical intervention of ~0.1% in high income countries. Understanding of causal genetic and environmental factors is improving, with mechanical feedback interactions between the neuromuscular and skeletal systems thought to be important. While examining mechanosignalling in the zebrafish musculoskeletal system, we observed transient expression of *yap1* mRNA in precursor cells of muscle and notochord and *wwtr1* mRNA accumulation in differentiated muscle. Yap1 and Wwtr1/Taz are transcriptional coactivators that mediate Hippo pathway signalling, often in response to mechanosignals. Loss of function mutation of either gene alone transiently altered early larval motility and reduced survival to adulthood, but mutation of *yap1* specifically diminished overall growth without an obvious histological muscle defect. *Yap1* mutants had a temperature-sensitive phenotype of oedema in cardiac and other tissues, which could be rescued by rearing at low temperature. Rescued *yap1* mutants showed focal defects in hypochordal *col8a1a* mRNA expression at 1–2 days post-fertilisation (dpf), an early motility defect at 5 dpf and subsequently developed a fully penetrant vertebral dysmorphology, reflected by a decrease in posterior vertebral height. Thereafter, frank kyphoscoliosis accompanied by additional vertebral defects developed in around a third of the surviving *yap1* mutants and was first detected at 11 dpf. Thus, the mild initial vertebral defect can, in a predisposing genetic or environmental background, gradually develop into full kyphoscoliosis through a positive feedback mechanism, analogous to the Hueter-Volkmann ‘Law’. Although the cell type/s of cell autonomous *yap1* action remain unclear, we hypothesise that Yap1 mechanosensation mediates feedback between bone, muscle and tendon to restrain vertebral overgrowth and protect against the development of kyphoscoliosis.

## Author summary

Scoliosis occurs in 2–3% of people, often arising during adolescence and more commonly in females. Scoliosis occasions surgical intervention in about 1 person in a thousand and is thought to develop through an imbalance of forces exerted on the skeleton by muscle contraction. However, initiating primary genetic and environmental cause(s) of scoliosis are largely unclear. During analysis of zebrafish lacking the Yap1 force sensor protein, we noticed the gradual development of scoliosis in about a third of *yap1* mutant larvae. Larvae express the *yap1* gene during development in both bone and muscle precursor cells. Early defects observed in mutants include altered *col8a1a* mRNA in the hypochord signalling centre, followed by reduced muscle function and swimming ability and growth rate. Fish lacking Wwtr1/Taz, a Yap1-like protein normally expressed in newly-formed muscle but not detected in bone precursor cells, show a similar early motility defect, but recover, grow normally and lack scoliosis. These findings suggest that correct Yap1-dependent force-sensing may be required during rapid musculoskeletal growth phases, together with other genetic and/or environmental factors, to ensure balanced and symmetrical spine development.

## Introduction

Familial genetics and genome wide association studies (GWAS) have implicated a number of genes involved in neuromusculoskeletal development and matrix biology in the aetiology of adolescent idiopathic scoliosis (AIS), which affects 2–3% of children and is more common in females [1–3]. However, the identified genes only account for a small fraction of AIS heritability, indicating heterogeneity of aetiology and pathogenesis [4]. Expanding genetic understanding of scoliosis with a view to illuminating environmental interactors that could mitigate the typically teenage onset is thus a priority.

Muscle-specific diseases such as Duchenne Muscular Dystrophy frequently lead to scoliosis [5]. Moreover, a recent murine study found that defective muscle development causes scoliosis and implicated the Yap1 mechanosensing system in its aetiology [6]. Within the musculoskeletal system, muscles generate force that is transmitted by tendons to cartilage and bone. During development, intercellular signals that pass between these tissues are required for the assembly of attachments [7–10]. Signals from muscle are required for skeletal development [11,12] and muscle contraction itself is required for normal muscle, tendon, cartilage, and bone growth [13–23]. Reciprocally, reduced force signals in osteoblasts lead to impaired muscle formation [24]. Even in the adult, high-force exercise is known to be a potent hypertrophic stimulus [17,23,25–27]. Conversely, osteoporosis and ageing-related muscle wasting (sarcopenia) are exacerbated by inactivity [28–33]. Moreover, clinical observations suggest that altered force can trigger pathological positive feedback that exacerbates kyphoscoliosis and other skeletal pathologies through inappropriate skeletal remodelling [34–39]. Given the heavy burden of musculoskeletal problems, particularly in the elderly, and the relative ease of modulating force through exercise, it is important to understand how force regulates the musculoskeletal system.

The Hippo pathway, which often mediates force-derived signals [40,41], is implicated in both muscular and skeletal response to force [42–44]. The canonical Hippo pathway integrates a variety of signals, including mechanical cues, through a protein kinase signalling cascade that focuses onto two proteins, Yap1 and Wwtr1/Taz [45]. Yap1 and Wwtr1 are transcriptional coactivators which, when activated by dephosphorylation, enter the cell nucleus and interact with a range of transcription factors to regulate (usually up-regulate) expression of their target genes [45,46]. Although Yap1 and Wwtr1 are widely expressed, the presence of distinct combinations of interacting transcription factors in each cell type allows exquisite tissue-specific tuning of the cellular response to mechanosignalling mediated by Yap1 and Wwtr1 [47–49]. Yap1/Wwtr1 activity can be regulated by cytoskeletal tension [50,51], ligand density [52] and ECM composition [53].

Both Yap1 and Wwtr1 have been implicated in musculoskeletal development, where it is suggested that they could mediate mechanosignals [54–60]. TEAD transcription factors, which strongly interact with Yap1 and Wwtr1, were originally described as the muscle-specific MCAT-binding transcription factor [61,62] (reviewed in [63]). In Drosophila, the single Yap1/Wwtr1 homologue, Yorkie, mediates Hippo signalling that restricts muscle size [64,65]. In vertebrates, murine *Yap1* knockouts are embryonic lethal at E8.5, prior to musculoskeletal development [66]. In vitro, *Yap1* knockdown reduces murine muscle stem cell proliferation, whereas Yap1 overexpression promotes proliferation and inhibits terminal differentiation [67]. In vivo, Yap1 activation in muscle fibres either triggers atrophic myopathy [68] or hypertrophy [69]. Yap1 and Wwtr1 within non-muscle cells in muscle tissue may also regulate muscle growth [70]. The 20% of *Wwtr1* knockout mice that survive to weaning [55,71,72] have low body weight and smaller muscles [58]. Muscle-specific deletion of *Yap1* or *Wwtr1* leads to weakness with mild neuromuscular junction defects that are more marked in adult *Wwtr1* mutants [73]. Muscle-specific deletion of both genes is lethal at birth due to severe muscle defects with sarcomere disorganisation [73]. Thus, Wwtr1 and Yap1 functions are required for normal muscle development.

Wwtr1 has also been implicated in bone formation based on knockdown experiments in cultured cells and zebrafish and a molecular interaction with the osteogenic transcription factor Runx2 [74]. However, no obvious defect in bone formation was reported in initial murine *Wwtr1* knockouts [55,71,72]. The results of modulating Yap1 and Wwtr1 activities in developing bones in mice have been reported and yield a complex picture. Both gain and loss of function experiments have been performed specifically in chondrocytes, chiefly using a *Collagen2a1* driver [59,75,76]. Removal of Yap1 from cartilage yielded slightly longer bones with a slight increase in mineralization [75]. The converse experiment of over-expressing Yap1 in cartilage using the same *Col2a1* regulatory elements caused a dose-dependent shortening of long bones and vertebrae and reduction of growth plate size [75]. Similarly, dramatic shortening of bones and chondrodysplasia was observed when over-expressing constitutively nuclear (i.e., potentially active) Yap1 or by activating endogenous Yap1-family proteins through ablation of their upstream inhibitors in the Hippo pathway, Lats1 and Lats2 [59]. Another study reported Yap1 as essential for osteoclastogenesis through a TEAD-dependent mechanism [77]. In contrast, Li et al. [76] deleted *Wwtr1*, also using *Col2a1:Cre*, and observed a converse phenotype, with decreased long bone length, reduced growth plate length and less expression of chondrocyte marker genes. Vanyai et al. [59] ablated both Yap1 and Wwtr1 in chondrocytes, again using *Col2a1:Cre*, and detected various subtle bone malformations and an increase in long bone length with an enlarged growth plate but little change in overall bone formation at E17.5. Nevertheless, when chondrocyte proliferation was examined, little change was observed in the dual knockout [59], whereas the *Yap1* and *Wwtr1* single knockouts were each described as having reduced chondrocyte proliferation [75,76]. In another study, Wwtr1 was reported to regulate bone formation through a mechanosensing mechanism [78]. While clearly showing a role for Yap1 and Wwtr1 in cartilage/bone formation, these studies leave the precise role of each gene at various stages of skeletal differentiation unclear.

Here we report genetic analysis of the role/s of Yap1 and Wwtr1 in the zebrafish musculoskeletal system. We initially focus on early developmental stages, when myotomal muscle and its associated vertebral skeleton arise by well-characterised processes. In muscle, transient early *yap1* expression is followed by later *wwtr1* expression as muscle precursors undergo terminal differentiation into fibres. Initial myogenesis and notochord formation appeared normal in *yap1*, *wwtr1*, or double loss of function mutants, but hypochord and motility defects soon became apparent in *yap1* mutants. Defects in musculoskeletal growth were observed in *yap1* mutants, which grew more slowly than their non-mutant siblings. About a third of *yap1* mutant individuals subsequently developed kyphoscoliosis. Although double mutant muscle could not be analysed due to early lethality [79], redundant function between the two proteins is suggested as heterozygosity of *yap1* in *wwtr1* mutants prevents larval survival. We conclude that Yap1 and Wwtr1 are required for full functional muscle in early larval growth, and that Yap1 plays a role in vertebral column development in interaction with other genetic or environmental determinants of kyphoscoliosis.

## Results

### Sequential *yap1* and *wwtr1* expression in the musculoskeletal system

**Fig 1:**
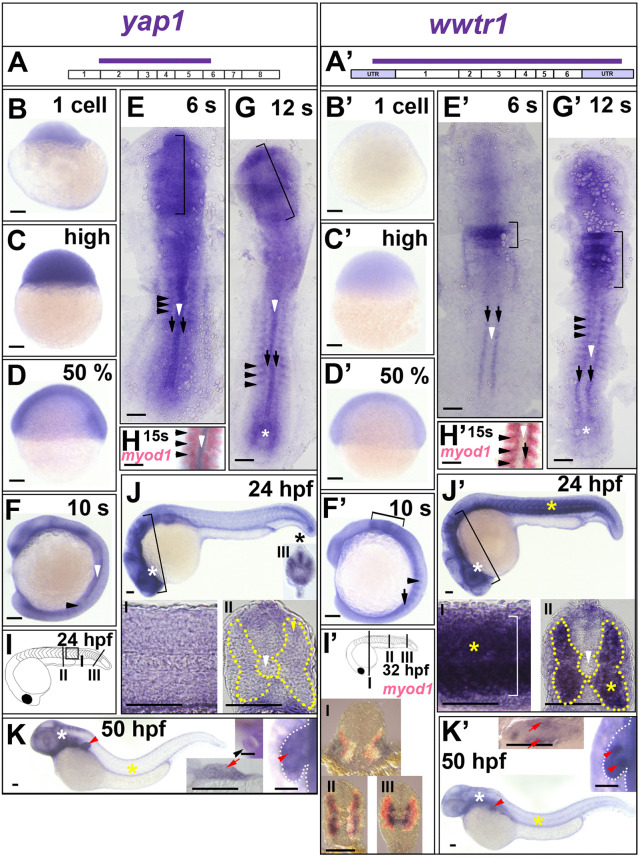
Expression of *yap1* and *wwtr1* mRNAs during early development. **(A,A’)** Schematic indicating the location of antisense probe on each mRNA transcript. In situ mRNA hybridisation for *yap1* mRNA (B-K) or *wwtr1* mRNA (B’-K’) shown in lateral whole mount, anterior to top (B,B’-D,D’, F,F’, J,J’, K,K’), dorsal flat mount, anterior to top (E,E’, G,G’, H,H’) or transverse cryo-section (B,B’-D,D’, J, J’, K, K’). Maternal *yap1* mRNA augmented by zygotic expression at high stage, contrasted with uniform *wwtr1* mRNA zygotic expression (C,C’). **(E)** At 6ss**,** *yap1* mRNA is widespread in forebrain (bracket), notochord (white arrowhead) and lateral somite stripes (black arrowheads), but less apparent in adaxial slow muscle precursors (black arrows). **(E’)** *Wwtr1* mRNA appears more restricted to mid/hindbrain (bracket) and adaxial cells (black arrows), but is absent from notochord (white arrowhead). **(F,G)** At 10-12ss, notochord (white arrowhead), forebrain (bracket), tail bud (white asterisk) and lateral stripes in nascent somites (black arrowheads) contain *yap1* mRNA, but expression appears diminished in more mature somites. **(F’,G’)** Accumulation of *wwtr1* mRNA is observed in adaxial cells (black arrows), stripes in lateral somites (black arrowheads) and mid/hindbrain (bracket), but not in notochord (white arrowhead) or tail bud (white asterisk). **(H)** At 15ss, *myod1* mRNA with *yap1* mRNA localises in the anterior half of the somites, alternating with *myod1* mRNA (black arrowheads) and notochord (white arrowhead). **(H’**) Conversely, *wwtr1* mRNA co-localises with the *myod1* mRNA in the posterior half of each somite (black arrowheads) and adaxial cells (black arrow). **(I)** Schematic indicating location of flat-mount (I) and 20 μm transverse cryosections (II and III) in J and J’. **I’**. Schematic indicating location of 15 μm transverse cryosections showing *wwtr1* and *myod1* mRNA in somitic regions at 32 hpf. **(J, J’)** At 24 hpf, low level *yap1* mRNA was present in dorsal tip (yellow arrowhead) of somite (outlined in yellow dots), whereas *wwtr1* mRNA was detected throughout the somitic myotome (yellow asterisks). Both *yap1* and *wwtr1* mRNAs were present in the eye (white asterisks) and head (brackets). *Yap1* mRNA persisted in the tail bud (black asterisk). **(K, K’)** At 50 hpf, *yap1* mRNA persisted at dorsal and ventral somite extremes and was present in heart (inset, black arrowhead) and pectoral fin (red arrowheads) whether viewed from anterior or in 20 μm transverse cryosection (red arrow). *Yap1* and *wwtr1* mRNAs persisted in the head (white asterisks), but both were reduced in the myotome (yellow asterisks). In pectoral fin, *wwtr1* mRNA was detected in two regions consistent with the differentiating muscle masses (red arrows and arrowheads)**.** Bars = 100 μm, except K and K’ 50 μm.

*Yap1* mRNA is expressed widely in early zebrafish development (Fig 1A–1K) and clearly as slow and fast myogenesis are initiated, respectively, in presomitic pre-adaxial cells and mediolateral stripes of the anterior border cells within each forming somite (Fig 1E and 1G; [80]). When adaxial and posterior somite cells become postmitotic en route to myogenesis, *yap1* mRNA is downregulated (Fig 1E–1H), both as adaxial cells undergo terminal differentiation and as presomitic cells become somitic and upregulate *myod1* mRNA (Fig 1E,1G, and 1H). By 24 hpf, *yap1* mRNA has diminished in terminally-differentiated slow and fast muscle fibres, but remains present in the dermomyotomal cells located on the lateral surface of the myotome, which include precursor cells for later muscle growth (Fig 1J). As muscle precursors undergo terminal differentiation into fibres, *yap1* is replaced by *wwtr1* mRNA (Fig 1E’,1G’, and 1J’) in cells that have down-regulated *myod1* mRNA (Fig 1H’ and 1I’). A similar pattern of *yap1* mRNA in superficial precursors and *wwtr1* mRNA in differentiated fibres is observed during pectoral fin myogenesis (Fig 1K and 1K’). Thus, *yap1* and *wwtr1* show sequential expression during early myogenesis.

*Yap1* mRNA also accumulates in the nascent notochord before being down-regulated throughout its length by 24 hpf (Fig 1E,1F,1G and 1J). *Wwtr1* mRNA, however, was not detected in the notochord or in nascent fin cartilage (Fig 1E’,1F’,1G’ and 1K’).

### Myod is required to suppress *yap1* and induce *wwtr1* mRNA in somites

Skeletal myogenesis in zebrafish is driven by the myogenic regulatory factors (MRFs) Myf5, Myod, Myogenin and Mrf4/Myf6 [12,81–83]. Whereas *yap1* mRNA precedes expression of the myogenic transcription factor *myod1*, *wwtr1* mRNA is expressed after *myod1* mRNA in differentiated muscle deep within the myotome (Fig 1H,1I and 1H’,1I’). The time course of *wwtr1* mRNA accumulation matches that of *myog* and *myf6* [83]. To test the relationship between myogenesis and *yap1*/*wwtr1* expression, embryos lacking each MRF gene were subjected to in situ mRNA hybridisation for *yap1* and *wwtr1* and compared to their non-mutant siblings. Loss of *myod1* function delays differentiation of fast muscle [12,84]. Congruently, *myod1* mutants show reduced *wwtr1* expression and persistence of *yap1* mRNA in the lateral somite (S1A and S1B Fig). As expected, expression of *yap1* and *wwtr1* was unaltered in other regions of *myod1* mutant embryos (S1A and S1B Fig). Mutation of *myog* does not prevent muscle terminal differentiation, but reduces fusion into multinucleate fibres [81]. *Yap1* mRNA appeared unaltered in *myog* mutants, whereas *wwtr1* mRNA was somewhat reduced in muscle tissue (S1C and S1D Fig). Loss of function of either *myf5* or *myf6* had no discernible effect on *yap1* or *wwtr1* mRNA accumulation (S1E-S1H Fig). These observations support the view that a switch from expression of Yap1 to Wwtr1 accompanies zebrafish skeletal muscle terminal differentiation.

### Mildly impaired larval movement in *wwtr1* mutant

**Fig 2:**
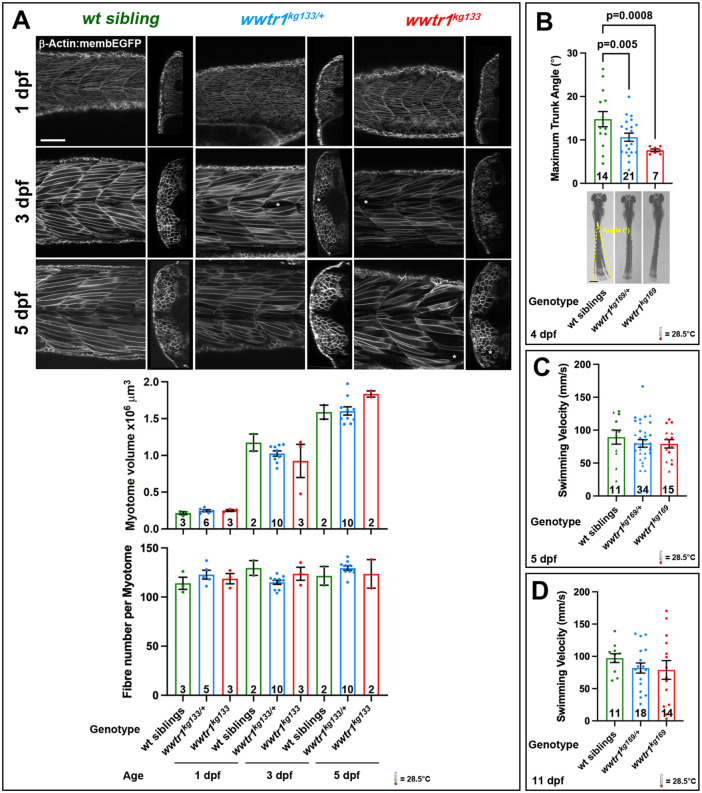
*Wwtr1* mutants are defective in evoked motility. **(A)** Muscle growth analysed in sibling wild type (wt), heterozygote and *wwtr1*^*kg133*^ mutant bred on the *ß-Actin:membEGFP* background and reared at 28.5ºC until 5 dpf and then at 26.5ºC to the indicated age. *Wwtr1*^*kg133*^ mutants and their siblings have similar muscle growth. Note that apparent gaps (asterisks) between fibres reflect reporter mosaicism, not absence of fibres. Bar = 50 μm. **(B)** Fish movement in response to electrical stimulation under anaesthetic was reduced in *wwtr1*^*kg169*^ mutants compared to siblings. Bar = 500 μm. **(C,D).** Swim velocity in response to touch stimulation at 5 dpf (C) and 11 dpf **(D)**. Number of fish analysed is indicated on columns. Significant statistical results are shown (A-D) for Kruskal-Wallis tests with Dunn’s post hoc comparisons, with the exception of the highly significant main effect of age is in **A.**

To test the role of Yap1 and Wwtr1 in the musculoskeletal system, each gene was mutated using TALEN-mediated genome editing in the coding region of the first coding exon (S2A and S2B Fig). Among various nonsense alleles obtained of each gene, a trend in nonsense-mediated mRNA decay (NMD) was observed depending on nonsense tail length. One class of mutants had long nonsense tails and showed strong NMD (S2C-S2E Fig). Mutants shifting to the other nonsense reading frame resulted in a short nonsense tail and much less NMD (S2F and S2G Fig). *Yap1* mRNA was not detectably upregulated in *wwtr1* mutant larvae (S2H Fig). *Wwtr1* mutants, when crossed onto a membrane-EGFP reporter to permit confocal analysis [85], did not show an obvious morphological defect before 5 dpf and muscle growth appeared to be normal (Figs 2A and S3A). However, fewer *wwtr1*^*kg169/kg169*^ mutants (we use the form *wwtr1*^*kg169*^ for such homozygote mutants hereafter) survived to adulthood than their co-habiting siblings (S3E Fig, n = 165, Χ^2^ *p* = 0.017). To ask if the lack of Wwtr1 affected muscle function, we used two movement assays. At 4 dpf, larvae from a *wwtr1*^*kg169/+*^ in-cross were anaesthetised and tested for their response to electrical stimulation by measuring the maximum angle of evoked trunk movement. *Wwtr1*^*kg169*^ mutants moved less than their siblings (*p* = 0.02; Fig 2B). However, *wwtr1*^*kg169*^ mutants did not show a slower swim speed response to touch at either 5 or 11 dpf (Fig 2C and 2D) and grew normally at later stages (see below, Fig 4B). Whereas electrically-evoked tail movement likely reflects a high force regime in which movement may be limited by altered trunk structure or extracellular matrix deposition, swimming speed may represent a lower force regime under which a motility defect is not apparent. These results show that Wwtr1 is not essential for muscle growth but is required transiently for optimal muscle function during early larval stages.

**Fig 3:**
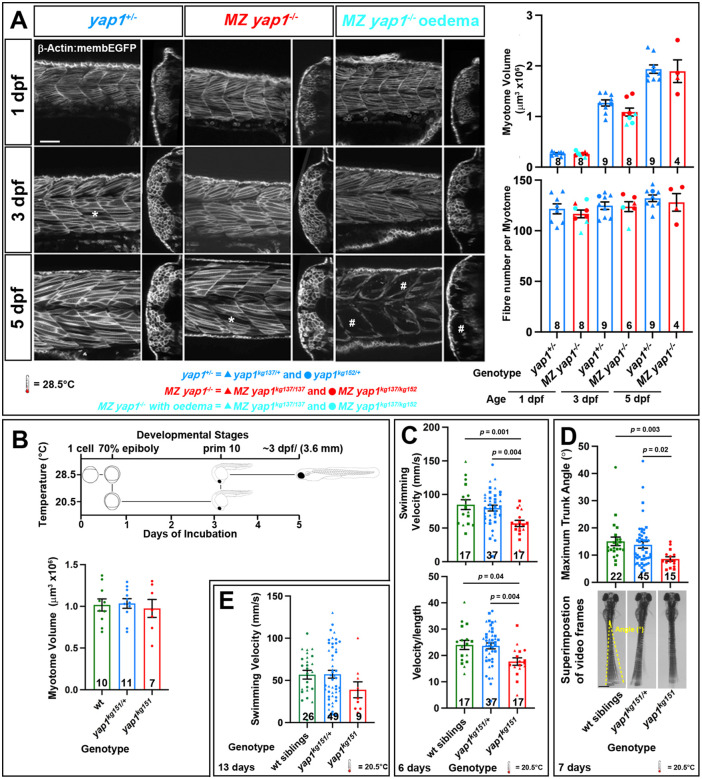
*Yap1*^*kg151*^ mutants are defective in larval movement. Muscle properties analysed in *yap1* mutants and their non-mutant siblings on the *ß-Actin:membEGFP* background reared at the indicated temperature from 70% epiboly until prim 10 (B, see schematic) or 5 dpf-equivalent (A,C-E) and 26.5ºC thereafter (E). **(A)** *MZyap1*^*kg137*^ or *MZyap1*^*kg137*/kg152^ mutants and their heterozygote siblings grown at non-permissive temperature have similar muscle growth. Lack of signal in occasional fibres due to mosaic transgene expression (asterisks) is distinguished from broad loss of fluorescence (#) in oedematous MZ mutants at 5 dpf. Bar = 50 μm. **(B)** *Yap1*^*kg151*^ mutants grown as schematised had myotome volume indistinguishable from their heterozygous and wt siblings**. (C-E)** *Yap1*^*kg151/+*^ in-cross grown at permissive temperature from 70% epiboly**.** Symbol shape indicates lay. Bar = 500 μm. **(C)** *Yap1*^*kg151*^ mutants at 6 days (equivalent to ~4.25 dpf) swim away from touch stimulation slower than their siblings (upper panel), even when corrected for shorter total length (lower panel). **(D)** When stimulated by electrical pulses, *yap1*^*kg151*^ mutants at 7 days (equivalent to 5 dpf) move their trunk through a smaller angle in comparison to their siblings. **(E)** Swimming velocity of *yap1*^*kg151*^ mutants at 13 days (equivalent to 11 dpf) is variable between lays. Symbol shapes represent different lays/experiments (3 lays C, 2 lays D,E). Number of fish analysed is indicated on columns. Significant statistical results are shown (A-E) for Kruskal-Wallis tests with Dunn’s post hoc comparisons.

### *Yap1* mutant fish are defective in movement

We next assessed the phenotype of homozygous *yap1* mutants observing, as previously reported, temperature-dependent variability [86,87]; S3B and S3C Fig). At the normal rearing temperature of 28.5 ºC, some *yap1* mutants had reduced eyes, pericardial oedema and shortened or curled bodies (S3B and S3C Fig). When two lays were reared to adulthood at 28.5 ºC, no mutants were found among 97 surviving siblings (Χ^2^, *p* = 1 x 10^-8^). Reared at a higher, restrictive temperature of 32 ºC, eye, body, oedema and lethality phenotypes were fully penetrant in mutants by the equivalent of 5 dpf, whereas their siblings were unaffected (S3C Fig). Conversely, rearing at the lower permissive temperature of 20.5 ºC supressed these gross morphological phenotypes and mutants survived beyond 5 dpf-equivalent (7 days). When such surviving mutants were subsequently reared through the nursery at 26.5 ºC in tanks with their non-mutant siblings, approximately half survived to 3 months of age (S3D Fig; n = 423 survivors, Χ^2^ *p* =6 x 10^-6^). Husbandry and legal considerations prevented rearing at 20.5 ºC beyond 5 dpf-equivalent. Surviving mutant females bred poorly. Nevertheless, some maternal-zygotic (MZ) *yap1* mutant larvae obtained, when reared at the permissive temperature until 7 days, were motile and survived to adulthood. Thus, *yap1* is dispensable for early myogenesis.

Failure to thrive in the nursery and breed might reflect poor muscle function leading to low food intake. So we investigated muscle at pre-feeding stages by analysing muscle growth in MZ*yap1*^*kg137*^ mutants or MZ*yap1*^*kg137/kg152*^ trans-heterozygotes on a plasma membrane-EGFP reporter background (Fig 3). Analysis of myotome volume and muscle fibre number revealed no defect in maternal-zygotic (MZ) *yap1* mutants compared to their heterozygous siblings (Fig 3A). Even mutants showing oedema grew muscle normally, despite becoming sick and necrotic at 5 dpf (Fig 3A). To avoid the problem of the early oedema and other phenotypes, *yap1*^*kg151*^ mutant embryos were grown at the permissive low temperature from 70% epiboly to the prim 10 stage (3 days of incubation; see Materials and Methods for explanation of rearing and staging at low temperatures) and then shifted to the restrictive higher temperature during early larval muscle growth. At 5 days of incubation, the equivalent of 4 dpf stage, no difference in myotome growth was detected between *yap1*^*kg151*^ mutants and their siblings (Fig 3B). At 7 days of incubation, 5 dpf-equivalent, no defects in sarcomere structure were observed either in live larvae or after immunodetection of α-actinin (S4 Fig). *Wwtr1* mRNA was not detectably upregulated in *yap1*^*kg151*^ mutants, either in larvae or in adult muscle (S2H Fig). Thus, Yap1 is not required for muscle growth at pre-feeding stages.

To ask if the lack of Yap1 affects muscle function, we next looked at *yap1*^*kg151*^ mutants using the two movement assays. Siblings from *yap1*^*kg151/+*^ in-crosses were reared at the permissive temperature until 6 days (4 dpf-equivalent) and measurement of swim velocity in response to touch revealed that *yap1*^*kg151*^ mutant swam slower than their siblings (*p* ≤ 0.04; Fig 3C; hereafter fish grown at 20.5ºC from 70% epiboly to 5 dpf-equivalent and then transferred to 26.5ºC are staged in days, not dpf). At 7 days, maximal evoked trunk movement in *yap1*^*kg151*^ mutant fish was also reduced compared to siblings (*p* ≤ 0.003; Fig 3D). Neither defect could be accounted for by altered size of mutants (S5A and S5B Fig). When *yap1*^*kg151*^ mutants were assayed for swim speed at 13 days the defect was less apparent (Fig 3E). One lay of *yap1* mutants suggested persistence of a movement defect, whereas another lay did not (Fig 3E). We conclude that *yap1* mutants have significant motility defects in early larval stages that appear more severe than those in *wwtr1* mutants.

### Cooperativity between *Yap1* and *Wwtr1*

Given the transient nature of the motility defects in *yap1* and *wwtr1* single mutants, we asked if these paralogous genes have redundant function. As reported previously with other alleles, *yap1*^*kg151*^*;wwtr1*^*kg169*^ double mutants were not viable, showing a failure of tailbud outgrowth with misshapen somites (S6A Fig), and death at around 1 dpf, which is suggested to arise from a periderm defect [79]. Analysis of muscle development in double mutants did not reveal any defect in myogenesis, either when analysing expression of MRF genes at the 16 somite stage (16ss; S6B Fig), or in terminal differentiation and morphogenesis of slow and fast muscle at 18ss (S6C Fig). At the 24ss, shortly before their death, fast muscle tissue was well differentiated although of aberrant morphology in *yap1*^*kg151*^*;wwtr1*^*kg169*^ double mutants (S6D Fig). Thus, despite their early and sequential expression within the myogenic lineage, neither Yap1 nor Wwrt1 is required for early myogenesis.

We next asked if loss of one allele of *wwtr1* or *yap1* on a null mutant background of the other gene would affect the muscle growth at later stages. Even when reared at the restrictive temperature, at 2 dpf no difference in myotome size was apparent in *yap1*^*kg151/+*^*;wwtr1*^*kg169*^, *yap1*^*kg151*^*;wwtr1*^*kg169/+*^ or its MZ *yap1*^*kg151*^*;wwtr1*^*kg169/+*^ counterpart (S6E and S6F Fig). MZ analysis could not be performed on *wwtr1* mutants because eggs from *wwtr1* mutant females could not be fertilised, as previously suggested [88]. Despite their good initial myogenesis*, yap1*^*kg151*^*;wwtr1*^*kg169/+*^ fish showed more severe and earlier defects than their *yap1*^*kg151*^ siblings elsewhere in the body (S3A Fig), and *yap1*^*kg151/+*^*;wwtr1*^*kg169*^ larvae developed mild cardiac oedema and failed to survive to adulthood (S3A and S3F Fig). Thus, *yap1* and *wwtr1* show partial genetic redundancy.

### *Yap1* mutant fish develop kyphoscoliosis

**Fig 4:**
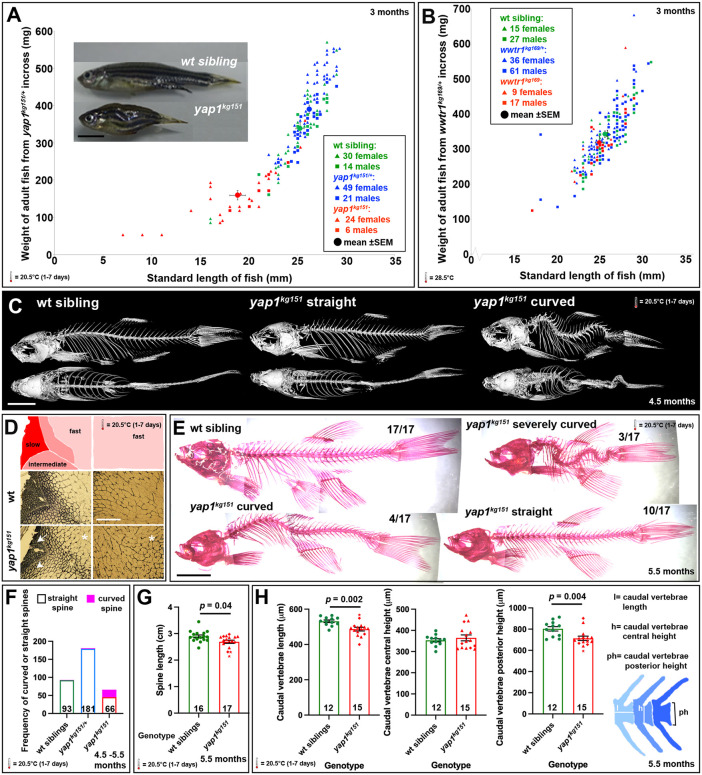
Adult *yap1*^*kg151*^ have kyphoscoliosis and/or smaller vertebrae. Heterozygous in-crosses of *yap1*^*kg151/+*^ reared at the permissive 20.5ºC temperature (A,C-H) or *wwtr1*^*kg169/+*^ reared to 28.5ºC (B) until 5 dpf-equivalent and then at 26.5ºC to the indicated age, points plotted with jitter to avoid overlap. **(A)** Reduced growth of *yap1*^*kg151*^ mutants. Some *yap1*^*kg151*^ mutant individuals showed a dramatic tail truncation not observed in non-mutant (inset). **(B)** At 3 months, adult *wwtr1*^*kg169*^ mutants have similar weight and length to their wild type and heterozygous siblings but survive poorly (Χ^2^, n = 165, *p* = 0.02). **(C)** Computerised Tomography (CT) scans of adult fish at 4.5 months viewed from lateral and dorsal. **(D)** NADH tetrazolium reductase stain showing comparable oxidative/slow (arrows), intermediate (arrowheads) and glycolytic/fast fibres (asterisks) between wild type and *yap1*^*kg151*^ mutant muscle in superficial (left) and medial (right) myotome transverse cryosections taken at 6 months. **(E)** Lateral view of wholemount alcian red stained 5.5 month old skeletons. **(F)** Frequency of curved spine within sibling 4.5-5.5 month adult fish is greater in surviving mutants from four separate *yap1*^*kg151/+*^ in-crosses compared to their siblings (Χ^2^, n = 340, *p* = 1 x 10^-11^). **(G)** Spine length of 5.5 month sibling wt and *yap1*^*kg151*^ fish. **(H)** Caudal vertebrae length, height and posterior height of 5.5 month straight and curved *yap1*^*kg151*^ fish. Statistics show significant results of Kruskal-Wallis tests with Dunn’s post hoc comparisons **(G,H)**. Bars: A,C and E = 5 mm; D = 100 μm. Number of fish analysed is indicated on columns.

To investigate the role/s *yap1* and *wwtr1* play in later development, surviving single mutants were assessed further (Fig 4). Adult *yap1*^*kg151*^ mutants were significantly shorter and lighter than their siblings, although Fulton’s condition factor (*k* = Weight/Length^3^; [89] was not reduced in mutants (Figs 4A and S6C). A similar size reduction was observed in *yap1*^*kg137*^ and *yap1*^*kg152*^ mutants (S7B and S7D Fig). In contrast, the length, weight and *k* factor of *wwtr1*^*kg169*^ mutants were similar to their siblings (Figs 4B and S7A). Microtomographic (CT) examination of *yap1*^*kg151*^ siblings raised at the permissive temperature for 7 days (5 dpf-equivalent) revealed severe kyphoscoliosis in some surviving adult *yap1*^*kg151*^ mutants (Fig 4C). Histological analysis revealed no obvious muscle defect in *yap1* mutants, whether kyphoscoliotic or not (Fig 4D).

To assess further the kyphoscoliotic phenotype in *yap1*^*kg151*^ mutants, a lay was raised to 5.5 months and kyphoscoliosis was again observed in some mutants (Fig 4E). From 86 genotyped fish, all 17 surviving *yap1*^*kg151*^ mutants and 17 wildtype siblings were closely analysed by alizarin red staining; 3/17 mutant fish had a severe kyphoscoliotic phenotype and 4/17 fish were less severely curved (Fig 4E). Ten mutant fish were straight and similar to their wildtype siblings, although in general shorter in length (Fig 4E). About a third of mutants died before adulthood (Figs 4E,4F and S3D; n = 128 surviving mutants out of 763 fish of 3 to 5.5 months of age, Χ^2^ *p* = 4 x 10^-6^) and about a third of these surviving *yap1* mutants exhibited kyphoscoliosis (n = 21/66 mutants, Χ^2^ *p* = 1 x 10^-12^; Fig 4E and 4F). Commensurately, whole spine length and vertebral length measured in the caudal region was shorter in the straight *yap1*^*kg151*^ mutants (Fig 4G and 4H). The dorsoventral height of the posterior end of the vertebral centrum was reduced in mutants (Fig 4H). Thus, loss of Yap1 function leads to a variety of vertebral phenotypes including smaller vertebrae which, in some individuals, develops into a strong kyphoscoliosis and failure to thrive.

**Fig 5:**
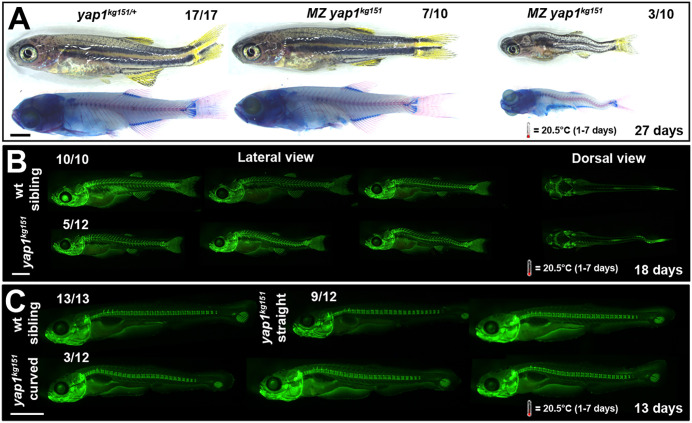
Kyphoscoliosis arises in *yap1*^*kg151*^ mutants from 13 days. **(A)** Larval fish at 27 days deriving from a female *yap1*^*kg151*^ x male *yap1*^*kg151/+*^ cross reared at 20.5 ºC until 7 days (5 dpf-equivalent) and then at 26.5 ºC. Wholemount fish before (upper) and after (lower) skeletal stain for bone (red) and cartilage (blue). Note reduced dorsoventral extent in severely kyphoscoliotic mutant (right). **(B,C)** Several examples of larval fish deriving from a *yap1*^*kg151/+*^ in-cross reared at 20.5 ºC until 7 days and then at 26.5 ºC stained live with calcein. Lateral views of 18 days (B) and 13 days **(C)**. Numbers indicate fraction of fish of each genotype with morphology shown. Bars = 1 mm.

To ask if kyphoscoliosis was more penetrant in MZ mutants, a lay from a *yap1*^*kg151*^ mutant female with a heterozygous *yap1*^*kg151/+*^ male were reared at the permissive temperature until 5 dpf-equivalent and then at 26.5ºC until 27 days. Kyphoscoliosis occurred at a similar rate to zygotic mutants, with 3/10 MZ*yap1*^*kg151*^ mutants exhibiting a bent spine (Fig 5A), suggesting that maternal Yap1 does not protect zygotic *yap1* mutants from kyphoscoliosis.

When the forming spine of larval fish raised at the permissive temperature was analysed with the live bone stain calcein [90], kyphoscoliosis was apparent in 5/12 mutants at 18 days (Fig 5B), developing from a more mild kyphoscoliosis that was already visible in some larvae at 13 days (Fig 5C). We conclude that about a third of *yap1*^*kg151*^ mutants develop kyphoscoliosis from as early as 13 days (11 dpf-equivalent).

### Investigating the cause of kyphoscoliosis

Three reported causes of kyphoscoliosis in zebrafish are defective notochord development, alterations of cerebrospinal fluid flow in the neural canal and perturbation of the Reissner’s fibre. We investigated each but failed to find evidence of defects in *yap1* mutants (S8–S10 Figs).

Multiple mutant zebrafish lines that have disorganised notochord cells develop kyphoscoliosis [91–93]. *Yap1* mRNA is present early within the notochord (Fig 1E–1G). We therefore examined *yap1*^*kg151*^ mutants reared at the permissive temperature until 3 days (48 hpf-equivalent). No defects were noted in notochords of six mutants examined by compound microscopy for size and vacuolation at 3 dpf (S8 Fig), nor in >200 embryos resulting from four separate *yap1*^*kg151/+*^ in-crosses. Vertebral centrum defects frequently arise in mutants with notochord defects [94]. Our observations at 13 days failed to reveal defects in vertebral centrum formation (Fig 5C), further arguing against an early notochordal origin for the kyphoscoliosis.

Murine *Yap1* deficiency causes hydrocephalus [95]. Defective cilia in cerebral ventricles lead to abnormal flow of cerebrospinal fluid that, in turn, has been suggested to cause both hydrocephalus and kyphoscoliosis [96,97]. Scanning electron microscopy of the rhombencephalic ventricle of kyphoscoliotic and non-kyphoscoliotic *yap1*^*kg151*^ mutants and their wild type siblings, failed to reveal either hydrocephalus or ciliary defects (S9 Fig). We conclude that Yap1-dependent kyphoscoliosis is not accompanied by a visible defect in ventricular ciliary morphology.

We did not examine ciliary function in the spinal canal, which is required for proper Reissner’s fibre formation. Kyphoscoliosis can arise in zebrafish with defects in the Reissner’s fibre, located within the spinal canal [98,99]. However, immunodetection of the Reissner’s fibre revealed its presence at the 5 dpf-equivalent stage in *yap1* mutants grown at the permissive temperature (S10 Fig).

**Fig 6:**
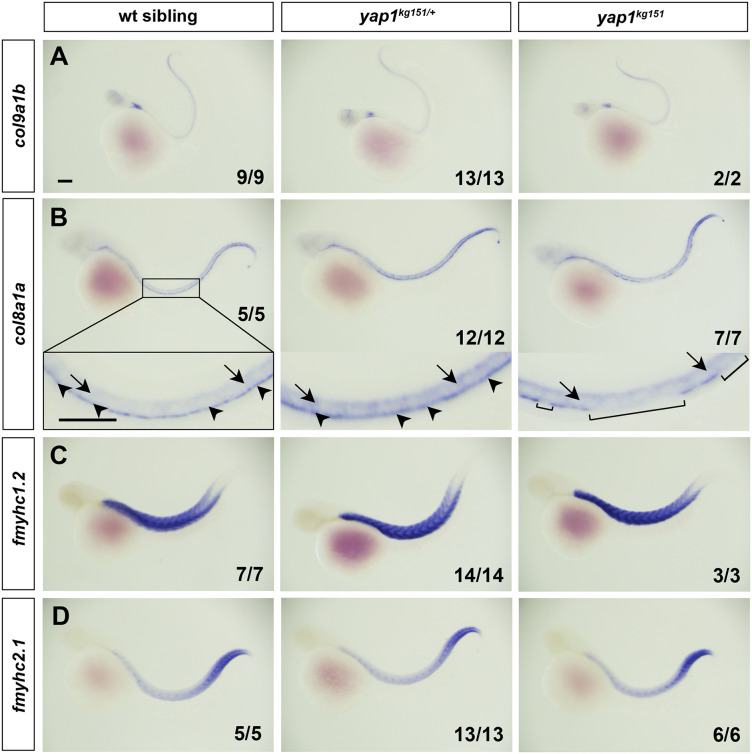
Altered hypochord marker distribution in *yap1*^*kg151*^ mutant. In situ mRNA hybridisation for collagen genes implicated in kyphoscoliosis and fast myosin heavy chain genes involved in axial patterning in sibling embryos reared from a *yap1*^*kg151/+*^ in-cross at non-permissive temperature (28.5ºC) that were photographed and subsequently PCR genotyped. **(A,B)** Collagen extracellular matrix genes *col9a1b* (A) and *col8a1a* (B) are unchanged in 36 hpf notochord (arrows) of mutants compared to their siblings. *Col8a1a* mRNA is abundant in sibling hypochord throughout the axis (B, arrowheads, shown magnified in insets), but lacking in several segments in mutants (B, brackets). Additional *col8a1a*-stained embryos of each genotype are shown in S11 Fig. **(C,D)** In contrast, the axially-patterned localisation of fast myosin heavy chains *fmyhc1.2* (D, trunk) and *fmyhc2.1* (E; tail) is unaltered at 48 hpf. Numbers of (individuals with the pattern shown)/ (total genotyped individuals) are displayed on each panel. Bars = 100 μm.

Another cause of kyphoscoliosis in zebrafish is loss of Dstyk activity, which also reduces notochordal collagen expression and shortens the body axis by 2 dpf [100]. We did not observe either a shortening of the body axis or reduction in notochordal *col9a1b* or *col8a1a* mRNA in *yap1*^*kg151*^ mutants (Fig 6A and 6B). However, *yap1*^*kg151*^ mutants did display focal losses of strong *col8a1a* mRNA accumulation in the hypochord in all seven mutants genotyped (Figs 6B and S11). Other genes, such as fast myosin heavy chain genes *fmyhc1.2* and *fmyhc2.1*, which are regionally expressed along the body axis, showed no defects (Fig 6C and 6D). We conclude that Yap1 is essential for normal patterning of hypochord development but how this early phenotype relates to later growth defects and kyphoscoliosis is unclear.

### Larval growth requires *Yap1*

**Fig 7:**
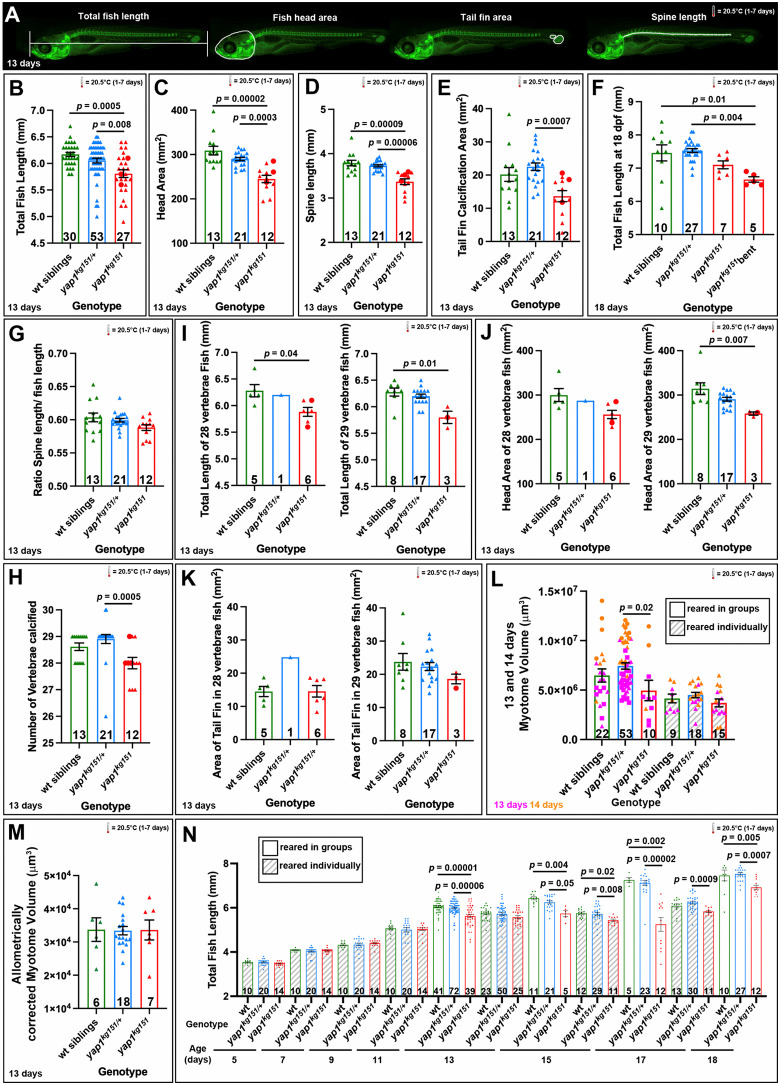
*Yap1* is required for normal larval growth in a group. *Yap1*^*kg151/+*^ in-crosses were reared at 20.5°C from 70% epiboly to 7 days (5 dpf-equivalent) and then at 26.5°C until 13 days (11 dpf-equivalent) or 18 days (16 dpf-equivalent). Larvae were stained with calcein. **(A)** Schematics of measurements made (white). **(B-G,H,M,N)** Total fish length (B,F,N), head area **(C)**, spine length **(D)**, tail fin calcified area **(E)**, Spine length divided by fish length ratio **(G)**, number of calcified vertebrae (H) and myotome 17 volume/total fish length cubed **(M)**. **(I,J,K)** After segregation into groups containing 28 or 29 calcified vertebrae, total fish length (I) and head area **(J)**, but not tail calcified area **(K)**, were reduced in *yap1* mutants compared to wt siblings. **(L)** Volume of myotome 17 in 13-14 day fish reared in group nursery environment (clear bars) or individually with unlimited food (striped bars). **(N)** Length of fish at 5, 7, 9, 11 and 13, 15, 17 and 18 days reared individually with unlimited food (striped bars) or in group nursery environment (clear bars). Statistics: Kruskal-Wallis tests with Dunn’s post hoc comparisons. Green bars wt siblings, blue bars *yap1*^*kg151/+*^ siblings red bars *yap1*^*kg151*^. Red circle symbols represent curved spine fish (B-F,H-K). Symbol shape represents different lays/experiments **(L,N)**. Number of fish analysed is indicated on columns.

Growth of *yap1* mutants was studied in more detail. Fish from a *yap1*^*kg151/+*^ in-cross were reared at the low permissive temperature until 7 days to allow early survival and their growth under optimal husbandry conditions followed at 26.5ºC in our professional aquarium. *Yap1*^*kg151*^ mutant fish analysed at 13 days were already smaller in total fish length, spine length, head size and tail fin calcified area (Fig 7A-7E). Although fish with bent spines were not particularly short at 13 days (Fig 7B), by 18 days scoliotic fish were amongst the shortest in the lay (Figs 5B and 7F). At 13 days, spine and total fish lengths were reduced similarly (Fig 7G). Thus, Yap1 is required for normal growth of the larval skeleton.

*Yap1*^*kg151*^ mutant fish appeared slightly delayed in development, as mutants had on average one fewer calcified vertebrae than their siblings (*p* = 0.0005 Mann-Whitney; Fig 7H). When larvae were stage-matched according to their number of calcified vertebrae, *yap1*^*kg151*^ mutants still had a growth defect compared to their siblings, with shorter total length and head area (*p* ≤ 0.02 Mann-Whitney; Fig 7I and 7J). These reductions were not more pronounced in bent compared to straight mutants (Fig 7I and 7J compare circle and triangle symbols). In contrast, tail fin calcification appeared unchanged after stage-matching mutants and siblings (Fig 7K), suggesting this feature is indirectly dependent on Yap1. These findings suggest that *yap1* mutants have a gross skeletal growth defect independent of a subtle developmental delay.

The skeletal reduction in *yap1*^*kg151*^ mutants was accompanied by defects in muscle growth. The myotome volume measured between 13 and 14 days was reduced in mutants housed with their siblings (*p* = 0.01 Mann-Whitney; Fig 7L). Fish housed alone grew more slowly despite ad libitum feeding and had yet to show a muscle defect at the same age (Fig 7L). Although the *yap1*^*kg151*^ mutant fish reared in groups had smaller myotome volumes, this size difference was removed if we divided it by body length cubed, to measure their overall condition [101,102], an analogue of Fulton’s *k*; Fig 7M). Therefore, despite a clear skeletal defect in *yap1* mutants, there is little indication of a disproportionate muscle defect that might account of the skeletal changes.

To ask when the growth defect arose, fish reared under the permissive low temperature regime until 5 dpf-equivalent (7 days) were measured at 5, 7, 9, 11 and 13 days. Under standard husbandry conditions, zebrafish get nutrients entirely from their yolk for the first 5 dpf, at which point they start to feed but yolk is not fully consumed until at least 10 dpf. After the first length measurement, the fish were raised in a light cycling incubator at 28.5°C in individual 35 mm wells in 6 well plates to enable longitudinal study of each fish. Fish were fed rotifers as they would be in the aquarium from 7 days. Surprisingly, these fish remained small and *yap1*^*kg151*^ mutants showed no difference in length or muscle volume from their siblings even at 13 days, nor did any have kyphoscoliosis (Fig 7L and 7N). However, when the experiment was repeated and fish raised individually through 15 and 17 days, *yap1*^*kg151*^ mutants began to show a growth defect (Fig 7N). We conclude that Yap1 is required for normal larval growth during the mid-larval period.

## Discussion

The current study reports three novel findings. Firstly, that *yap1* loss of function mutation causes a variably penetrant kyphoscoliosis arising in early larval life. Secondly, that *yap1* mutation leads to a generalised growth defect that becomes apparent even in morphologically normal individuals at the mid-larval stage. Thirdly, that *wwtr1* function is required for optimal survival to adulthood even in a protected aquarium environment. Our study also confirms several observations made with distinct *yap1* and *wwtr1* mutant alleles in zebrafish, including a striking temperature sensitivity of the *yap1* loss of function phenotype.

### Temperature sensitivity of *yap1* putative null alleles

Four separate *yap1* early-truncating mutant alleles occurring in distinct exons and isolated in different laboratories, *yap1*^*sa25458*^, *yap1*^*mw48*^, *yap1*^*zf2210*^ and *yap1*^*kg151*^, have now shown temperature sensitivity, with higher temperatures being non-permissive [86,87,103]; the current study). Although none of these alleles is a deletion, they are likely to reveal the null phenotype. *Yap1*^*kg151*^ causes translational termination early in the first coding exon, shows nonsense-mediated mRNA decay, and lacks evidence of genetic compensation. Moreover, as previously reported with other *yap1* alleles that lack Yap1 immunoreactivity, at the non-permissive temperature *yap1*^*kg151*^;*wwtr1*^*kg169*^ dual loss of function is embryonic lethal during late somitogenesis stages, whereas loss of *yap1* function alone leads to cardiac and yolk sac oedema, small eyes and death in the early larval stage in both zygotic and MZ mutants [86,79]. At the permissive temperature, almost all *yap1*^*kg151*^ mutants appear wild type at 5 dpf. Loss of *wwtr1* function, on the other hand, leads to viable adults despite the reported cardiac and other defects [48,79,86,88,104–106].

Temperature sensitivity of null mutants has been reported in bacteria and yeast [107–109]. In animals, however, *ts* alleles are generally missense single amino acid substitutions affecting protein folding [110]. Interestingly, a truncating loss of function mutation (possibly not null) in *scrib*, which interacts genetically with *yap1*, is also temperature sensitive [87]. We suggest that temperature-sensitivity of unknown modifier gene/s or physiological processes account for variable penetrance of *yap1* mutant alleles. The role of Yap1 in the response to environmental temperature merits further investigation.

### *Yap1* is required for larval motility and growth

Normal Yap1 function is essential to avoid early developmental defects, including oedema and eye defects. When *yap1*^*kg151*^ mutant fish are grown at non-permissive temperatures, the larvae do not survive. Pericardial oedema is frequently attributed to cardiovascular defects and Yap1 has been extensively implicated in cardiac development [111–115]. Pericardial and yolk-sac oedema has also been attributed to kidney or liver defects [116,117] and *yap1* has been reported to play roles in both kidney and liver development [118–120]. Interestingly, the earliest defect observed in *yap1*^*kg151*^ mutants is an uneven axial distribution of *col8a1a* mRNA in the hypochord, an axial midline structure underlying the notochord. Hypochord signals, such as Vegfc, have been implicated in lymphangiogenic and vascular patterning [121], defects in which might contribute to the cardiovascular phenotype of mutants. However, gross vascular flow or morphology defects were not observed at 36 hpf; further study is warranted. Thus, the data are consistent with roles of Yap1 in early development of a variety of organs.

When grown at the low permissive temperature until the 5 dpf-equivalent stage, fish lacking zygotic Yap1 have a motility defect but normal body and muscle size and, even when shifted to non-permissive conditions at 5 dpf-equivalent, some go on to survive to adulthood. Nevertheless, following Yap1 inactivation from 5 dpf-equivalent, *yap1*^*kg151*^ mutants show an early growth deficit, which amounts to about a 10% reduction in length, head and tail size. A subset of such *yap1* mutants show no morphological or obvious behavioural defects, but have a slight delay in vertebral calcification and growth. These ossification defects correlate with the reduction in body length. Various murine *Yap1*/*Wwtr1* mutant combinations show reduced overall growth [55], as we observe in *yap1* mutants. The findings suggest that the growth defect in *yap1*^*kg151*^ mutants arises as early as 13 days and persists into adulthood, accompanied by poor survival.

The mechanism underlying the motility defect is unclear because Yap1 protein is expressed in multiple tissues. Histological and size analysis of the *yap1* mutants at early larval stages show myotome size and cellularity are similar to wild type siblings, even though early motility is required for muscle growth [19]. Although the function of larval muscle is poor, adult muscle appears histologically normal except for the reduced size of the fish. TEAD transcription factors can mediate Yap1 effects on transcription and a recently isolated *tead1a* mutant appeared ‘slimmer’ than wild type; however scoliosis was not reported [122]. Resolution of the cell autonomy and mechanism of Yap1 action will await tissue-specific genetic manipulation.

### *Yap1* helps maintain vertebral column symmetry

Yap1 is required to ensure symmetrical vertebral column morphogenesis. A kyphoscoliotic spinal phenotype of variable severity arises when mutant larvae are grown at the permissive temperature until 5 dpf-equivalent stage and the non-permissive temperature thereafter. Whereas the only early cartilage or bone defect in the backbone of larvae was a slight delay in calcification, some individual mutants showed a mildly curved spine at 13 days and by 1 month of age kyphoscoliosis was apparent in around a third of zygotic *yap1* mutants. Similar defects were not observed in *wwtr1*^*kg169*^ mutants, *yap1*^*kg151/+*^ heterozygotes or *yap1*^*kg151/+*^*;wwtr1*^*kg169/+*^ dual heterozygotes even if grown at the non-permissive temperature throughout their development. Analysis of *yap1* MZ mutants revealed a similar phenotype to the zygotic mutants; about a third of MZ*yap1*^*kg151*^ mutants showed kyphoscoliosis, suggesting that the maternal contribution does not affect the penetrance of the zygotic phenotype.

Yap1 has been extensively implicated in mechanosignalling [50,123,124]. A correlation between mechanical influences and kyphoscoliosis has long been proposed [34,35,37–39,125]. Once an initial defect in the spine occurs, generally from an unknown cause, worsening of the defect often occurs during adolescent growth spurts. In the 1880s, this observation engendered a hypothesis, the Hueter-Volkmann ‘Law’, which stated that vertebral growth is retarded by mechanical compression of the growth plate and stimulated by reduction of compression [34,39] thereby explaining the exacerbation of scoliosis during growth under asymmetric gravitational load. Studies on rat tails show that mechanical force can modulate or correct vertebral growth [35]. The kyphoscoliosis observed in the *yap1*^*kg151*^ mutants is first observed from 13 days and gradually worsens in some individuals, during zebrafish adolescence [126]. However, this process cannot be attributed to gravitational positive feedback effects because larvae are neutrally buoyant. Perhaps, in the absence of *yap1*, mechanical force is not transmitted or sensed correctly along the spine, causing a curve that, once present, progresses to kyphoscoliosis in about a third of the mutant fish.

About a third of *yap1*^*kg151*^ mutants also die during rearing. We note, however, that the frequency of kyphoscoliotic mutants did not obviously decrease with age, suggesting either that mutants die for reasons unrelated to kyphoscolioisis or that, as kyphoscoliotic mutants perish, additional mutants develop kyphoscoliosis during their adolescence.

As with humans, the heterogeneous genetic background in zebrafish *yap1* mutants may explain the variable penetrance and time of onset of kyphoscoliosis. Alternatively, subtle epigenetic or environmental variables may affect the expression of the *yap1* mutant phenotype. Such effects may account for the variable survival rate between different lays.

### Poor larval survival without *Wwtr1*

Zebrafish *wwtr1*^*kg169*^ mutants, like those previously reported [86,88,120,127–129] do not recapitulate the failure to undergo skeletal calcification reported in *wwtr1* morphants [74]. This is in contrast to murine manipulations in which *Wwtr1* mutant mice are born significantly smaller than siblings with minor defects in ossification [55].

Despite the viability and unaltered size of zebrafish *wwtr1*^*kg169*^ mutants, they survive less well than their siblings, even in the protected environment of an aquarium. The *wwtr1*^*bns35*^ mutant allele has been shown to cause defects in cardiac trabeculation [128], which might enhance larval death. Mice lacking Wwtr1 die between birth and weaning, possibly from kidney defects [55,71]. As we confirm that *yap1;wwtr1* double mutants show lethally severe early defects, it seems that balance of functional requirement for Yap1 and/or Wwtr1 in individual tissues has varied during vertebrate evolution. We also note that *yap1*^*kg151/+*^ heterozygosity may prevent survival of *wwtr1*^*kg169*^ mutants. Clearly, some Wwtr1 function(s) may be essential for effective survival in the wild.

### Cell autonomy of *Yap1* action in kyphoscoliosis unresolved

As we have analysed putative null *yap1* and *wwtr1* mutations, the cellular origin of the observed defects remains unknown. All components of the neuromusculoskeletal system, nerve, muscle, tendon, cartilage, bone and associated vessels, or a combination of them, are candidates for the site(s) of action of Yap1. Despite their early poor motility, the earliest putative precursor to kyphoscoliosis that we could discern in *yap1*^*kg151*^ mutants was at 11–13 days in spinal bone itself, where a delay in calcification was accompanied by mild spinal curvature in some individuals. Vertebrae arise from cells of the notochord sheath, somitic sclerotome and neural crest, but no defects in these tissues were observed at earlier stages. An osteogenesis imperfecta (OI) bone phenotype correlating with the extent of reduction of Yap1 and Wwtr1 in bone precursors has been reported in murine conditional mutants [56]. Murine cartilage-specific deletion of *Yap1* and *Wwtr1* has also shown subtle morphological bone defects [59]. Moreover, Yap1 and Wwtr1 have opposing effects at different stages of limb osteoblast differentiation [60]. Thus, vertebral precursor cells remain prime candidates for a cell autonomous origin of the defect.

The striking segmental interruptions of the hypochord observed in 36 hpf *yap1* mutants suggest a possible vascular or lymphatic origin for kyphoscoliosis. We note, however, that the hypochord defect did not cause any obvious vessel defects and was fully penetrant at the non-permissive temperature. Further studies are required to determine whether and how similar defects in a subset of embryos reared at the permissive temperature might contribute to either the motility defect or the later vertebral anomalies and kyphoscoliosis.

Many zebrafish scoliotic phenotypes arise from defects in early notochord vacuolation, elongation and extracellular matrix deposition that lead to subsequent defective of ossification [100,130]. *Yap1* mutants lack the poor notochord vacuolation and body length reduction at embryonic stages and calcein vertebral centrum defects at 15–20 dpf observed in *dstyk* and MZ*ptk7a* mutants and *vangl2* knockdowns. Thus, the *yap1* vertebral column phenotype arises at the mid larval stage after apparently normal early notochord formation.

The early motility defect is also a possible cause of kyphoscoliosis. Defects arising in the central nervous system are poor candidate kyphoscoliosis triggers because *yap1* mutants showed early motility defects in response to direct electrical stimulation, which is thought to act peripherally [131]. So altered muscle-derived force was a prime trigger candidate, particularly because severe craniofacial cartilage and bone defects can arise from early muscle defects caused by mutation of genes acting cell autonomously in muscle [12]. Given the strong and sequential expression of *yap1* and *wwtr1* during myotomal myogenesis and evidence that Yap1 can affect murine myogenesis [58,67,132,133], we hypothesised that early muscle defects in *yap1* mutants might cause force asymmetry leading to kyphoscoliosis. However, an extensive analysis of early muscle failed to reveal a morphological or histological defect in mutants, despite their poor motility. Moreover, *yap1*^*kg151*^ mutants show normal early muscle growth even when shifted to the non-permissive temperature during the early growth phase. Yap1 has also been shown to affect tendons [134–136], but given that tendons are small in 5 dpf zebrafish, we have not analysed this tissue.

Recessive variants in *MYH3*, the gene encoding the skeletal muscle-specific embryonic myosin heavy chain, cause spondylocarpotarsal synostosis (SCTS), characterised by vertebral fusions and scoliosis [137]. Ablation of the murine *Myh3* gene similarly causes severe muscle defects with altered muscle Yap1 signalling and leads to scoliosis in surviving mice [6]. Our finding that loss of *yap1* function can cause kyphoscoliosis strengthens the evidence for the involvement of Yap1 signalling in the aetiology of *MYH3*-associated SCTS and possibly other scolioses. Bharadwaj et al. further suggested that *Myh3* ablation caused muscle defects and scoliosis through Yap1 activation because both phenotypes were mitigated by CA3, a drug that can block Yap1 action [6]. These observations raise the hypothesis that inhibition of Yap1 function cell autonomously within muscle mitigates scoliosis by its clear beneficial effects on muscle development. However, *yap1* mutant fish do not show a severe muscle defect, yet have scoliosis. Although zebrafish do not contain an *Myh3* orthologue, we observed no alteration in expression of regionally-expressed *fmyhc1.2* and *fmyhc2.1* fast myosin genes that might have contributed to kyphoscoliosis. Moreover, in fish, it is loss of *yap1* function rather than gain, that promotes scoliosis. Thus, defining the cellular site and mechanism of Yap1 action required to prevent kyphoscoliosis is a priority.

Familial Kyphoscoliotic Ehlers-Danlos Syndrome arises from rare mutations in genes implicated in collagen matrix formation (*PLOD1* and *FKBP14*; [138]). GWAS studies of adolescent idiopathic scoliosis (AIS) have yielded hits near genes involved in neuromuscular (*EPHA4*/*PAX3*, *LBX1, SOX6* and *GPR126*) and skeletal (*PAX1*, *SOX9*, *TBX1*, *MEOX2*) development and in matrix biology (*BNC2, FBN1*), but to date account for a small fraction of heritability [4,139]. A YAP1-BNC2 interaction is implicated in matrix remodelling in response to mechanosignals [140]. We speculate that Yap1 function mediates mechanosignalling to regulate cell-matrix interactions in one or more tissues of the developing neuromusculoskeletal system and may contribute to some forms of AIS.

Lastly, a number of mutations affecting sensorimotor function have yielded kyphoscoliotic phenotypes in zebrafish, including some affecting cilia and the Reissner’s fibre [97,99]. While the cell types requiring this ciliary function are unclear, it has been suggested that defects in fluid circulation in the neural ventricles can underlie both Reissner’s fibre defects and scoliosis [96,98]. Although Yap1 has not been implicated in ciliary function, we investigated this possibility by analysing cilia in the ventricle and the Reissner’s fibre but failed to observe any morphological defect. Whether other neural system defects deriving from loss of Yap1 function, but acting through altered muscle or hypochord functionality, underlie kyphoscoliosis remains to be determined. While the mechanism leading to kyphoscoliosis in some individual *yap1* mutants remains unclear, we hypothesise that Yap1 may act to suppress positive mechanofeedback leading to asymmetric bone growth.

## Materials and methods

### Ethics statement

All experiments were performed in accordance with licences held under the UK Animals (Scientific Procedures) Act 1986 and later modifications after approval by King’s College London Animal Welfare and Ethical Review Board and the UK Home Office and conforming to all relevant guidelines and regulations.

### Zebrafish lines and maintenance

Genetically-altered *Danio rerio myf5*^*hu2022*^ [83], *myf6*^*kg126*^ (new, 3 bp deletion creating STOP (UAG) at position aa34: predicted null), *myod1*^*fh261*^ [12,141], *myog*^*kg125*^ [81], *wwtr1*^*kg133*^ (new, TALEN: 1 bp deletion T83, creating 4 aa nonsense tail then STOP; predicted null), *wwtr1*^*kg169*^ (new, TALEN: 8 bp deletion 82–89 CTTTTTAA, 105 aa nonsense tail; predicted null), *yap1*^*kg137*^ (new, TALEN: 19 bp deletion 106–124 AAAAACACCATCGTCCCCC, 4 aa nonsense tail; predicted null), *yap1*^*kg151*^ (TALEN: 5 bp deletion 111–115 CACCA, 84 aa nonsense tail; predicted null), *yap1*^*kg152*^ (TALEN: 11 bp deletion 110–120 ACACCATCGTC, 82 aa nonsense tail; predicted null), *Tg(Ola.Actb:Hsa.HRAS-EGFP)*^*vu119*^ *(ßActin:membEGFP*; [142]), *Tg(actc1b:mCherryCAAX)*^*pc22*^ and *Tg(actc1b:LIFEACT-EGFP)*^*pc21*^ [143] were kept on AB background, reared at King’s College London on a 14/10h light/dark cycle [144]. Adults were kept at 26.5°C and embryos/larvae were kept at 28.5°C (or 20.5°C or 32.0°C where stated) in the dark until 5 dpf-equivalent stage. To avoid developmental problems, including increased death rates [145], caused by rearing fish at non-standard temperatures during early embryonic stages, all fish were reared at 28.5ºC until 70% epiboly (~8 hpf) and then shifted to alternate temperatures as described. For clarity, the age of fish reared at non-standard temperatures are described by their absolute age in days, rather than by standard dpf stages at 28.5ºC (S1 Table). Pilot experiments showed equivalent development of length, pigmentation and yolk consumption at 5 dpf @ 28.5ºC and 7 days @ 20.5ºC. Fish raised past 5 dpf-equivalent developmental stage (7 days if raised at 20.5°C) in the incubator were kept separately in 12 or 24 well plates on a 12/12h light/dark cycle fed with fresh rotifer food daily.

### Genotyping

Adult fish fin-clips or whole anaesthetised embryos/larva were dissolved in 50 μl 25 mM NaOH, 0.2 mM EDTA for 1 hour at 95°C and neutralized with 50 μl of 55 mM Tris HCl pH 8.0. *Myf5*^*hu202*^ was genotyped by PCR using primers CATTGTCTCCAATGGGCCTGCAAAGCTCG and GGATCTCTACCTTGGGGAGGCGTTG annealed at 60°C, followed by restriction digest with *TaqI* (NEB, R0149S) at 65°C for 4 hours to over-night, yielding uncut wt band (175 bp) and cut mutant bands (145 and 30 bp). *Myf6*^*kg126*^ was genotyped by PCR using primers GGGCACCAGAAGGCCTATTG and GGTTGTATGTGTAAGGGTCAGT GTC annealed at 59°C, digested with *RsaI* (NEB, R0167) at 37°C for 4 hours to over-night yielding cut wild-type bands (250 and 396 bp), uncut mutant band (646 bp). *Myod*^*fh261*^ was genotyped by PCR using primers GGACCCCAGGCTTGTTC and GTTGGATCTCGGACTGGA annealed at 56°C**,** digested with *BsaXI* (NEB, R0609) at 37°C for 4 hours to over-night, yielding uncut wild-type band (397 bp) and cut mutant bands (260, 119 and 18 bp). *Myog*^*kg125*^ was genotyped by PCR using primers TCAGAAACACCCACAAACGCTCAC and GCAGGCCCAGGGGAGACACT annealed at 54°C, digested with *EcoRV-HF* (NEB, R3195) at 37°C for 4 hours to over-night, yielding cut wild-type bands (197 and 164 bp) and uncut mutant band (361 bp). *Wwtr1*^*kg133*^ and *wwtr1*^*kg169*^ were genotyped by PCR using primers CGGCCATTTTAATCGAAGTTTGTT and CTGTAGGGACGCCGGAGATGAGC annealed at 57°C followed by sequencing (Genewiz) with the latter primer. *Yap1*^*kg137*^*, yap1*^*kg151*^ and *yap1*^*kg152*^ were genotyped by PCR using primers TCTTTTTGGGTTGTTTTGGATTA and GGCTCTGGCGGCGTGAA annealed at 57°C, followed by sequencing with the former primer. An alternative High Resolution Melting (HRM) method was also used to genotype *yap1*^*kg151*^ using the primers ACCGATCTGGAGGCTCTTTT and GTCTGGCAGCTTTCTCAACC and *wwtr1*^*kg169*^ using primers GTGATCCATGTCGCCAAAGACT and GCGGCATATCCTTGTTC. Both HRM methods used the standard 60°C annealing temperature with Melt Dr (Thermo Fisher Scientific, 4415450) reagent in an Applied Biosystems ViiA 7 with the HRM set-up in duplicate 10 μl reaction volumes.

### Generation and characterisation of *yap1* and *wwtr1* mutants

*Yap1* and *Wwtr1* mutants were generated by TALEN (Transcription activator-like effector nuclease) genome editing. Two RNA guide TALEN arms were designed to target within the first exon of each gene, 5′-TAACGCTGTGATGAACCC-3’ and 5’-CCCCTTCCGTGCCGATG-3’ (exon 1 of *yap1*) and 5′-TGGACACGGATCTGGAGG-3’ and 5’-CATGAACCCGAAACCGA-3’ (exon 1 of *wwtr1*) using Zifit software [146, 147]. TALEN arms were constructed using cloning by the REAL Assembly Plasmid Method [147]. Capped TALEN mRNAs were injected into single cell embryos to induce FokI cuts at the target site. Alleles were selected at F1 that carried mutations causing premature stop codons after a nonsense tail. All the alleles described for each gene have a similar phenotype, but *yap1*^*kg151*^ and *wwtr1*^*kg169*^ were eventually favoured due to their high levels of nonsense mediated decay. Repeated outcrossing to ‘wildtype’ AB did not reveal changes in phenotype to F4.

Quantitative reverse transcriptase-polymerase chain reaction (qRT-PCR) was performed on RNA extracted from four sibling individual 7–8 dpf-equivalent larval trunk/tails of each genotype (heads were used for genotyping) or from dissected adult muscle of the indicated number of genotyped sibling individuals. Larvae were sonicated on ice in 100 μL TRI Reagent (Sigma T9424) and left at room temperature for 5 minutes before 1-Bromo-3-chloropropane (50 μL, Sigma B9673 was added, incubated for 10 minutes at room temperature, microfuged at 4°C for 10 minutes and RNA purified from the aqueous upper phase by phenol/chloroform extraction. Adult muscle (~50 mg) was processed in 500 μL of TRI Reagent using a Tissue Ruptor (Qiagen 9002755) and purified using RNeasy Mini kit (Qiagen 74106). The cDNA synthesis and qPCR were performed as described [148], using primers listed in S2A Table.

### In situ mRNA hybridization (ISH), immunodetection, histology and imaging

ISH was performed as described [149]. Digoxigenin-labelled probe for *yap1* was made by PCR from 24 hpf cDNA with the primers TAATACGACTCACTATAGGGAGAACGCCGCCAGAGCCAAAGTCC and GGATCCATTAACCCTCACTAAAGGGAAGTGCCGCCATCCTGCTCCAT. Probes for *wwtr1* were made from Image Clone 9037619 (Sourcebioscience). Primers used to make probes for *col8a1a*, *col9a1b*, *fmyhc1.2* and *fmyhc2.1* are listed in S2B Table. Embryos were immersed in glycerol and imaged on a Leica MZ16F with LED light attachment, Olympus DP70 camera and DP Controller software.

Immunodetection was performed as previously described [150] with antibodies Rabbit anti-GFP (1:500, Torrey Pines, ABIN110592), A4.1025/sarcomeric myosin heavy chain (MyHC; 1:10, Abcam, ab37484), F59/slow MyHC (1:5, Santa Cruz Biotechnology, sc-32732), F310/fast myosin light chain (1:5 DSHB), Mouse anti-alpha-actinin (1:500 Sigma A7811), GαMIgG(H + L)Alexa Fluor488 (1:1000 Invitrogen A-11001), GαMIgA-FITC (1:1000 Serotec 5104-3104F), GαMIgG1AlexaFluor555 (1:1000 Invitrogen A-21127), GantiR IgG (H + L) Alexa Fluor488 (1:1000 Invitrogen A-11008) and Hoechst 33342 (1:2000 New England Biolabs 4082S). Larvae older than 2 dpf were lightly fixed with 2% paraformaldehyde in PBS for 30 minutes at room temperature. Embryos/larvae were mounted in low melting point agarose and imaged on a Zeiss LSM Exciter confocal microscope using Zeiss 20 × /1.0 NA dipping objective, analysed using Fiji (NIH, ImageJ2 2.9.0/1.53t) or ZEN 2009 (Zeiss) software. Myotome volume and fibre number measurements followed [85]. NADH tetrazolium reductase staining was performed as described [81].

Immunodetection of the Reissner’s fibre was performed as described in [99] on bleached larvae at 5 dpf-equivalent using 3 day incubation for the AFRU rabbit primary antibody (a generous gift of Dr. M. Monserrat Guerra, Universidad Austral de Chile) and 3 days for the secondary antibody. Some larvae were transected to improve access. Individuals were countrerstained with Hoechst 33342 in the secondary step.

### Skeletal assays

Alizarin Red and Alcian Blue staining was performed as previously described [151]. Measurements of spine and caudal vertebrae were performed using Image J version 1.53. Measurements on caudal vertebrae C15-C20 were averaged for each fish. Calcein staining of live fish was as described [152]. Micro Computerized Tomography (CT) scans of 4.5 month frozen fish supported by gauze were made in the Centre for Craniofacial Regenerative Biology (KCL) using a Scanco MicroCT 50 (300–350 ms exposure, 70 kV, 114 μA, 10 μm resolution).

### Scanning electron microscopy

Skulls were opened with forceps, the brain removed and bisected with a sapphire knife, fixed in 2% (v/v) glutaraldehyde in 0.1 M sodium cacodylate buffer (pH 7.3) overnight at 4°C, and processed by the KCL Centre for Ultrastructural Imaging.

### Movement assays

The larval tail movement assay was carried out as previously described [148], and tail angle of movement was measured in Fiji (NIH) software. Velocity swim assay conducted as previously described [153], recorded at early larval stages on Leica MZ16F with LED light attachment, Olympus DP70 camera and DP Controller software or with an iPhone 13. Fish velocity was measured using Tracker (http://physlets.org/tracker) software, by averaging the second and third time intervals at the early (4–5 dpf) larval stage, but the second to fourth time intervals at the later (11 dpf) larval stage because motility persisted longer than in younger larvae. The first interval was omitted as it was unknown when within it the fish began to move.

### Statistics

Unless otherwise stated, parametric and non-parametric tests appropriate to the data were performed using GraphPad Prism version 10.6.1. Χ^2^ tests were performed in Excel for Mac 16.101.3. Raw count data underlying the current work is available in S1 Data.

## Supporting information

S1 Fig *Yap1* and *wwtr1* mRNA accumulation is altered in *myod1* mutants. In situ mRNA hybridisation for *yap1* mRNA (A,C,E,G) and *wwtr1* mRNA (B,D,F,H) in genotyped mutant and wild type (wt) sibling embryos from single lays from heterozygote in-crosses of MRF mutants *myod1*^*fh261*^, *myog*^*kg125*^, *myf5*^*hu2022*^ and *myf6*^*kg126*^. Lateral whole mounts, anterior to left, dorsal to top are magnified at right. At lower right, transverse cryosections in the yolk-extension region have dorsal to top and show somitic muscle tissue (yellow dots). **(A)** *Yap1* mRNA is up-regulated in superficial somitic regions (arrowheads) of *myod1*^*fh261*^ mutants (*myod1*^*fh261*^ vs wt sib *p* = 0.01, *myod1*^*fh261*^ vs *myod1*^*fh261/+*^ *p* = 0.012, wt sib vs *myod1*^*fh261/+*^ *p* = 0.877, Kruskal-Wallis, adjusted with Bonferroni-Holm). **(B)** *Myod*^*fh261*^ mutants have similar intensity but lesser extent of somitic *wwtr1* mRNA signal in comparison to wt siblings. **(C-H)** *Yap1* mRNA in *myog*^*kg125*^, *myf5*^*hu2022*^ and *myf6*^*kg126*^ mutants appears comparable to wt siblings (C,E,G). *Wwtr1* mRNA accumulated less in *myog*^*kg125*^ mutant somites in comparison to wt sibling (D). *Wwtr1* mRNA in *myf5*^*hu2022*^ and *myf6*^*kg126*^ was indistinguishable from wt (F,H). Fractions represent number of embryos with phenotype shown/numbers genotyped, in all cases heterozygotes appeared wt. Bars = 100 μm.
(PDF)

S2 Fig Genome edited *yap1* and *wwtr1* loss-of-function mutants. **(A,B)** Schematics of *yap1* (A) and *wwtr1* (B) protein, mRNA and gene showing location of TALEN binding sites in exon 1 and primers used to identify and genotype mutants. **(C-G)** Mutant alleles showing schematic of protein truncation (top left, red indicates length of predicted nonsense C-terminal peptide), sequencing traces of heterozygote (top right) and in situ mRNA hybridisation showing nonsense-mediated decay in *yap1*^*kg151*^ (C), *yap1*^*kg152*^ (D) and *wwtr1*^*kg169*^ (E), but not *yap1*^*kg137*^ (F) or *wwtr1*^*kg133*^ (G). Fractions show number of each genotype among unsorted embryos and how blind sorting of the three ISH phenotypes was confirmed with 100% genotype accuracy in *kg151*, *kg152* and *kg169*, but genotypes could not be distinguished by ISH in *kg137* or *kg133*. Bars = 100 μm. **(H)** qRT-PCR for *yap1* or *wwtr1* mRNA in *wwtr1*^*kg169*^ and *yap1*^*kg151*^, respectively, in 5 dpf-equivalent larval trunk/tail (left and centre) or adult myotomal muscle (right). Symbol shapes indicate siblings. Mean ± SEM, t-test statistics.
(PDF)

S3 Fig Variable penetrance of severe *Yap1* and mild *Wwtr1* mutant phenotypes. **(A)** Dual heterozygote *yap1*^*kg151/+*^*; wwtr1*^*kg169/+*^ in-cross larvae reared at 28.5ºC in lateral view, dorsal to top, anterior to left. *Yap1*^*kg151/+*^*;wwtr1*^*kg169*^ fish have oedema (asterisks) from 2 dpf. In comparison to *yap1*^*kg151*^ single mutant, *yap1*^*kg151*^*;wwtr1*^*kg169/+*^ fish have more severe eye defects including coloboma starting at 2 dpf (arrowheads). Bar = 500 μm. **(B)** Wholemounts of genotypically-identified *yap1*^*kg151*^ mutant larvae reared at 28.5°C until 70% epiboly then at 32.0°C until 3 dpf. Compared to wild type and heterozygote siblings (wt, larva 1) (top), the severity of *yap1*^*kg151*^ phenotype varies, with oedema (larvae 2–6, red arrows), small eye pigment area (larvae 2–6) and bent (larvae 2–4) or curled (larvae 5,6, asterisks) bodies. Bottom: pericardial oedema (left) and eye pigmentation (right, outlined in yellow dots). Bars = 100 μm. **(C)** Penetrance of the small eye pigmentation/oedema/bent tail phenotype in fry from three *yap1*^*kg151/+*^ in-cross experiments (Exp 1–3) reared at different temperatures to the equivalent of the 5 dpf developmental stage, as indicated in schematic (top). Number of larvae on bars. Difference from 25% mutant phenotype: Χ^2^; 20.5°C *p* = 2 x 10^-10^, 28.5°C *p* = 3 x 10^-5^, 32.0°C *p* = 0.1. **(D)** Survival beyond 5 dpf-equivalent of *yap1*^*kg151*^ compared to siblings when raised at 28.5°C until 70% epiboly then 20.5°C until 7 days (5 dpf-equivalent) and 28.5°C thereafter. Data pooled from seven *yap1*^*kg151/+*^ in-crosses totalling 576 viable larvae at 7 days (no deaths occurred before 7 days), of which 423 survived to genotyping at 3 months (73%). **(E)** Poor survival beyond 5 dpf-equivalent of *wwtr1*^*kg169*^ pooled from three separate lays. * *p*-values of Χ^2^ tests performed comparing numbers of genotypes obtained against the numbers expected at 1:2:1. **(F)** Survival from 5 dpf to 3 months from a *yap1*^*kg151/+*^*;wwtr1*^*kg169*/+^ in-cross raised at 28.5°C. Note the lack of surviving *yap1*^*kg151/+*^*;wwtr1*^*kg169*/kg169^ larvae (*p* = 0.008; Χ^2^ on survival versus non-*yap1* mutant siblings). Number of fish analysed is indicated on columns.
(PDF)

S4 Fig Sarcomere organisation appears unaffected in *yap1*^*kg151*^ mutant. Heterozygote *yap1*^*kg151/+*^ carriers were crossed and reared at permissive temperature from 70% epiboly and analysed live (A) or after fixation (B) and are shown in lateral view, dorsal to top, anterior to left. **(A)** Dual heterozygote *yap1*^*kg151/+*^*;Tg(actc1b:mCherryCAAX)*^*pc22/+*^ and *yap1*^*kg151/+*^*; Tg(actc1b:LIFEACT-EGFP)*^*pc21/+*^ crossed and larvae reared to 6 dpf-equivalent had normal structure and average sarcomere length (brackets; 1.94 μm) in mutants and siblings. **(B)** α-actinin at 5 dpf-equivalent in larvae from an in-cross of heterozygote *yap1*^*kg151/+*^ parents. Bar = 5 μm.
(PDF)

S5 Fig *Yap1* mutant movement defects are not due to shorter fish length. **(A)** Swimming velocity in mutants is not correlated with their length. **(B)** Maximum trunk angle shows no correlation with total fish length.
(PDF)

S6 Fig Double mutants have normal myogenesis prior to developmental arrest. **(A)** Time course of development of embryos from a dual heterozygote *yap1*^*kg151/+*^*;wwtr1*^*kg169/+*^ in-cross. Lateral view, dorsal to top, anterior to left. Red brackets highlight reduced height of myotome, at 14ss and further reduction at 16ss. By 21ss, yolk elongation fails. Yellow dots marking somite borders highlight shorter length of somites in double mutants compared to siblings. **(B)** In situ mRNA hybridisation for MRF mRNAs in 16ss double mutant (right) and sibling (left). Dorsal flatmount, anterior to top. **(C)** Confocal stacks of flatmounted 18ss *yap1*^*kg151*^;*wwtr1*^*kg169*^ and sibling embryos stained for fast myosin (F310, red), slow myosin (F59, green), and nuclei (Hoechst 33342, blue). Fractions indicate number of genotyped flatmounts showing the phenotype. **(D)** Lateral view confocal stacks of 24ss *yap1*^*kg151*^*;wwtr1*^*kg169*^ and sibling larvae stained for fast myosin (F310, green). **(E,F)** Volume of myotome 17 at 2 dpf in progeny from a dual heterozygote *yap1*^*kg151/+*^*;wwtr1*^*kg169/+*^ in-cross (E) or a *yap1*^*kg151*^ mutant female crossed with a *yap1*^*kg151/+*^*;wwtr1*^*kg169/+*^ male (F), each grown at the non-permissive temperature. Numbers of fish analysed are indicated on columns. Bars: A = 200 μm; B = 100 μm; C and D = 50 μm.
(PDF)

S7 Fig Adult *yap1*^*kg137/151*^ and *yap1*^*kg137/152*^ mutants are smaller than their siblings. **(A-C)**. Weight, standard length and Fulton’s condition factor (k) of *wwtr1*^*kg169*^ (A), *yap1*^*kg137*^*, yap1*^*kg151*^ and *yap1*^*kg152*^ (B) and *yap1*^*kg151*^ (C). **(D)** Adult *yap1*^*kg137/kg151*^ and *yap1*^*kg137/kg152*^ mutants reared at the permissive 20.5ºC temperature until 5 dpf-equivalent and then at 26.5ºC are smaller than their wt or heterozygote siblings, points plotted with jitter to avoid overlap. Statistically significant results of Kruskal-Wallis tests are shown (B,C).
(PDF)

S8 Fig Apparently normal notochord cells in *yap1*^*kg151*^ mutant. Notochord cells of *yap1*^*kg151*^ mutants grown at 20.5°C until 3 day (equivalent to ~2 dpf) were indistinguishable from those of their wild type siblings. Box indicates location of magnified images, showing variability between individuals unrelated to genotype. Bars = 100 μm.
(PDF)

S9 Fig Zebrafish *yap1*^*kg151*^ mutants lack a morphological ciliary defect in cerebral ventricle. **(A)** Schematic dorsal view (left) of adult zebrafish brain showing the sagittal dissection (red line), and lateral view (right) of the dissected brain with the position of imagining in the rhombencephalic ventricle marked (red box). **(B-D)** Scanning electron micrographs of the internal surface of the rhombencephalic ventricle in sibling wildtype (B) and *yap*^*1kg151*^ mutants without (C) or with (D) spinal curvature phenotype at two magnifications. OB; olfactory bulb, Tel; telencephalon, TeO; optic tectum, CCe; corpus cerebelli, CC; crista cerebralis, V; ventricle. Bars = 10 μm.
(PDF)

S10 Fig Reissner’s fibre is present in *yap1*^*kg151*^ mutant. Immunofluorescent detection of AFRU Reissner’s fibre antigen (green, arrowheads) and nuclei (blue) in the posterior spinal canal at the 5 dpf-equivalent stage in single optical slices (top) or maximum intensity projection in a genotyped *yap*^*kg151*^ mutant. Single channels are shown beneath in grayscale. Note the intense AFRU signal at the posterior tip of the spinal cord (arrows). Bar = 50 μm.
(PDF)

S11 Fig Defective distribution of *col8a1a* mRNA in hypochord of *yap1*^*kg151*^ mutants. In situ mRNA hybridisation for *col8a1a* mRNA in additional sibling 36 hpf embryos to those shown in Fig 6B. **(A)** Mutants show patchy axial distribution in hypochord (arrowheads), but more even signal in notochord (arrows). Hypochord signal is absent for one or more whole somite lengths in mutants. **(B,C)** In contrast, heterozygous (B) and wild type (C) siblings show continuous hypochord signal throughout the axis at higher level than that in notochord. Bar = 100 μm.
(PDF)

S1 Data (XLSX)

S1 Table Staging of fish reared at 28.5ºC until 70% epiboly and at 20.5ºC thereafter. (PDF)

S2 Table Primers used in qRT-PCR and to make probes for in situ mRNA hybridisation. (PDF)

## References

[1] Wise CA, Sepich D, Ushiki A, Khanshour AM, Kidane YH, Makki N, et al. The cartilage matrisome in adolescent idiopathic scoliosis. Bone Res. 2020;8:13. doi: 10.1038/s41413-020-0089-0 32195011 PMC7062733

[2] Faldini C, Manzetti M, Neri S, Barile F, Viroli G, Geraci G, et al. Epigenetic and Genetic Factors Related to Curve Progression in Adolescent Idiopathic Scoliosis: A Systematic Scoping Review of the Current Literature. International Journal of Molecular Sciences. 2022;23(11):5914. doi: 10.3390/ijms23115914 35682604 PMC9180299

[3] Tuncay IO, Lee EK, Gustafson A, Lee Y, Jung D, Koh J-Y, et al. Whole genome sequencing in adolescent idiopathic scoliosis cohort implicates multiple biological pathways. npj Genomic Medicine. 2025;10(1):67. doi: 10.1038/s41525-025-00520-5 41073408 PMC12514243

[4] Kou I, Otomo N, Takeda K, Momozawa Y, Lu H-F, Kubo M, et al. Genome-wide association study identifies 14 previously unreported susceptibility loci for adolescent idiopathic scoliosis in Japanese. Nat Commun. 2019;10(1):3685. doi: 10.1038/s41467-019-11596-w 31417091 PMC6695451

[5] Conway KM, Gedlinske A, Mathews KD, Perlman S, Johnson N, Butterfield R, et al. A population-based study of scoliosis among males diagnosed with a dystrophinopathy identified by the Muscular Dystrophy Surveillance, Tracking, and Research Network (MD STARnet). Muscle Nerve. 2022;65(2):193–202. doi: 10.1002/mus.27464 34787322 PMC8752499

[6] Bharadwaj A, Sharma J, Singh J, Kumari M, Dargar T, Kalita B, et al. Musculoskeletal defects associated with myosin heavy chain-embryonic loss of function are mediated by the YAP signaling pathway. EMBO Mol Med. 2023;15(9):e17187. doi: 10.15252/emmm.202217187 37492882 PMC10493586

[7] Bourgeois A, Esteves de Lima J, Charvet B, Kawakami K, Stricker S, Duprez D. Stable and bicistronic expression of two genes in somite- and lateral plate-derived tissues to study chick limb development. BMC Dev Biol. 2015;15:39. doi: 10.1186/s12861-015-0088-3 26518454 PMC4628273

[8] Dong Y, Yuan H, Ma G, Cao H. Bone-muscle crosstalk under physiological and pathological conditions. Cell Mol Life Sci. 2024;81(1):310. doi: 10.1007/s00018-024-05331-y 39066929 PMC11335237

[9] Gaut L, Duprez D. Tendon development and diseases. Wiley Interdiscip Rev Dev Biol. 2016;5(1):5–23. doi: 10.1002/wdev.201 26256998

[10] Usami Y, Iijima H, Kokubun T. Exploring the role of mechanical forces on tendon development using in vivo model: a scoping review. Dev Dyn. 2024;253(6):550–65. doi: 10.1002/dvdy.673 37947268

[11] Braun T, Rudnicki MA, Arnold HH, Jaenisch R. Targeted inactivation of the muscle regulatory gene Myf-5 results in abnormal rib development and perinatal death. Cell. 1992;71(3):369–82. doi: 10.1016/0092-8674(92)90507-9 1423602

[12] Hinits Y, Williams VC, Sweetman D, Donn TM, Ma TP, Moens CB, et al. Defective cranial skeletal development, larval lethality and haploinsufficiency in Myod mutant zebrafish. Dev Biol. 2011;358(1):102–12. doi: 10.1016/j.ydbio.2011.07.015 21798255 PMC3360969

[13] Ashby PR, Pinçon-Raymond M, Harris AJ. Regulation of myogenesis in paralyzed muscles in the mouse mutants peroneal muscular atrophy and muscular dysgenesis. Dev Biol. 1993;156(2):529–36. doi: 10.1006/dbio.1993.1099 8462749

[14] Hall BK, Herring SW. Paralysis and growth of the musculoskeletal system in the embryonic chick. J Morphol. 1990;206(1):45–56. doi: 10.1002/jmor.1052060105 2246789

[15] Hosseini A, Hogg DA. The effects of paralysis on skeletal development in the chick embryo. I. General effects. J Anat. 1991;177:159–68. 1769890 PMC1260423

[16] Hosseini A, Hogg DA. The effects of paralysis on skeletal development in the chick embryo. II. Effects on histogenesis of the tibia. J Anat. 1991;177:169–78. 1769891 PMC1260424

[17] Karlsson MK, Hasserius R, Obrant KJ. Bone mineral density in athletes during and after career: a comparison between loaded and unloaded skeletal regions. Calcif Tissue Int. 1996;59(4):245–8. doi: 10.1007/s002239900117 8781046

[18] Karlsson MK, Johnell O, Obrant KJ. Bone mineral density in professional ballet dancers. Bone Miner. 1993;21(3):163–9. doi: 10.1016/s0169-6009(08)80227-9 8400916

[19] Kelu JJ, Pipalia TG, Hughes SM. Circadian regulation of muscle growth independent of locomotor activity. Proc Natl Acad Sci U S A. 2020;117(49):31208–18. doi: 10.1073/pnas.2012450117 33229575 PMC7733834

[20] Skerry TM. The response of bone to mechanical loading and disuse: fundamental principles and influences on osteoblast/osteocyte homeostasis. Arch Biochem Biophys. 2008;473(2):117–23. doi: 10.1016/j.abb.2008.02.028 18334226

[21] Subramanian A, Kanzaki LF, Galloway JL, Schilling TF. Mechanical force regulates tendon extracellular matrix organization and tenocyte morphogenesis through TGFbeta signaling. Elife. 2018;7:e38069. doi: 10.7554/eLife.38069 30475205 PMC6345564

[22] Suominen H. Bone Mineral Density and Long Term Exercise. Sports Medicine. 1993;16(5):316–30. doi: 10.2165/00007256-199316050-00003 8272687

[23] Karlsson MK, Johnell O, Obrant KJ. Bone mineral density in weight lifters. Calcif Tissue Int. 1993;52(3):212–5. doi: 10.1007/BF00298721 8481835

[24] Shen H, Grimston S, Civitelli R, Thomopoulos S. Deletion of connexin43 in osteoblasts/osteocytes leads to impaired muscle formation in mice. J Bone Miner Res. 2015;30(4):596–605. doi: 10.1002/jbmr.2389 25348938 PMC4444057

[25] Attwaters M, Hughes SM. Cellular and molecular pathways controlling muscle size in response to exercise. FEBS J. 2022;289(6):1428–56. doi: 10.1111/febs.15820 33755332

[26] Nikander R, Sievänen H, Heinonen A, Kannus P. Femoral neck structure in adult female athletes subjected to different loading modalities. J Bone Miner Res. 2005;20(3):520–8. doi: 10.1359/JBMR.041119 15746998

[27] Roberts MD, McCarthy JJ, Hornberger TA, Phillips SM, Mackey AL, Nader GA, et al. Mechanisms of mechanical overload-induced skeletal muscle hypertrophy: current understanding and future directions. Physiol Rev. 2023;103(4):2679–757. doi: 10.1152/physrev.00039.2022 37382939 PMC10625844

[28] Dirks ML, Wall BT, van de Valk B, Holloway TM, Holloway GP, Chabowski A, et al. One Week of Bed Rest Leads to Substantial Muscle Atrophy and Induces Whole-Body Insulin Resistance in the Absence of Skeletal Muscle Lipid Accumulation. Diabetes. 2016;65(10):2862–75. doi: 10.2337/db15-1661 27358494

[29] Dos Santos VR, de Silva BSA, Agostinete RR, Batista VC, Gobbo LA. Older adults physically inactive in occupational and commuting domains have a higher risk for osteopenia and osteoporosis: A 12-month prospective study. Arch Osteoporos. 2023;18(1):80. doi: 10.1007/s11657-023-01294-y 37280379

[30] Nicks DK, Beneke WM, Key RM, Timson BF. Muscle fibre size and number following immobilisation atrophy. J Anat. 1989;163:1–5. 2558097 PMC1256510

[31] Sánchez-Sánchez JL, He L, Morales JS, de Souto Barreto P, Jiménez-Pavón D, Carbonell-Baeza A, et al. Association of physical behaviours with sarcopenia in older adults: a systematic review and meta-analysis of observational studies. Lancet Healthy Longev. 2024;5(2):e108–19. doi: 10.1016/S2666-7568(23)00241-6 38310891

[32] Schoutens A, Laurent E, Poortmans JR. Effects of inactivity and exercise on bone. Sports Med. 1989;7(2):71–81. doi: 10.2165/00007256-198907020-00001 2646672

[33] Tournadre A, Vial G, Capel F, Soubrier M, Boirie Y. Sarcopenia. Joint Bone Spine. 2019;86:309–14. doi: 10.1016/j.jbspin.2018.08.001 30098424

[34] Hueter C. XXIII. Anatomische Studien an den Extremitätengelenken Neugeborener und Erwachsener. Archiv für pathologische Anatomie und Physiologie und für klinische Medicin Band 26. De Gruyter. 1863. p. 484–519. doi: 10.1515/9783112391280-023

[35] Mente PL, Aronsson DD, Stokes IA, Iatridis JC. Mechanical modulation of growth for the correction of vertebral wedge deformities. J Orthop Res. 1999;17(4):518–24. doi: 10.1002/jor.1100170409 10459757

[36] Roaf R. Vertebral growth and its mechanical control. J Bone Joint Surg Br. 1960;42-B:40–59. doi: 10.1302/0301-620X.42B1.40 13854527

[37] Stokes IAF. Mechanical effects on skeletal growth. J Musculoskelet Neuronal Interact. 2002;2(3):277–80. 15758453

[38] Stokes IA, Spence H, Aronsson DD, Kilmer N. Mechanical modulation of vertebral body growth. Implications for scoliosis progression. Spine (Phila Pa 1976). 1996;21(10):1162–7. doi: 10.1097/00007632-199605150-00007 8727190

[39] von Volkmann R. Veilletzungen und Krankheiten der Bewegungsorgane. Stuttgart: Verlag von Ferdinand Enke. 1882.

[40] Li Z, Lin J, Wu J, Suo J, Wang Z. The Hippo signalling pathway in bone homeostasis: Under the regulation of mechanics and aging. Cell Prolif. 2024;57(10):e13652. doi: 10.1111/cpr.13652 38700015 PMC11471399

[41] Zhang C, Wang F, Gao Z, Zhang P, Gao J, Wu X. Regulation of Hippo Signaling by Mechanical Signals and the Cytoskeleton. DNA Cell Biol. 2020;39(2):159–66. doi: 10.1089/dna.2019.5087 31821009

[42] Han J, Zhang J, Zhang X, Luo W, Liu L, Zhu Y, et al. Emerging role and function of Hippo-YAP/TAZ signaling pathway in musculoskeletal disorders. Stem Cell Res Therapy. 2024;15:386. doi: 10.1186/s13287-024-04011-9 39468616 PMC11520482

[43] Wackerhage H, Del Re DP, Judson RN, Sudol M, Sadoshima J. The Hippo signal transduction network in skeletal and cardiac muscle. Sci Signal. 2014;7(337):re4. doi: 10.1126/scisignal.2005096 25097035

[44] Watt KI, Goodman CA, Hornberger TA, Gregorevic P. The Hippo Signaling Pathway in the Regulation of Skeletal Muscle Mass and Function. Exerc Sport Sci Rev. 2018;46(2):92–6. doi: 10.1249/JES.0000000000000142 29346163 PMC6319272

[45] Zhong Z, Jiao Z, Yu F-X. The Hippo signaling pathway in development and regeneration. Cell Rep. 2024;43(3):113926. doi: 10.1016/j.celrep.2024.113926 38457338

[46] Pocaterra A, Romani P, Dupont S. YAP/TAZ functions and their regulation at a glance. J Cell Sci. 2020;133(2):jcs230425. doi: 10.1242/jcs.230425 31996398

[47] Mosaddad SA, Salari Y, Amookhteh S, Soufdoost RS, Seifalian A, Bonakdar S, et al. Response to Mechanical Cues by Interplay of YAP/TAZ Transcription Factors and Key Mechanical Checkpoints of the Cell: A Comprehensive Review. Cell Physiol Biochem. 2021;55(1):33–60. doi: 10.33594/000000325 33474906

[48] Nakajima H, Yamamoto K, Agarwala S, Terai K, Fukui H, Fukuhara S, et al. Flow-Dependent Endothelial YAP Regulation Contributes to Vessel Maintenance. Dev Cell. 2017;40(6):523-536.e6. doi: 10.1016/j.devcel.2017.02.019 28350986

[49] Vassilev A, Kaneko KJ, Shu H, Zhao Y, DePamphilis ML. TEAD/TEF transcription factors utilize the activation domain of YAP65, a Src/Yes-associated protein localized in the cytoplasm. Genes Dev. 2001;15(10):1229–41. doi: 10.1101/gad.888601 11358867 PMC313800

[50] Dupont S, Morsut L, Aragona M, Enzo E, Giulitti S, Cordenonsi M, et al. Role of YAP/TAZ in mechanotransduction. Nature. 2011;474(7350):179–83. doi: 10.1038/nature10137 21654799

[51] Mason DE, Camacho P, Goeckel ME, Tobin BR, Vega SL, Wu PH, et al. Mechanotransductive feedback control of endothelial cell motility and vascular morphogenesis. eLife. 2025. doi: 10.7554/eLife.86668.3

[52] Stanton AE, Tong X, Lee S, Yang F. Biochemical Ligand Density Regulates Yes-Associated Protein Translocation in Stem Cells through Cytoskeletal Tension and Integrins. ACS Appl Mater Interfaces. 2019;11(9):8849–57. doi: 10.1021/acsami.8b21270 30789697 PMC6881158

[53] Stanton AE, Tong X, Yang F. Extracellular matrix type modulates mechanotransduction of stem cells. Acta Biomater. 2019;96:310–20. doi: 10.1016/j.actbio.2019.06.048 31255664 PMC8735670

[54] Panciera T, Azzolin L, Cordenonsi M, Piccolo S. Mechanobiology of YAP and TAZ in physiology and disease. Nat Rev Mol Cell Biol. 2017;18(12):758–70. doi: 10.1038/nrm.2017.87 28951564 PMC6192510

[55] Hossain Z, Ali SM, Ko HL, Xu J, Ng CP, Guo K, et al. Glomerulocystic kidney disease in mice with a targeted inactivation of Wwtr1. Proc Natl Acad Sci U S A. 2007;104(5):1631–6. doi: 10.1073/pnas.0605266104 17251353 PMC1785239

[56] Kegelman CD, Mason DE, Dawahare JH, Horan DJ, Vigil GD, Howard SS, et al. Skeletal cell YAP and TAZ combinatorially promote bone development. FASEB J. 2018;32(5):2706–21. doi: 10.1096/fj.201700872R 29401582 PMC5901392

[57] Piccolo S, Sladitschek-Martens HL, Cordenonsi M. Mechanosignaling in vertebrate development. Dev Biol. 2022;488:54–67. doi: 10.1016/j.ydbio.2022.05.005 35580730

[58] Sun C, De Mello V, Mohamed A, Ortuste Quiroga HP, Garcia-Munoz A, Al Bloshi A, et al. Common and Distinctive Functions of the Hippo Effectors Taz and Yap in Skeletal Muscle Stem Cell Function. Stem Cells. 2017;35(8):1958–72. doi: 10.1002/stem.2652 28589555 PMC5575518

[59] Vanyai HK, Prin F, Guillermin O, Marzook B, Boeing S, Howson A, et al. Control of skeletal morphogenesis by the Hippo-YAP/TAZ pathway. Development. 2020;147(21):dev187187. doi: 10.1242/dev.187187 32994166 PMC7673359

[60] Xiong J, Almeida M, O’Brien CA. The YAP/TAZ transcriptional co-activators have opposing effects at different stages of osteoblast differentiation. Bone. 2018;112:1–9. doi: 10.1016/j.bone.2018.04.001 29626544 PMC5970058

[61] Mar JH, Ordahl CP. A conserved CATTCCT motif is required for skeletal muscle-specific activity of the cardiac troponin T gene promoter. Proc Natl Acad Sci U S A. 1988;85(17):6404–8. doi: 10.1073/pnas.85.17.6404 3413104 PMC281980

[62] Yoshida T. MCAT elements and the TEF-1 family of transcription factors in muscle development and disease. Arterioscler Thromb Vasc Biol. 2008;28(1):8–17. doi: 10.1161/ATVBAHA.107.155788 17962623

[63] Qin S, Li C, Lu H, Feng Y, Guo T, Han Y, et al. Biology of Hippo signaling pathway: Skeletal muscle development and beyond. J Integr Agric. 2024;23:1825–38. doi: 10.1016/j.jia.2023.09.031 33259860

[64] Kaya-Çopur A, Marchiano F, Hein MY, Alpern D, Russeil J, Luis NM, et al. The Hippo pathway controls myofibril assembly and muscle fiber growth by regulating sarcomeric gene expression. Elife. 2021;10:e63726. doi: 10.7554/eLife.63726 33404503 PMC7815313

[65] Weitkunat M, Kaya-Çopur A, Grill SW, Schnorrer F. Tension and force-resistant attachment are essential for myofibrillogenesis in Drosophila flight muscle. Curr Biol. 2014;24(7):705–16. doi: 10.1016/j.cub.2014.02.032 24631244

[66] Morin-Kensicki EM, Boone BN, Howell M, Stonebraker JR, Teed J, Alb JG, et al. Defects in yolk sac vasculogenesis, chorioallantoic fusion, and embryonic axis elongation in mice with targeted disruption of Yap65. Mol Cell Biol. 2006;26(1):77–87. doi: 10.1128/MCB.26.1.77-87.2006 16354681 PMC1317614

[67] Judson RN, Tremblay AM, Knopp P, White RB, Urcia R, De Bari C, et al. The Hippo pathway member Yap plays a key role in influencing fate decisions in muscle satellite cells. J Cell Sci. 2012;125(Pt 24):6009–19. doi: 10.1242/jcs.109546 23038772 PMC3585517

[68] Judson RN, Gray SR, Walker C, Carroll AM, Itzstein C, Lionikas A, et al. Constitutive expression of Yes-associated protein (Yap) in adult skeletal muscle fibres induces muscle atrophy and myopathy. PLoS One. 2013;8(3):e59622. doi: 10.1371/journal.pone.0059622 23544078 PMC3609830

[69] Goodman CA, Dietz JM, Jacobs BL, McNally RM, You J-S, Hornberger TA. Yes-Associated Protein is up-regulated by mechanical overload and is sufficient to induce skeletal muscle hypertrophy. FEBS Lett. 2015;589(13):1491–7. doi: 10.1016/j.febslet.2015.04.047 25959868 PMC4442043

[70] Kaneshige A, Kaji T, Zhang L, Saito H, Nakamura A, Kurosawa T, et al. Relayed signaling between mesenchymal progenitors and muscle stem cells ensures adaptive stem cell response to increased mechanical load. Cell Stem Cell. 2022;29(2):265-280.e6. doi: 10.1016/j.stem.2021.11.003 34856120

[71] Makita R, Uchijima Y, Nishiyama K, Amano T, Chen Q, Takeuchi T, et al. Multiple renal cysts, urinary concentration defects, and pulmonary emphysematous changes in mice lacking TAZ. Am J Physiol Renal Physiol. 2008;294(3):F542-53. doi: 10.1152/ajprenal.00201.2007 18172001

[72] Tian Y, Kolb R, Hong J-H, Carroll J, Li D, You J, et al. TAZ promotes PC2 degradation through a SCFbeta-Trcp E3 ligase complex. Mol Cell Biol. 2007;27(18):6383–95. doi: 10.1128/MCB.00254-07 17636028 PMC2099608

[73] Gessler L, Huraskin D, Jian Y, Eiber N, Hu Z, Prószyński TJ, et al. The YAP1/TAZ-TEAD transcriptional network regulates gene expression at neuromuscular junctions in skeletal muscle fibers. Nucleic Acids Res. 2024;52(2):600–24. doi: 10.1093/nar/gkad1124 38048326 PMC10810223

[74] Hong J-H, Hwang ES, McManus MT, Amsterdam A, Tian Y, Kalmukova R, et al. TAZ, a transcriptional modulator of mesenchymal stem cell differentiation. Science. 2005;309(5737):1074–8. doi: 10.1126/science.1110955 16099986

[75] Deng Y, Wu A, Li P, Li G, Qin L, Song H, et al. Yap1 Regulates Multiple Steps of Chondrocyte Differentiation during Skeletal Development and Bone Repair. Cell Rep. 2016;14(9):2224–37. doi: 10.1016/j.celrep.2016.02.021 26923596

[76] Li Y, Yang S, Qin L, Yang S. TAZ is required for chondrogenesis and skeletal development. Cell Discov. 2021;7(1):26. doi: 10.1038/s41421-021-00254-5 33879790 PMC8058044

[77] Zhao L, Guan H, Song C, Wang Y, Liu C, Cai C, et al. YAP1 is essential for osteoclastogenesis through a TEADs-dependent mechanism. Bone. 2018;110:177–86. doi: 10.1016/j.bone.2018.01.035 29432919

[78] Xiao Z, Cao L, Smith MD, Li H, Li W, Smith JC, et al. Genetic interactions between polycystin-1 and Wwtr1 in osteoblasts define a novel mechanosensing mechanism regulating bone formation in mice. Bone Res. 2023;11(1):57. doi: 10.1038/s41413-023-00295-4 37884491 PMC10603112

[79] Kimelman D, Smith NL, Lai JKH, Stainier DY. Regulation of posterior body and epidermal morphogenesis in zebrafish by localized Yap1 and Wwtr1. Elife. 2017;6:e31065. doi: 10.7554/eLife.31065 29283341 PMC5773182

[80] Groves JA, Hammond CL, Hughes SM. Fgf8 drives myogenic progression of a novel lateral fast muscle fibre population in zebrafish. Development. 2005;132(19):4211–22. doi: 10.1242/dev.01958 16120642

[81] Ganassi M, Badodi S, Ortuste Quiroga HP, Zammit PS, Hinits Y, Hughes SM. Myogenin promotes myocyte fusion to balance fibre number and size. Nat Commun. 2018;9(1):4232. doi: 10.1038/s41467-018-06583-6 30315160 PMC6185967

[82] Ganassi M, Badodi S, Wanders K, Zammit PS, Hughes SM. Myogenin is an essential regulator of adult myofibre growth and muscle stem cell homeostasis. Elife. 2020;9:e60445. doi: 10.7554/eLife.60445 33001028 PMC7599067

[83] Hinits Y, Osborn DPS, Hughes SM. Differential requirements for myogenic regulatory factors distinguish medial and lateral somitic, cranial and fin muscle fibre populations. Development. 2009;136(3):403–14. doi: 10.1242/dev.028019 19141670 PMC2687589

[84] Roy SD, Williams VC, Pipalia TG, Li K, Hammond CL, Knappe S, et al. Myotome adaptability confers developmental robustness to somitic myogenesis in response to fibre number alteration. Dev Biol. 2017;431(2):321–35. doi: 10.1016/j.ydbio.2017.08.029 28887016 PMC5667637

[85] Attwaters M, Kelu JJ, Pipalia TG, Hughes SM. Real Time and Repeated Measurement of Skeletal Muscle Growth in Individual Live Zebrafish Subjected to Altered Electrical Activity. J Vis Exp. 2022;(184):10.3791/64063. doi: 10.3791/64063 35781279

[86] Miesfeld JB, Gestri G, Clark BS, Flinn MA, Poole RJ, Bader JR, et al. Yap and Taz regulate retinal pigment epithelial cell fate. Development. 2015;142(17):3021–32. doi: 10.1242/dev.119008 26209646 PMC4582179

[87] Xu D, Lv J, He L, Fu L, Hu R, Cao Y, et al. Scribble influences cyst formation in autosomal-dominant polycystic kidney disease by regulating Hippo signaling pathway. FASEB J. 2018;32(8):4394–407. doi: 10.1096/fj.201701376RR 29529391

[88] Dingare C, Niedzwetzki A, Klemmt PA, Godbersen S, Fuentes R, Mullins MC, et al. The Hippo pathway effector Taz is required for cell morphogenesis and fertilization in zebrafish. Development. 2018;145(22):dev167023. doi: 10.1242/dev.167023 30327325 PMC6262795

[89] Ricker WE. Computation and interpretation of biological statistics of fish populations. Fish Res Board Can Bull. 1975;191:1–382. https://waves-vagues.dfo-mpo.gc.ca/Library/1485.pdf

[90] Schreiber AM. Asymmetric craniofacial remodeling and lateralized behavior in larval flatfish. J Exp Biol. 2006;209(Pt 4):610–21. doi: 10.1242/jeb.02056 16449556

[91] Bagwell J, Norman J, Ellis K, Peskin B, Hwang J, Ge X, et al. Notochord vacuoles absorb compressive bone growth during zebrafish spine formation. Elife. 2020;9:e51221. doi: 10.7554/eLife.51221 31995030 PMC7012607

[92] Christiansen HE, Lang MR, Pace JM, Parichy DM. Critical early roles for col27a1a and col27a1b in zebrafish notochord morphogenesis, vertebral mineralization and post-embryonic axial growth. PLoS One. 2009;4(12):e8481. doi: 10.1371/journal.pone.0008481 20041163 PMC2794549

[93] Wang M, Zhao S, Shi C, Guyot M-C, Liao M, Tauer JT, et al. Planar cell polarity zebrafish models of congenital scoliosis reveal underlying defects in notochord morphogenesis. Development. 2024;151(21):dev202829. doi: 10.1242/dev.202829 39417583 PMC11698040

[94] Wopat S, Adhyapok P, Daga B, Crawford JM, Norman J, Bagwell J, et al. Notochord segmentation in zebrafish controlled by iterative mechanical signaling. Dev Cell. 2024;59(14):1860-1875.e5. doi: 10.1016/j.devcel.2024.04.013 38697108 PMC11265980

[95] Park R, Moon UY, Park JY, Hughes LJ, Johnson RL, Cho S-H, et al. Yap is required for ependymal integrity and is suppressed in LPA-induced hydrocephalus. Nat Commun. 2016;7:10329. doi: 10.1038/ncomms10329 26754915 PMC4729961

[96] Grimes DT, Boswell CW, Morante NFC, Henkelman RM, Burdine RD, Ciruna B. Zebrafish models of idiopathic scoliosis link cerebrospinal fluid flow defects to spine curvature. Science. 2016;352(6291):1341–4. doi: 10.1126/science.aaf6419 27284198 PMC5574193

[97] Xie H, Li M, Kang Y, Zhang J, Zhao C. Zebrafish: an important model for understanding scoliosis. Cell Mol Life Sci. 2022;79(9):506. doi: 10.1007/s00018-022-04534-5 36059018 PMC9441191

[98] Rose CD, Pompili D, Henke K, Van Gennip JLM, Meyer-Miner A, Rana R, et al. SCO-Spondin Defects and Neuroinflammation Are Conserved Mechanisms Driving Spinal Deformity across Genetic Models of Idiopathic Scoliosis. Curr Biol. 2020;30(12):2363-2373.e6. doi: 10.1016/j.cub.2020.04.020 32386528

[99] Troutwine BR, Gontarz P, Konjikusic MJ, Minowa R, Monstad-Rios A, Sepich DS, et al. The Reissner fiber is highly dynamic in vivo and controls morphogenesis of the spine. Curr. Biol. 2020;30:2353–62.e2353. doi: 10.1016/j.cub.2020.04.015 32386529 PMC7891109

[100] Sun X, Zhou Y, Zhang R, Wang Z, Xu M, Zhang D, et al. Dstyk mutation leads to congenital scoliosis-like vertebral malformations in zebrafish via dysregulated mTORC1/TFEB pathway. Nat Commun. 2020;11(1):479. doi: 10.1038/s41467-019-14169-z 31980602 PMC6981171

[101] Froese R. Cube law, condition factor and weight–length relationships: history, meta‐analysis and recommendations. J App Ichthyol. 2006;22:241–53. doi: 10.1111/j.1439-0426.2006.00805.x

[102] Fulton TW. The rate of growth of fishes. Fisheries Board of Scotland. 1904.

[103] Dooley CM, Wali N, Sealy IM, White RJ, Stemple DL, Collins JE, et al. The gene regulatory basis of genetic compensation during neural crest induction. PLoS Genet. 2019;15(6):e1008213. doi: 10.1371/journal.pgen.1008213 31199790 PMC6594659

[104] Astone M, Lai JKH, Dupont S, Stainier DYR, Argenton F, Vettori A. Zebrafish mutants and TEAD reporters reveal essential functions for Yap and Taz in posterior cardinal vein development. Sci Rep. 2018;8(1):10189. doi: 10.1038/s41598-018-27657-x 29976931 PMC6033906

[105] Fillatre J, Fauny J-D, Fels JA, Li C, Goll M, Thisse C, et al. TEADs, Yap, Taz, Vgll4s transcription factors control the establishment of Left-Right asymmetry in zebrafish. Elife. 2019;8:e45241. doi: 10.7554/eLife.45241 31513014 PMC6759317

[106] Yi X, Yu J, Ma C, Li L, Luo L, Li H, et al. Yap1/Taz are essential for the liver development in zebrafish. Biochem Biophys Res Commun. 2018;503(1):131–7. doi: 10.1016/j.bbrc.2018.05.196 29859190

[107] Burd CG, Mustol PA, Schu PV, Emr SD. A yeast protein related to a mammalian Ras-binding protein, Vps9p, is required for localization of vacuolar proteins. Mol Cell Biol. 1996;16(5):2369–77. doi: 10.1128/MCB.16.5.2369 8628304 PMC231225

[108] Onogi T, Yamazoe M, Ichinose C, Niki H, Hiraga S. Null mutation of the dam or seqA gene suppresses temperature-sensitive lethality but not hypersensitivity to novobiocin of muk null mutants. J Bacteriol. 2000;182(20):5898–901. doi: 10.1128/JB.182.20.5898-5901.2000 11004192 PMC94715

[109] Wente SR, Blobel G. A temperature-sensitive NUP116 null mutant forms a nuclear envelope seal over the yeast nuclear pore complex thereby blocking nucleocytoplasmic traffic. J Cell Biol. 1993;123(2):275–84. doi: 10.1083/jcb.123.2.275 7691829 PMC2119834

[110] McMurray M. Lean forward: Genetic analysis of temperature-sensitive mutants unfolds the secrets of oligomeric protein complex assembly. Bioessays. 2014;36(9):836–46. doi: 10.1002/bies.201400062 25048147 PMC4229136

[111] Bornhorst D, Xia P, Nakajima H, Dingare C, Herzog W, Lecaudey V, et al. Biomechanical signaling within the developing zebrafish heart attunes endocardial growth to myocardial chamber dimensions. Nat Commun. 2019;10(1):4113. doi: 10.1038/s41467-019-12068-x 31511517 PMC6739419

[112] Duchemin A-L, Vignes H, Vermot J. Mechanically activated piezo channels modulate outflow tract valve development through the Yap1 and Klf2-Notch signaling axis. Elife. 2019;8:e44706. doi: 10.7554/eLife.44706 31524599 PMC6779468

[113] Flinn MA, Jeffery BE, O’Meara CC, Link BA. Yap is required for scar formation but not myocyte proliferation during heart regeneration in zebrafish. Cardiovasc Res. 2019;115(3):570–7. doi: 10.1093/cvr/cvy243 30295714

[114] Fukui H, Terai K, Nakajima H, Chiba A, Fukuhara S, Mochizuki N. S1P-Yap1 signaling regulates endoderm formation required for cardiac precursor cell migration in zebrafish. Dev Cell. 2014;31(1):128–36. doi: 10.1016/j.devcel.2014.08.014 25313964

[115] Miesfeld JB, Link BA. Establishment of transgenic lines to monitor and manipulate Yap/Taz-Tead activity in zebrafish reveals both evolutionarily conserved and divergent functions of the Hippo pathway. Mech Dev. 2014;133:177–88. doi: 10.1016/j.mod.2014.02.003 24560909 PMC4138299

[116] Elmonem MA, Khalil R, Khodaparast L, Khodaparast L, Arcolino FO, Morgan J, et al. Cystinosis (ctns) zebrafish mutant shows pronephric glomerular and tubular dysfunction. Sci Rep. 2017;7:42583. doi: 10.1038/srep42583 28198397 PMC5309805

[117] Hanke N, Staggs L, Schroder P, Litteral J, Fleig S, Kaufeld J, et al. “Zebrafishing” for novel genes relevant to the glomerular filtration barrier. Biomed Res Int. 2013;2013:658270. doi: 10.1155/2013/658270 24106712 PMC3784067

[118] Molina L, Nejak-Bowen K, Monga SP. Role of YAP1 signaling in biliary development, repair, and disease. Seminars in Liver Disease. 2022;:017–33. doi: 10.1055/s-0041-1742277 35073587 PMC9372714

[119] Oh S-H, Swiderska-Syn M, Jewell ML, Premont RT, Diehl AM. Liver regeneration requires Yap1-TGFβ-dependent epithelial-mesenchymal transition in hepatocytes. J Hepatol. 2018;69(2):359–67. doi: 10.1016/j.jhep.2018.05.008 29758331 PMC6349217

[120] Ren Z, Zhang Z, Liu T-M, Ge W. Novel zebrafish polycystic kidney disease models reveal functions of the Hippo pathway in renal cystogenesis. Dis Model Mech. 2021;14(11):dmm049027. doi: 10.1242/dmm.049027 34545930 PMC8592019

[121] Le Guen L, Karpanen T, Schulte D, Harris NC, Koltowska K, Roukens G, et al. Ccbe1 regulates Vegfc-mediated induction of Vegfr3 signaling during embryonic lymphangiogenesis. Development. 2014;141(6):1239–49. doi: 10.1242/dev.100495 24523457

[122] Yiqin W, Ruimeng Y, Peihong W, Xiaohui L, Hao Y, Hongli H, et al. Tead1a Initiates Transcriptional Priming Through the TEAD1a/YAP-Notch1-Spi1/Cebpα Axis to Promote Neutrophil Fate. Adv Sci (Weinh). 2025;12(41):e05441. doi: 10.1002/advs.202505441 40874937 PMC12591127

[123] Halder G, Dupont S, Piccolo S. Transduction of mechanical and cytoskeletal cues by YAP and TAZ. Nat Rev Mol Cell Biol. 2012;13(9):591–600. doi: 10.1038/nrm3416 22895435

[124] Wada K-I, Itoga K, Okano T, Yonemura S, Sasaki H. Hippo pathway regulation by cell morphology and stress fibers. Development. 2011;138(18):3907–14. doi: 10.1242/dev.070987 21831922

[125] Lanyon LE, Rubin CT. Static vs dynamic loads as an influence on bone remodelling. J Biomech. 1984;17(12):897–905. doi: 10.1016/0021-9290(84)90003-4 6520138

[126] Singleman C, Holtzman NG. Growth and maturation in the zebrafish, Danio rerio: a staging tool for teaching and research. Zebrafish. 2014;11(4):396–406. doi: 10.1089/zeb.2014.0976 24979389 PMC4108942

[127] Bornhorst D, Abdelilah-Seyfried S. Strong as a Hippo’s Heart: Biomechanical Hippo Signaling During Zebrafish Cardiac Development. Front Cell Dev Biol. 2021;9:731101. doi: 10.3389/fcell.2021.731101 34422841 PMC8375320

[128] Lai JKH, Collins MM, Uribe V, Jiménez-Amilburu V, Günther S, Maischein H-M, et al. The Hippo pathway effector Wwtr1 regulates cardiac wall maturation in zebrafish. Development. 2018;145(10):dev159210. doi: 10.1242/dev.159210 29773645

[129] Pappalardo A, Porreca I, Caputi L, De Felice E, Schulte-Merker S, Zannini M, et al. Thyroid development in zebrafish lacking Taz. Mech Dev. 2015;138 Pt 3:268–78. doi: 10.1016/j.mod.2015.10.002 26478012

[130] Wang M, Zhao S, Shi C, Guyot M-C, Liao M, Tauer JT, et al. Planar cell polarity zebrafish models of congenital scoliosis reveal underlying defects in notochord morphogenesis. Development. 2024;151(21):dev202829. doi: 10.1242/dev.202829 39417583 PMC11698040

[131] Attili S, Hughes SM. Anaesthetic tricaine acts preferentially on neural voltage-gated sodium channels and fails to block directly evoked muscle contraction. PLoS One. 2014;9(8):e103751. doi: 10.1371/journal.pone.0103751 25090007 PMC4121177

[132] Fischer M, Rikeit P, Knaus P, Coirault C. YAP-Mediated Mechanotransduction in Skeletal Muscle. Front Physiol. 2016;7:41. doi: 10.3389/fphys.2016.00041 26909043 PMC4754448

[133] Watt KI, Turner BJ, Hagg A, Zhang X, Davey JR, Qian H, et al. The Hippo pathway effector YAP is a critical regulator of skeletal muscle fibre size. Nat Commun. 2015;6:6048. doi: 10.1038/ncomms7048 25581281

[134] Li Y, Xiao C, Li R, Zhong W, Xu G, Zhang W. Role of Yes-associated protein (YAP) in regulation of mesenchymal stem cell tenogenic differentiation. J Mol Histol. 2022;53(2):273–83. doi: 10.1007/s10735-022-10059-9 35048214

[135] Lu J, Yang X, He C, Chen Y, Li C, Li S, et al. Rejuvenation of tendon stem/progenitor cells for functional tendon regeneration through platelet-derived exosomes loaded with recombinant Yap1. Acta Biomater. 2023;161:80–99. doi: 10.1016/j.actbio.2023.02.018 36804538

[136] Tao S-C, Huang J-Y, Li Z-X, Zhan S, Guo S-C. Small extracellular vesicles with LncRNA H19 “overload”: YAP Regulation as a Tendon Repair Therapeutic Tactic. iScience. 2021;24(3):102200. doi: 10.1016/j.isci.2021.102200 33733065 PMC7937563

[137] Cameron-Christie SR, Wells CF, Simon M, Wessels M, Tang CZN, Wei W, et al. Recessive Spondylocarpotarsal Synostosis Syndrome Due to Compound Heterozygosity for Variants in MYH3. Am J Hum Genet. 2018;102(6):1115–25. doi: 10.1016/j.ajhg.2018.04.008 29805041 PMC5992117

[138] Lim PJ, Lindert U, Opitz L, Hausser I, Rohrbach M, Giunta C. Transcriptome Profiling of Primary Skin Fibroblasts Reveal Distinct Molecular Features Between PLOD1- and FKBP14-Kyphoscoliotic Ehlers-Danlos Syndrome. Genes (Basel). 2019;10(7):517. doi: 10.3390/genes10070517 31288483 PMC6678841

[139] Khanshour AM, Kou I, Fan Y, Einarsdottir E, Makki N, Kidane YH, et al. Genome-wide meta-analysis and replication studies in multiple ethnicities identify novel adolescent idiopathic scoliosis susceptibility loci. Hum Mol Genet. 2018;27(22):3986–98. doi: 10.1093/hmg/ddy306 30395268 PMC6488972

[140] Bobowski-Gerard M, Boulet C, Zummo FP, Dubois-Chevalier J, Gheeraert C, Bou Saleh M, et al. Functional genomics uncovers the transcription factor BNC2 as required for myofibroblastic activation in fibrosis. Nat Commun. 2022;13(1):5324. doi: 10.1038/s41467-022-33063-9 36088459 PMC9464213

[141] Draper BW, McCallum CM, Stout JL, Slade AJ, Moens CB. A high-throughput method for identifying N-ethyl-N-nitrosourea (ENU)-induced point mutations in zebrafish. Methods Cell Biol. 2004;77:91–112. doi: 10.1016/s0091-679x(04)77005-3 15602907

[142] Cooper MS, Szeto DP, Sommers-Herivel G, Topczewski J, Solnica-Krezel L, Kang H-C, et al. Visualizing morphogenesis in transgenic zebrafish embryos using BODIPY TR methyl ester dye as a vital counterstain for GFP. Dev Dyn. 2005;232(2):359–68. doi: 10.1002/dvdy.20252 15614774

[143] Berger J, Tarakci H, Berger S, Li M, Hall TE, Arner A, et al. Loss of Tropomodulin4 in the zebrafish mutant träge causes cytoplasmic rod formation and muscle weakness reminiscent of nemaline myopathy. Dis Model Mech. 2014;7(12):1407–15. doi: 10.1242/dmm.017376 25288681 PMC4257009

[144] Westerfield, M, 2007. The Zebrafish Book; A guide for the laboratory use of zebrafish (Danio rerio). 5th edition. Univ. Oregon Press. https://zfin.org/zf_info/zfbook/zfbk.html

[145] Urushibata H, Sasaki K, Takahashi E, Hanada T, Fujimoto T, Arai K, et al. Control of Developmental Speed in Zebrafish Embryos Using Different Incubation Temperatures. Zebrafish. 2021;18(5):316–25. doi: 10.1089/zeb.2021.0022 34491109

[146] Miller JC, Tan S, Qiao G, Barlow KA, Wang J, Xia DF, et al. A TALE nuclease architecture for efficient genome editing. Nat Biotechnol. 2011;29(2):143–8. doi: 10.1038/nbt.1755 21179091

[147] Sander JD, Cade L, Khayter C, Reyon D, Peterson RT, Joung JK, et al. Targeted gene disruption in somatic zebrafish cells using engineered TALENs. Nat Biotechnol. 2011;29(8):697–8. doi: 10.1038/nbt.1934 21822241 PMC3154023

[148] Kelu JJ, Hughes SM. Muscle peripheral circadian clock drives nocturnal protein degradation via raised Ror/Rev-erb balance and prevents premature sarcopenia. Proc Natl Acad Sci U S A. 2025;122(19):e2422446122. doi: 10.1073/pnas.2422446122 40324095 PMC12088385

[149] Hinits Y, Hughes SM. Mef2s are required for thick filament formation in nascent muscle fibres. Development. 2007;134(13):2511–9. doi: 10.1242/dev.007088 17537787 PMC3016612

[150] Hinits Y, Pan L, Walker C, Dowd J, Moens CB, Hughes SM. Zebrafish Mef2ca and Mef2cb are essential for both first and second heart field cardiomyocyte differentiation. Dev Biol. 2012;369(2):199–210. doi: 10.1016/j.ydbio.2012.06.019 22750409 PMC3927553

[151] Walker MB, Kimmel CB. A two-color acid-free cartilage and bone stain for zebrafish larvae. Biotech Histochem. 2007;82(1):23–8. doi: 10.1080/10520290701333558 17510811

[152] Du SJ, Frenkel V, Kindschi G, Zohar Y. Visualizing normal and defective bone development in zebrafish embryos using the fluorescent chromophore calcein. Dev Biol. 2001;238(2):239–46. doi: 10.1006/dbio.2001.0390 11784007

[153] Hau H-TA, Kelu JJ, Ochala J, Hughes SM. Slow myosin heavy chain 1 is required for slow myofibril and muscle fibre growth but not for myofibril initiation. Dev Biol. 2023;499:47–58. doi: 10.1016/j.ydbio.2023.04.002 37121308 PMC10713478

