# Supplementary material for: Yap1 regulates motility and vertebral development and prevents kyphoscoliosis in zebrafish
Source: PLoS Genet. 2026 May 28;22(5):e1012172. doi: 10.1371/journal.pgen.1012172 (PMC13349305; doi:10.1371/journal.pgen.1012172)
Supplement: S1 Fig — In situ mRNA hybridisation for yap1 mRNA (A,C,E,G) and wwtr1 mRNA (B,D,F,H) in genotyped mutant and wild type (wt) sibling embryos from single lays from heterozygote in-crosses of MRF mutants myod1fh261, myogkg125, myf5hu2022 and myf6kg126. Lateral whole mounts, anterior to left, dorsal to top are magnified at right. At lower right, transverse cryosections in the yolk-extension region have dorsal to top and show somitic muscle tissue (yellow dots). (A) Yap1 mRNA is up-regulated in superficial somitic regions (arrowheads) of myod1fh261 mutants (myod1fh261 vs wt sib p = 0.01, myod1fh261 vs myod1fh261/+ p = 0.012, wt sib vs myod1fh261/+ p = 0.877, Kruskal-Wallis, adjusted with Bonferroni-Holm). (B) Myodfh261 mutants have similar intensity but lesser extent of somitic wwtr1 mRNA signal in comparison to wt siblings. (C-H) Yap1 mRNA in myogkg125, myf5hu2022 and myf6kg126 mutants appears comparable to wt siblings (C,E,G). Wwtr1 mRNA accumulated less in myogkg125 mutant somites in comparison to wt sibling (D). Wwtr1 mRNA in myf5hu2022 and myf6kg126 was indistinguishable from wt (F,H). Fractions represent number of embryos with phenotype shown/numbers genotyped, in all cases heterozygotes appeared wt. Bars = 100 μm. (PDF) [file pgen.1012172.s001.pdf]

S1 Fig

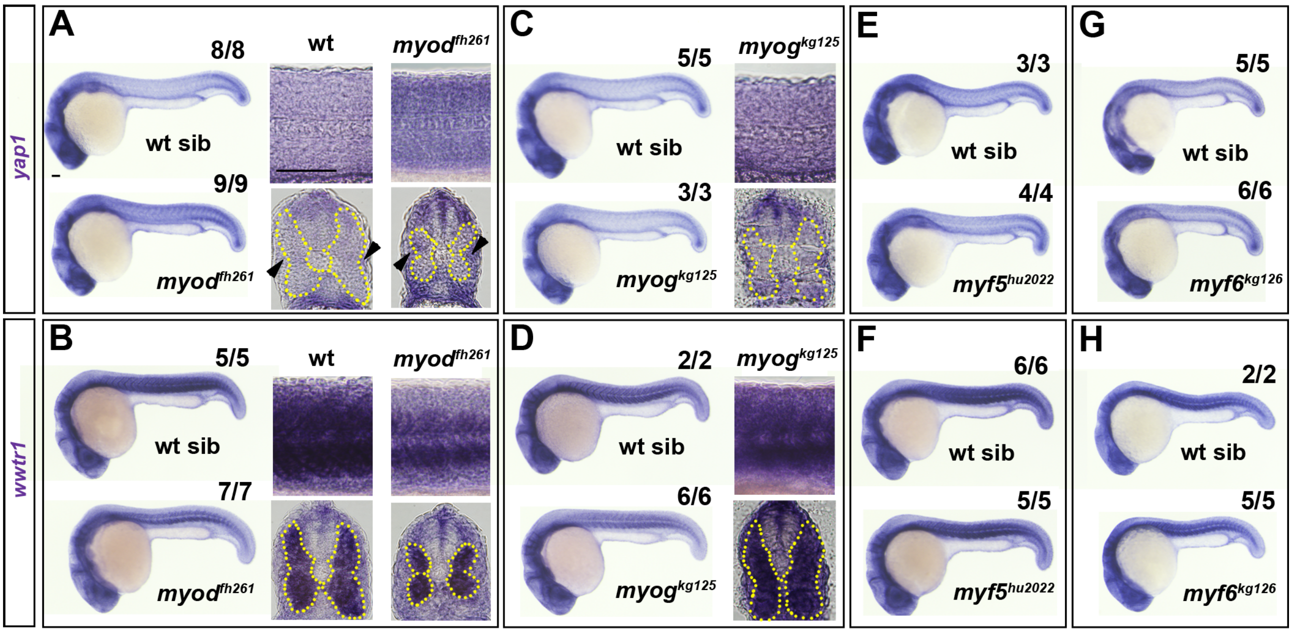

S1 Fig. *Yap1* and *wwtr1* mRNA accumulation is altered in *myod1* mutants.

In situ mRNA hybridisation for *yap1* mRNA (A,C,E,G) and *wwtr1* mRNA (B,D,F,H) in genotyped mutant and wild type (wt) sibling embryos from single lays from heterozygote in-crosses of MRF mutants *myod1*^{fh261}, *myog*^{kg125}, *myf5*^{hu2022} and *myf6*^{kg126}. Lateral whole mounts, anterior to left, dorsal to top are magnified at right. At lower right, transverse cryosections in the yolk-extension region have dorsal to top and show somitic muscle tissue (yellow dots). **(A)** *Yap1* mRNA is up-regulated in superficial somitic regions (arrowheads) of *myod1*^{fh261} mutants (*myod1*^{fh261} vs wt sib $p = 0.01$, *myod1*^{fh261} vs *myod1*$^{fh261/+}$ $p = 0.012$, wt sib vs *myod1*$^{fh261/+}$ $p = 0.877$, Kruskal-Wallis, adjusted with Bonferroni-Holm). **(B)** *Myod*^{fh261} mutants have similar intensity but lesser extent of somitic *wwtr1* mRNA signal in comparison to wt siblings. **(C-H)** *Yap1* mRNA in *myog*^{kg125}, *myf5*^{hu2022} and *myf6*^{kg126} mutants appears comparable to wt siblings (C,E,G). *Wwtr1* mRNA accumulated less in *myog*^{kg125} mutant somites in comparison to wt sibling (D). *Wwtr1* mRNA in *myf5*^{hu2022} and *myf6*^{kg126} was indistinguishable from wt (F,H). Fractions represent number of embryos with phenotype shown/numbers genotyped, in all cases heterozygotes appeared wt. Bars = 100 μm.
