# Supplementary material for: Yap1 regulates motility and vertebral development and prevents kyphoscoliosis in zebrafish
Source: PLoS Genet. 2026 May 28;22(5):e1012172. doi: 10.1371/journal.pgen.1012172 (PMC13349305; doi:10.1371/journal.pgen.1012172)
Supplement: S2 Fig — (A,B) Schematics of yap1 (A) and wwtr1 (B) protein, mRNA and gene showing location of TALEN binding sites in exon 1 and primers used to identify and genotype mutants. (C-G) Mutant alleles showing schematic of protein truncation (top left, red indicates length of predicted nonsense C-terminal peptide), sequencing traces of heterozygote (top right) and in situ mRNA hybridisation showing nonsense-mediated decay in yap1kg151 (C), yap1kg152 (D) and wwtr1kg169 (E), but not yap1kg137 (F) or wwtr1kg133 (G). Fractions show number of each genotype among unsorted embryos and how blind sorting of the three ISH phenotypes was confirmed with 100% genotype accuracy in kg151, kg152 and kg169, but genotypes could not be distinguished by ISH in kg137 or kg133. Bars = 100 μm. (H) qRT-PCR for yap1 or wwtr1 mRNA in wwtr1kg169 and yap1kg151, respectively, in 5 dpf-equivalent larval trunk/tail (left and centre) or adult myotomal muscle (right). Symbol shapes indicate siblings. Mean ± SEM, t-test statistics. (PDF) [file pgen.1012172.s002.pdf]

S2 Fig

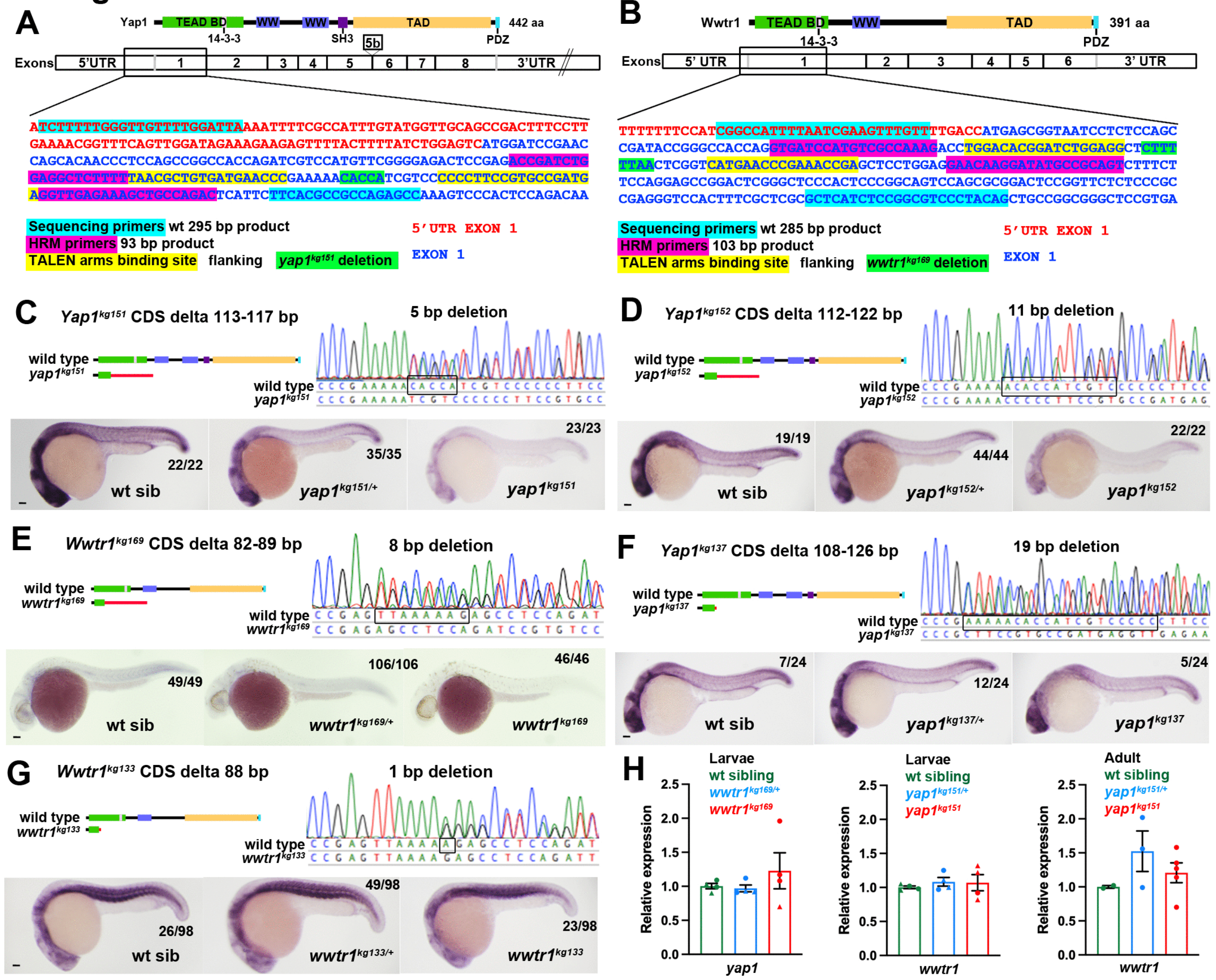

S2 Fig. Genome edited *yap1* and *wwtr1* loss-of-function mutants.

(A,B) Schematics of *yap1* (A) and *wwtr1* (B) protein, mRNA and gene showing location of TALEN binding sites in exon 1 and primers used to identify and genotype mutants. **(C-G)** Mutant alleles showing schematic of protein truncation (top left, red indicates length of predicted nonsense C-terminal peptide), sequencing traces of heterozygote (top right) and in situ mRNA hybridisation showing nonsense-mediated decay in *yap1*^{kg151} (C), *yap1*^{kg152} (D) and *wwtr1*^{kg169} (E), but not *yap1*^{kg137} (F) or *wwtr1*^{kg133} (G). Fractions show number of each genotype among unsorted embryos and how blind sorting of the three ISH phenotypes was confirmed with 100% genotype accuracy in *kg151*, *kg152* and *kg169*, but genotypes could not be distinguished by ISH in *kg137* or *kg133*. Bars = 100 μm. **(H)** qRT-PCR for *yap1* or *wwtr1* mRNA in *wwtr1*^{kg169} and *yap1*^{kg151}, respectively, in 5 dpf-equivalent larval trunk/tail (left and centre) or adult myotomal muscle (right). Symbol shapes indicate siblings. Mean ± SEM, t-test statistics.
