# Supplementary material for: Yap1 regulates motility and vertebral development and prevents kyphoscoliosis in zebrafish
Source: PLoS Genet. 2026 May 28;22(5):e1012172. doi: 10.1371/journal.pgen.1012172 (PMC13349305; doi:10.1371/journal.pgen.1012172)
Supplement: S3 Fig — (A) Dual heterozygote yap1kg151/+; wwtr1kg169/+ in-cross larvae reared at 28.5ºC in lateral view, dorsal to top, anterior to left. Yap1kg151/+;wwtr1kg169 fish have oedema (asterisks) from 2 dpf. In comparison to yap1kg151 single mutant, yap1kg151;wwtr1kg169/+ fish have more severe eye defects including coloboma starting at 2 dpf (arrowheads). Bar = 500 μm. (B) Wholemounts of genotypically-identified yap1kg151 mutant larvae reared at 28.5°C until 70% epiboly then at 32.0°C until 3 dpf. Compared to wild type and heterozygote siblings (wt, larva 1) (top), the severity of yap1kg151 phenotype varies, with oedema (larvae 2–6, red arrows), small eye pigment area (larvae 2–6) and bent (larvae 2–4) or curled (larvae 5,6, asterisks) bodies. Bottom: pericardial oedema (left) and eye pigmentation (right, outlined in yellow dots). Bars = 100 μm. (C) Penetrance of the small eye pigmentation/oedema/bent tail phenotype in fry from three yap1kg151/+ in-cross experiments (Exp 1–3) reared at different temperatures to the equivalent of the 5 dpf developmental stage, as indicated in schematic (top). Number of larvae on bars. Difference from 25% mutant phenotype: Χ2; 20.5°C p = 2 x 10^-10^, 28.5°C p = 3 x 10^-5^, 32.0°C p = 0.1. (D) Survival beyond 5 dpf-equivalent of yap1kg151 compared to siblings when raised at 28.5°C until 70% epiboly then 20.5°C until 7 days (5 dpf-equivalent) and 28.5°C thereafter. Data pooled from seven yap1kg151/+ in-crosses totalling 576 viable larvae at 7 days (no deaths occurred before 7 days), of which 423 survived to genotyping at 3 months (73%). (E) Poor survival beyond 5 dpf-equivalent of wwtr1kg169 pooled from three separate lays. * p-values of Χ2 tests performed comparing numbers of genotypes obtained against the numbers expected at 1:2:1. (F) Survival from 5 dpf to 3 months from a yap1kg151/+;wwtr1kg169/+ in-cross raised at 28.5°C. Note the lack of surviving yap1kg151/+;wwtr1kg169/kg169 larvae (p = 0.008; Χ2 on survival versus non-yap1 mutant siblings). Numb [file pgen.1012172.s003.pdf]

S3 Fig

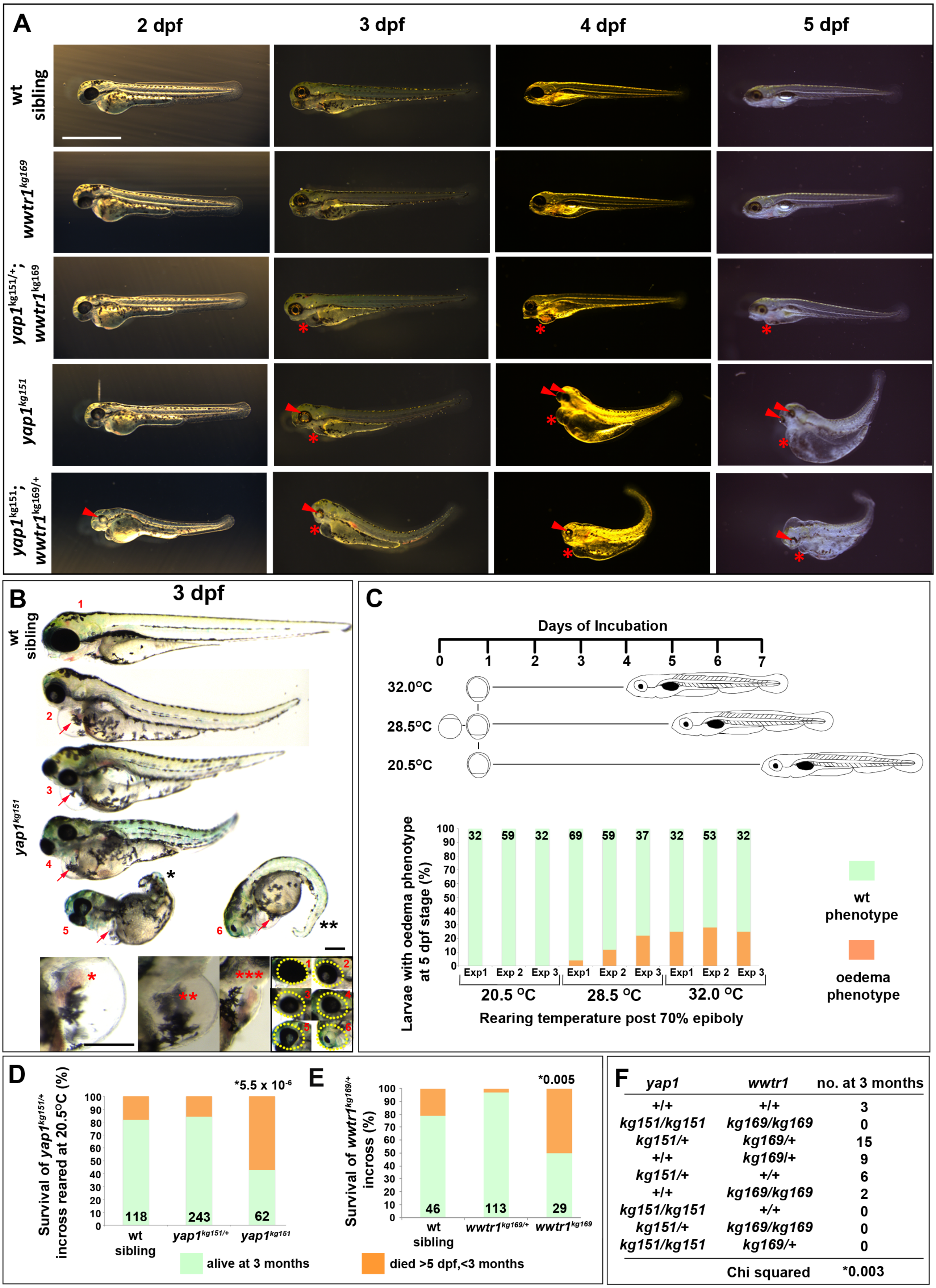

yap1	wwtr1	no. at 3 months
+/+	+/+	3
kg151/kg151	kg169/kg169	0
kg151/+	kg169/+	15
+/+	kg169/+	9
kg151/+	+/+	6
+/+	kg169/kg169	2
kg151/kg151	+/+	0
kg151/+	kg169/kg169	0
kg151/kg151	kg169/+	0
	Chi squared	*0.003

S3 Fig. Variable penetrance of severe *Yap1* and mild *Wwtr1* mutant phenotypes.

(A) Dual heterozygote *yap1*$^{kg151/+}$*;wwtr1*$^{kg169/+}$ in-cross larvae reared at 28.5ºC in lateral view, dorsal to top, anterior to left. *Yap1*$^{kg151/+}$*;wwtr1*^{kg169} fish have oedema (asterisks) from 2 dpf. In comparison to *yap1*^{kg151} single mutant, *yap1*^{kg151}*;wwtr1*$^{kg169/+}$ fish have more severe eye defects including coloboma starting at 2 dpf (arrowheads). Bar = 500 µm. **(B)** Wholemounts of genotypically-identified *yap1*^{kg151} mutant larvae reared at 28.5°C until 70% epiboly then at 32.0°C until 3 dpf. Compared to wild type and heterozygote siblings (wt, larva 1) (top), the severity of *yap1*^{kg151} phenotype varies, with oedema (larvae 2-6, red arrows), small eye pigment area (larvae 2-6) and bent (larvae 2-4) or curled (larvae 5,6, asterisks) bodies. Bottom: pericardial oedema (left) and eye pigmentation (right, outlined in yellow dots). Bars = 100 µm. **(C)** Penetrance of the small eye pigmentation/oedema/bent tail phenotype in fry from three *yap1*$^{kg151/+}$ in-cross experiments (Exp 1-3) reared at different temperatures to the equivalent of the 5 dpf developmental stage, as indicated in schematic (top). Number of larvae on bars. Difference from 25% mutant phenotype: X^2; 20.5°C $p = 2 \times 10^{-10}$, 28.5°C $p = 3 \times 10^{-5}$, 32.0°C $p = 0.1$. **(D)** Survival beyond 5 dpf-equivalent of *yap1*^{kg151} compared to siblings when raised at 28.5°C until 70% epiboly then 20.5°C until 7 days (5 dpf-equivalent) and 28.5°C thereafter. Data pooled from seven *yap1*$^{kg151/+}$ in-crosses totalling 576 viable larvae at 7 days (no deaths occurred before 7 days), of which 423 survived to genotyping at 3 months (73%). **(E)** Poor survival beyond 5 dpf-equivalent of *wwtr1*^{kg169} pooled from three separate lays. * *p*-values of X^2 tests performed comparing numbers of genotypes obtained against the numbers expected at 1:2:1. **(F)** Survival from 5 dpf to 3 months from a *yap1*$^{kg151/+}$*;wwtr1*$^{kg169/+}$ in-cross raised at 28.5°C. Note the lack of surviving *yap1*$^{kg151/+}$*;wwtr1*$^{kg169/kg169}$ larvae ($p = 0.008$; X^2 on survival versus non-*yap1* mutant siblings). Number of fish analysed is indicated on columns.
