# Supplementary material for: Yap1 regulates motility and vertebral development and prevents kyphoscoliosis in zebrafish
Source: PLoS Genet. 2026 May 28;22(5):e1012172. doi: 10.1371/journal.pgen.1012172 (PMC13349305; doi:10.1371/journal.pgen.1012172)
Supplement: S4 Fig — Heterozygote yap1kg151/+ carriers were crossed and reared at permissive temperature from 70% epiboly and analysed live (A) or after fixation (B) and are shown in lateral view, dorsal to top, anterior to left. (A) Dual heterozygote yap1kg151/+;Tg(actc1b:mCherryCAAX)pc22/+ and yap1kg151/+; Tg(actc1b:LIFEACT-EGFP)pc21/+ crossed and larvae reared to 6 dpf-equivalent had normal structure and average sarcomere length (brackets; 1.94 μm) in mutants and siblings. (B) α-actinin at 5 dpf-equivalent in larvae from an in-cross of heterozygote yap1kg151/+ parents. Bar = 5 μm. (PDF) [file pgen.1012172.s004.pdf]

S4 Fig

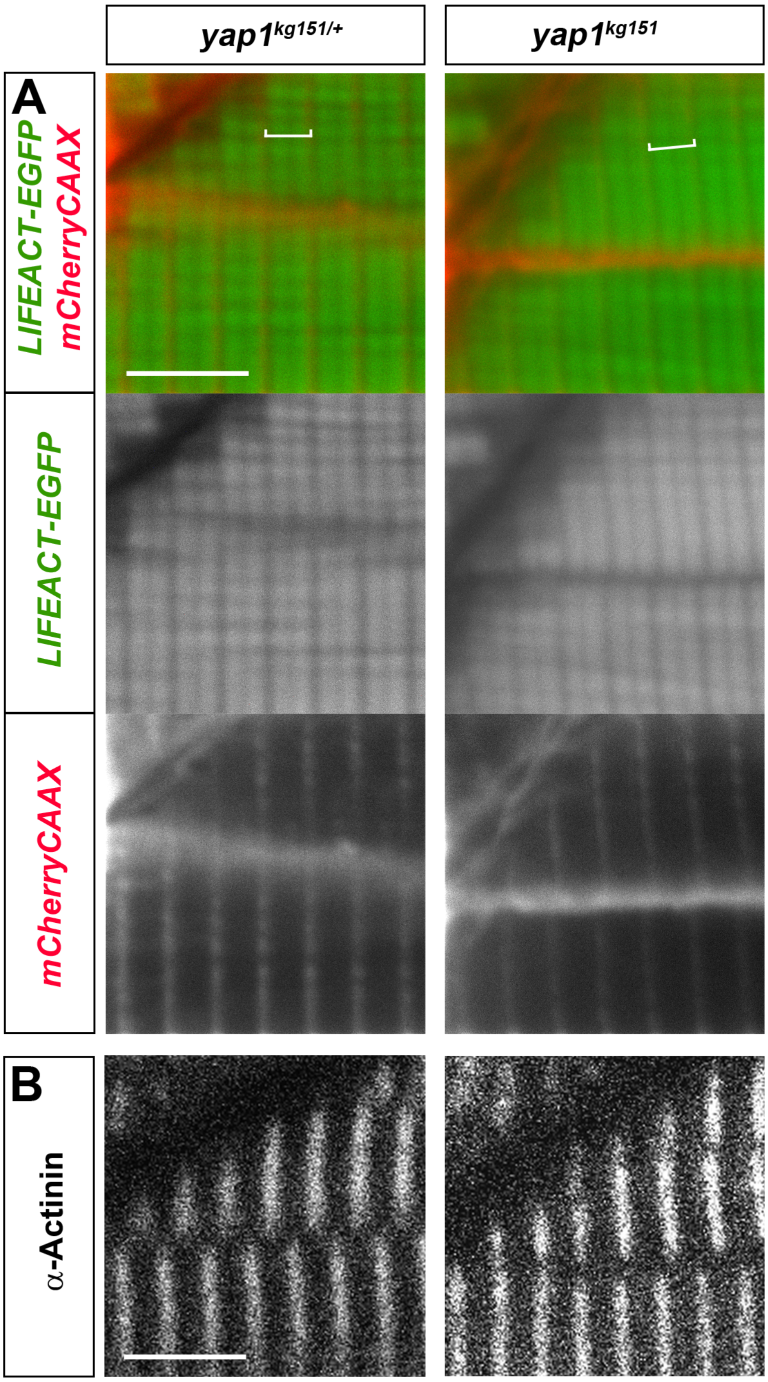

S4 Fig. Sarcomere organisation appears unaffected in *yap1*^{kg151} mutant.

Heterozygote *yap1*$^{kg151/+}$ carriers were crossed and reared at permissive temperature from 70% epiboly and analysed live (A) or after fixation (B) and are shown in lateral view, dorsal to top, anterior to left. **(A)** Dual heterozygote *yap1*$^{kg151/+}$*;Tg(actc1b:mCherryCAAX)*$^{pc22/+}$ and *yap1*$^{kg151/+}$*;Tg(actc1b:LIFEACT-EGFP)*$^{pc21/+}$ crossed and larvae reared to 6 dpf-equivalent had normal structure and average sarcomere length (brackets; 1.94 μm) in mutants and siblings. **(B)** α-actinin at 5 dpf-equivalent in larvae from an in-cross of heterozygote *yap1*$^{kg151/+}$ parents. Bar = 5 μm.
