# Supplementary material for: Yap1 regulates motility and vertebral development and prevents kyphoscoliosis in zebrafish
Source: PLoS Genet. 2026 May 28;22(5):e1012172. doi: 10.1371/journal.pgen.1012172 (PMC13349305; doi:10.1371/journal.pgen.1012172)
Supplement: S5 Fig — (A) Swimming velocity in mutants is not correlated with their length. (B) Maximum trunk angle shows no correlation with total fish length. (PDF) [file pgen.1012172.s005.pdf]

S5 Fig

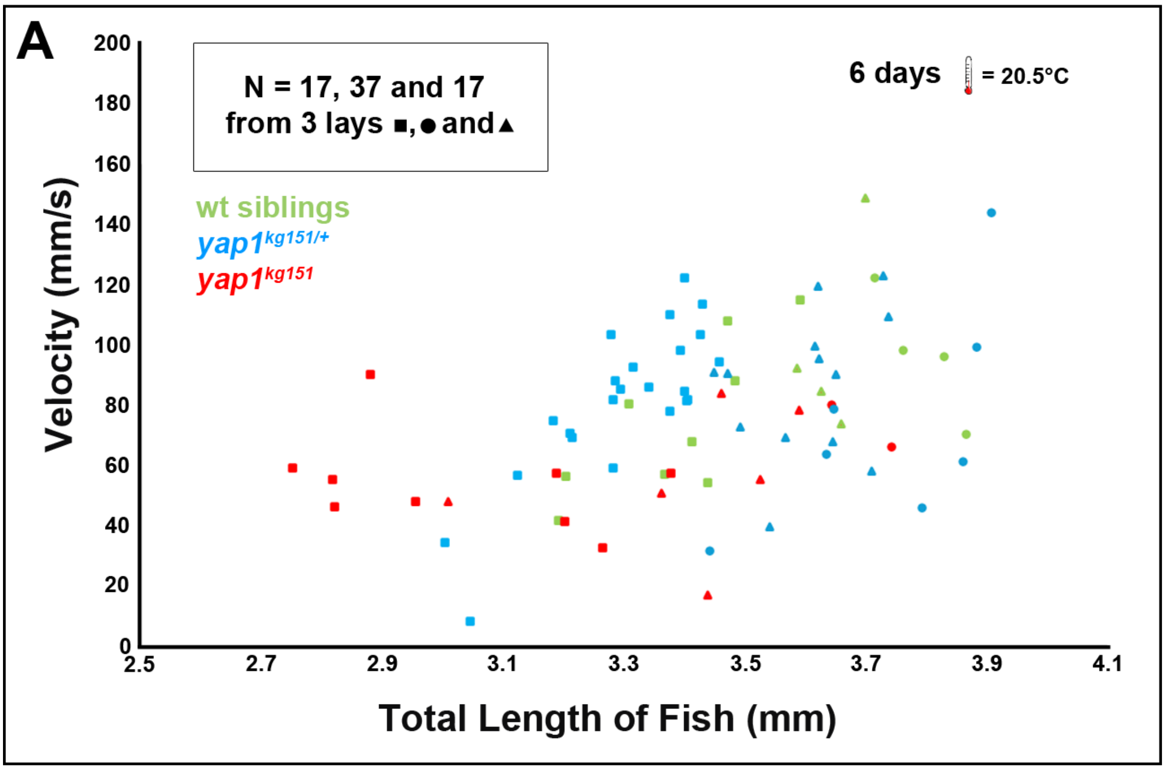

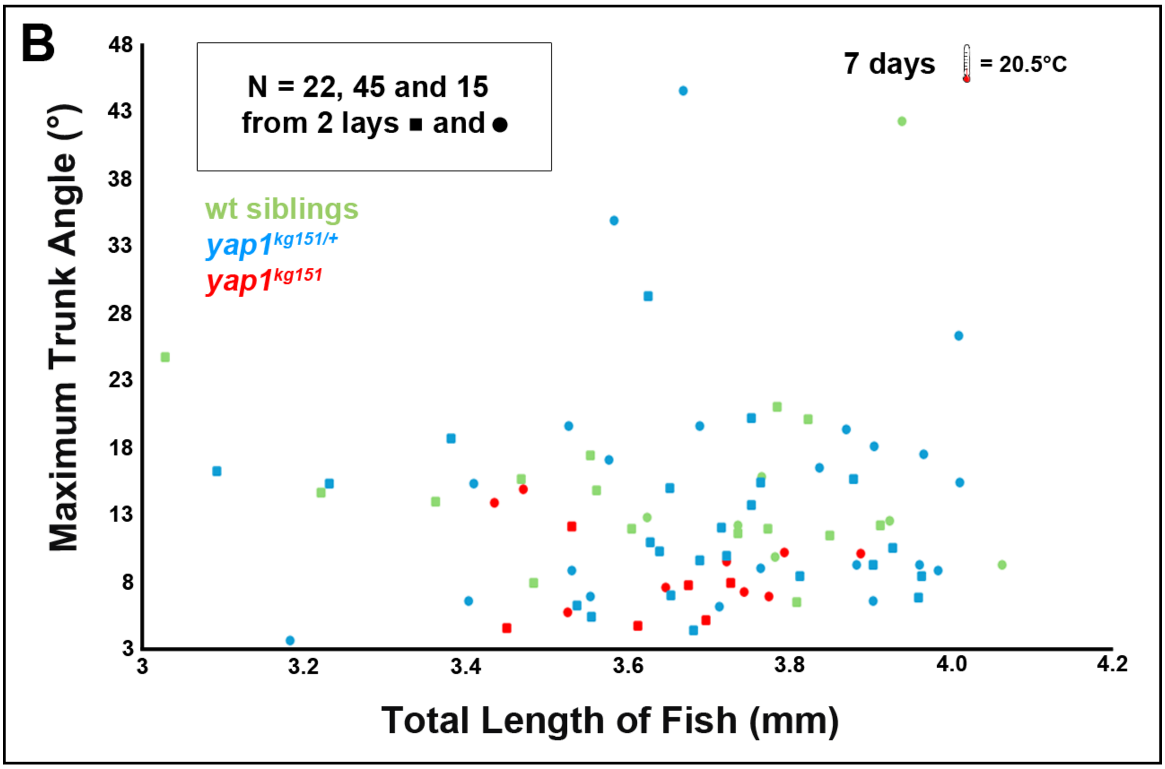

S5 Fig. *Yap1* mutant movement defects are not due to shorter fish length.

(A) Swimming velocity in mutants is not correlated with their length. **(B)** Maximum trunk angle shows no correlation with total fish length.
