# Supplementary material for: Yap1 regulates motility and vertebral development and prevents kyphoscoliosis in zebrafish
Source: PLoS Genet. 2026 May 28;22(5):e1012172. doi: 10.1371/journal.pgen.1012172 (PMC13349305; doi:10.1371/journal.pgen.1012172)
Supplement: S6 Fig — (A) Time course of development of embryos from a dual heterozygote yap1kg151/+;wwtr1kg169/+ in-cross. Lateral view, dorsal to top, anterior to left. Red brackets highlight reduced height of myotome, at 14ss and further reduction at 16ss. By 21ss, yolk elongation fails. Yellow dots marking somite borders highlight shorter length of somites in double mutants compared to siblings. (B) In situ mRNA hybridisation for MRF mRNAs in 16ss double mutant (right) and sibling (left). Dorsal flatmount, anterior to top. (C) Confocal stacks of flatmounted 18ss yap1kg151;wwtr1kg169 and sibling embryos stained for fast myosin (F310, red), slow myosin (F59, green), and nuclei (Hoechst 33342, blue). Fractions indicate number of genotyped flatmounts showing the phenotype. (D) Lateral view confocal stacks of 24ss yap1kg151;wwtr1kg169 and sibling larvae stained for fast myosin (F310, green). (E,F) Volume of myotome 17 at 2 dpf in progeny from a dual heterozygote yap1kg151/+;wwtr1kg169/+ in-cross (E) or a yap1kg151 mutant female crossed with a yap1kg151/+;wwtr1kg169/+ male (F), each grown at the non-permissive temperature. Numbers of fish analysed are indicated on columns. Bars: A = 200 μm; B = 100 μm; C and D = 50 μm. (PDF) [file pgen.1012172.s006.pdf]

S6 Fig

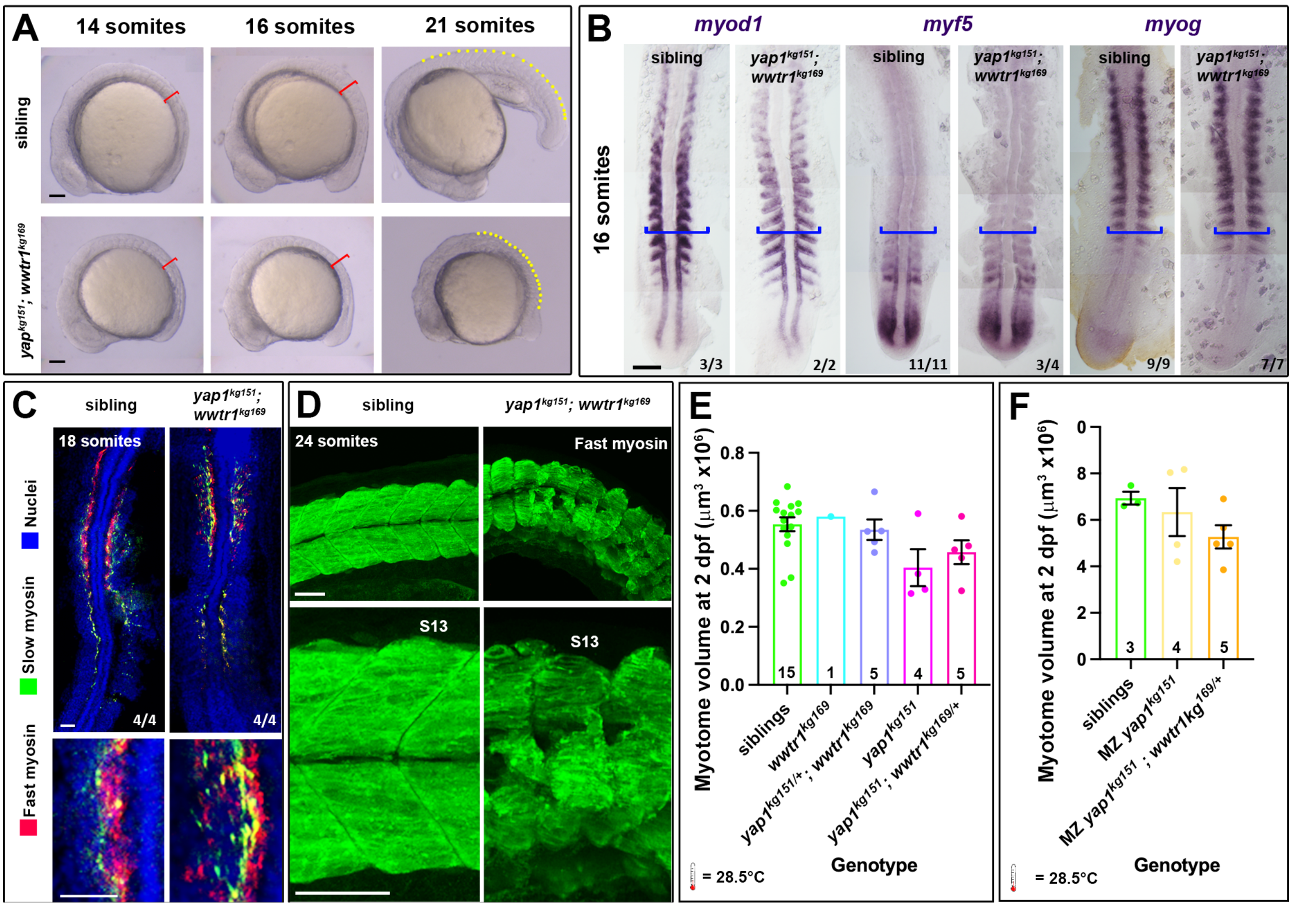

S6 Fig. Double mutants have normal myogenesis prior to developmental arrest.

(A) Time course of development of embryos from a dual heterozygote $yap1^{kg151/+}$;$wwtr1^{kg169/+}$ in-cross. Lateral view, dorsal to top, anterior to left. Red brackets highlight reduced height of myotome, at 14ss and further reduction at 16ss. By 21ss, yolk elongation fails. Yellow dots marking somite borders highlight shorter length of somites in double mutants compared to siblings. **(B)** In situ mRNA hybridisation for MRF mRNAs in 16ss double mutant (right) and sibling (left). Dorsal flatmount, anterior to top. **(C)** Confocal stacks of flatmounted 18ss $yap1^{kg151}$;$wwtr1^{kg169}$ and sibling embryos stained for fast myosin (F310, red), slow myosin (F59, green), and nuclei (Hoechst 33342, blue). Fractions indicate number of genotyped flatmounts showing the phenotype. **(D)** Lateral view confocal stacks of 24ss $yap1^{kg151}$;$wwtr1^{kg169}$ and sibling larvae stained for fast myosin (F310, green). **(E,F)** Volume of myotome 17 at 2 dpf in progeny from a dual heterozygote $yap1^{kg151/+}$;$wwtr1^{kg169/+}$ in-cross (E) or a $yap1^{kg151}$ mutant female crossed with a $yap1^{kg151/+}$;$wwtr1^{kg169/+}$ male (F), each grown at the non-permissive temperature. Numbers of fish analysed are indicated on columns. Bars: A = 200 µm; B = 100 µm; C and D = 50 µm.
