# Supplementary material for: Yap1 regulates motility and vertebral development and prevents kyphoscoliosis in zebrafish
Source: PLoS Genet. 2026 May 28;22(5):e1012172. doi: 10.1371/journal.pgen.1012172 (PMC13349305; doi:10.1371/journal.pgen.1012172)
Supplement: S7 Fig — (A-C). Weight, standard length and Fulton’s condition factor (k) of wwtr1kg169 (A), yap1kg137, yap1kg151 and yap1kg152 (B) and yap1kg151 (C). (D) Adult yap1kg137/kg151 and yap1kg137/kg152 mutants reared at the permissive 20.5ºC temperature until 5 dpf-equivalent and then at 26.5ºC are smaller than their wt or heterozygote siblings, points plotted with jitter to avoid overlap. Statistically significant results of Kruskal-Wallis tests are shown (B,C). (PDF) [file pgen.1012172.s007.pdf]

## S7 Fig

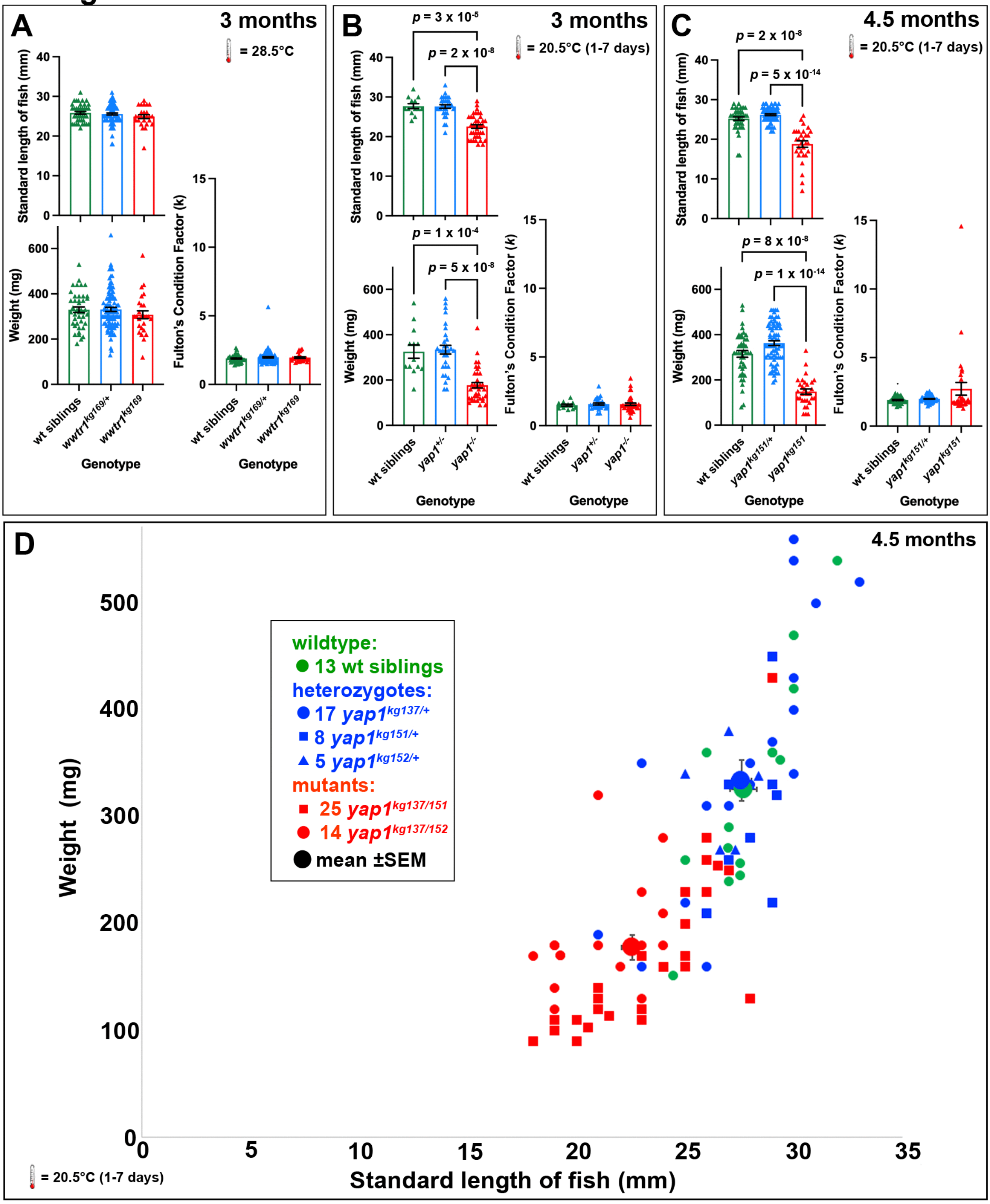


**S7 Fig. Adult *yap1*$^{kg137/151}$ and *yap1*$^{kg137/152}$ mutants are smaller than their siblings.**

**(A-C)**. Weight, standard length and Fulton's condition factor (k) of *wwtr1*$^{kg169}$ (A), *yap1*$^{kg137}$, *yap1*$^{kg151}$ and *yap1*$^{kg152}$ (B) and *yap1*$^{kg151}$ (C). **(D)** Adult *yap1*$^{kg137/151}$ and *yap1*$^{kg137/152}$ mutants reared at the permissive 20.5ºC temperature until 5 dpf-equivalent and then at 26.5ºC are smaller than their wt or heterozygote siblings, points plotted with jitter to avoid overlap. Statistically significant results of Kruskal-Wallis tests are shown (B,C).
