# Supplementary material for: Yap1 regulates motility and vertebral development and prevents kyphoscoliosis in zebrafish
Source: PLoS Genet. 2026 May 28;22(5):e1012172. doi: 10.1371/journal.pgen.1012172 (PMC13349305; doi:10.1371/journal.pgen.1012172)
Supplement: S8 Fig — Notochord cells of yap1kg151 mutants grown at 20.5°C until 3 day (equivalent to ~2 dpf) were indistinguishable from those of their wild type siblings. Box indicates location of magnified images, showing variability between individuals unrelated to genotype. Bars = 100 μm. (PDF) [file pgen.1012172.s008.pdf]

S8 Fig

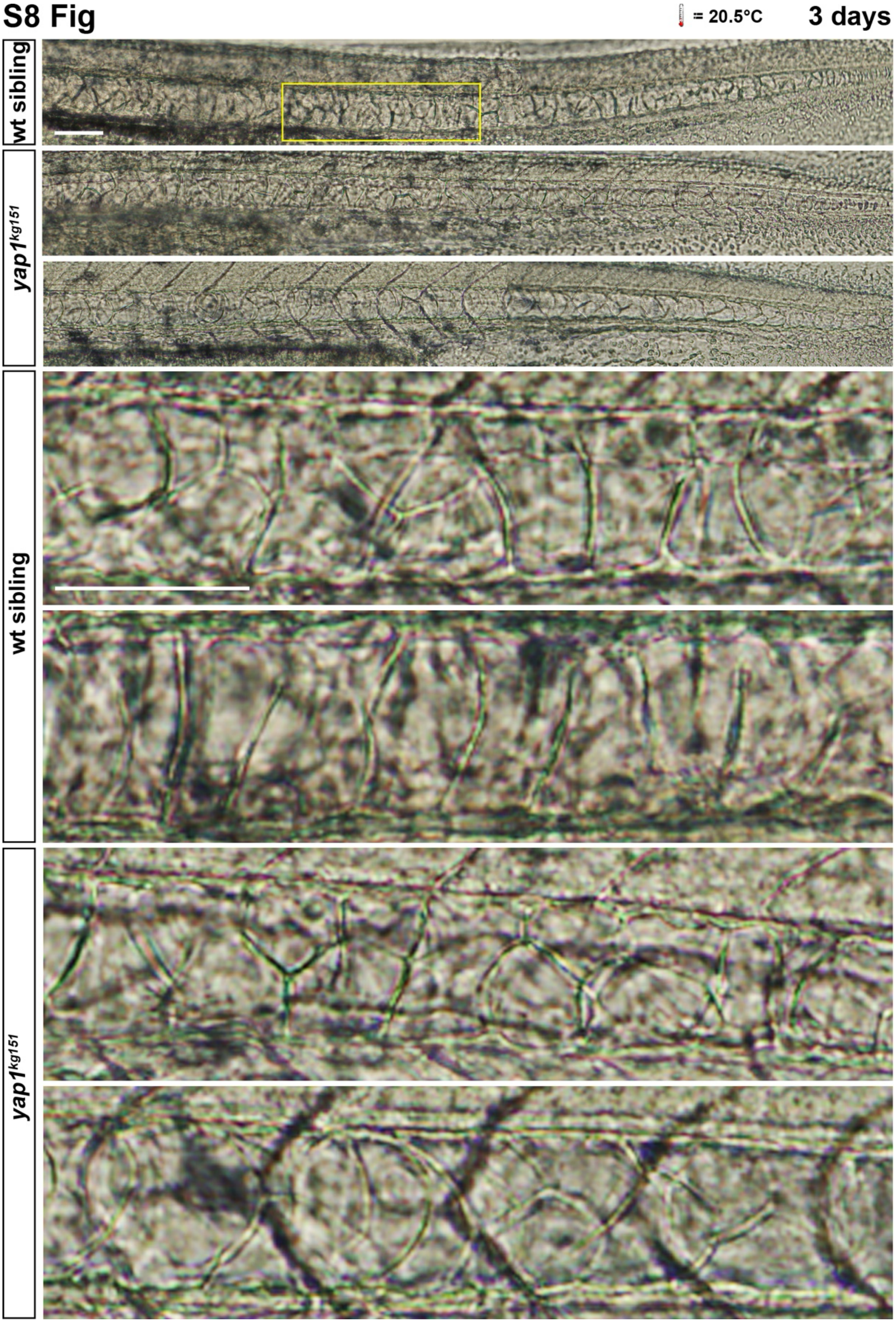

S8 Fig. Apparently normal notochord cells in *yap1*^{kg151} mutant.

Notochord cells of *yap1*^{kg151} mutants grown at 20.5°C until 3 day (equivalent to ~2 dpf) were indistinguishable from those of their wild type siblings. Box indicates location of magnified images, showing variability between individuals unrelated to genotype. Bars = 100 μm.
