# Supplementary material for: Yap1 regulates motility and vertebral development and prevents kyphoscoliosis in zebrafish
Source: PLoS Genet. 2026 May 28;22(5):e1012172. doi: 10.1371/journal.pgen.1012172 (PMC13349305; doi:10.1371/journal.pgen.1012172)
Supplement: S9 Fig — (A) Schematic dorsal view (left) of adult zebrafish brain showing the sagittal dissection (red line), and lateral view (right) of the dissected brain with the position of imagining in the rhombencephalic ventricle marked (red box). (B-D) Scanning electron micrographs of the internal surface of the rhombencephalic ventricle in sibling wildtype (B) and yap1kg151 mutants without (C) or with (D) spinal curvature phenotype at two magnifications. OB; olfactory bulb, Tel; telencephalon, TeO; optic tectum, CCe; corpus cerebelli, CC; crista cerebralis, V; ventricle. Bars = 10 μm. (PDF) [file pgen.1012172.s009.pdf]

S9 Fig

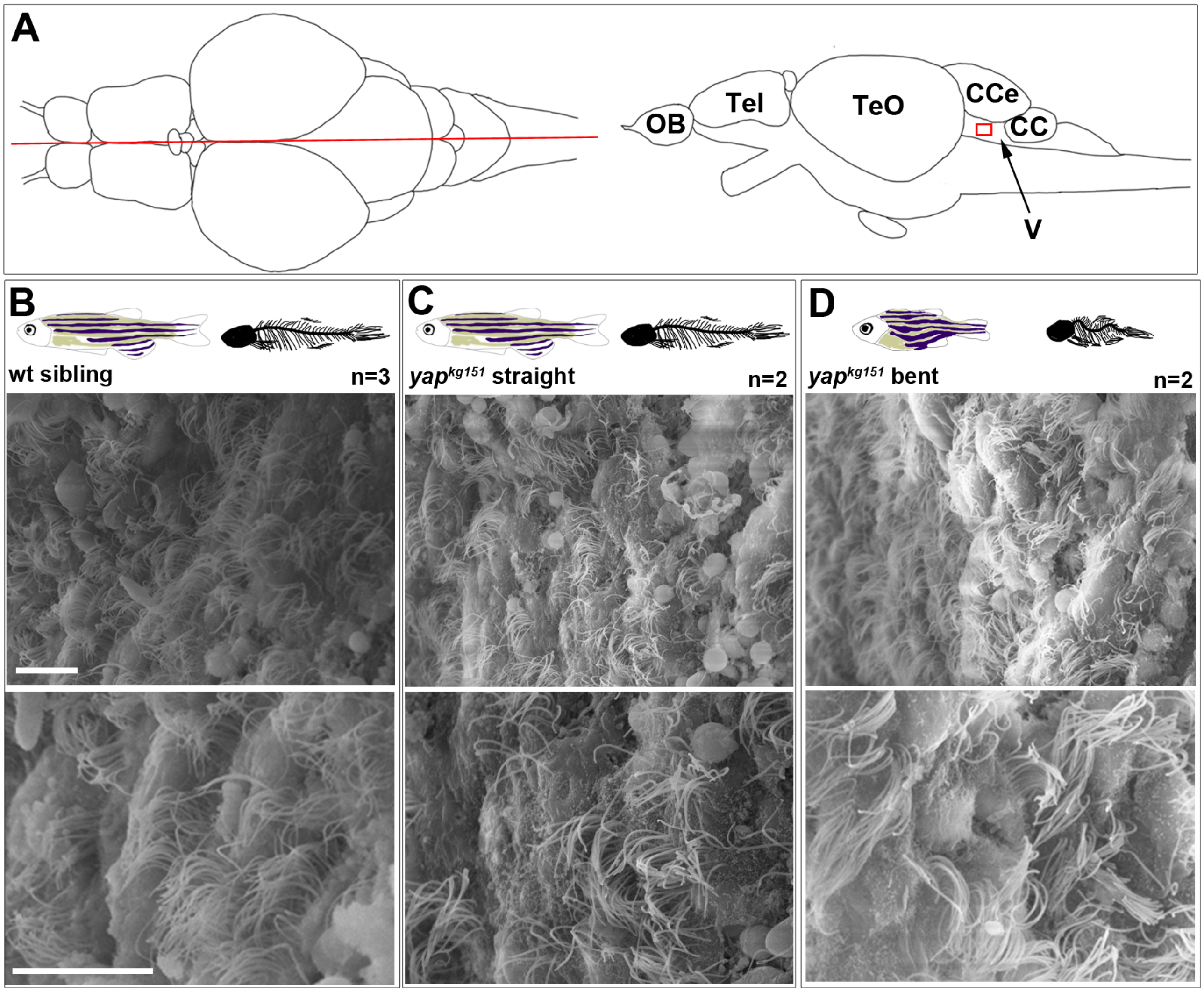

S9 Fig. Zebrafish *yap1*^{kg151} mutants lack a morphological ciliary defect in cerebral ventricle.

(A) Schematic dorsal view (left) of adult zebrafish brain showing the sagittal dissection (red line), and lateral view (right) of the dissected brain with the position of imagining in the rhombencephalic ventricle marked (red box). **(B-D)** Scanning electron micrographs of the internal surface of the rhombencephalic ventricle in sibling wildtype (B) and *yap*^{kg151} mutants without (C) or with (D) spinal curvature phenotype at two magnifications. OB; olfactory bulb, Tel; telencephalon, TeO; optic tectum, CCe; corpus cerebelli, CC; crista cerebralis, V; ventricle. Bars = 10 μm.
