# Supplementary material for: Yap1 regulates motility and vertebral development and prevents kyphoscoliosis in zebrafish
Source: PLoS Genet. 2026 May 28;22(5):e1012172. doi: 10.1371/journal.pgen.1012172 (PMC13349305; doi:10.1371/journal.pgen.1012172)
Supplement: S10 Fig — Immunofluorescent detection of AFRU Reissner’s fibre antigen (green, arrowheads) and nuclei (blue) in the posterior spinal canal at the 5 dpf-equivalent stage in single optical slices (top) or maximum intensity projection in a genotyped yapkg151 mutant. Single channels are shown beneath in grayscale. Note the intense AFRU signal at the posterior tip of the spinal cord (arrows). Bar = 50 μm. (PDF) [file pgen.1012172.s010.pdf]

S10 Fig

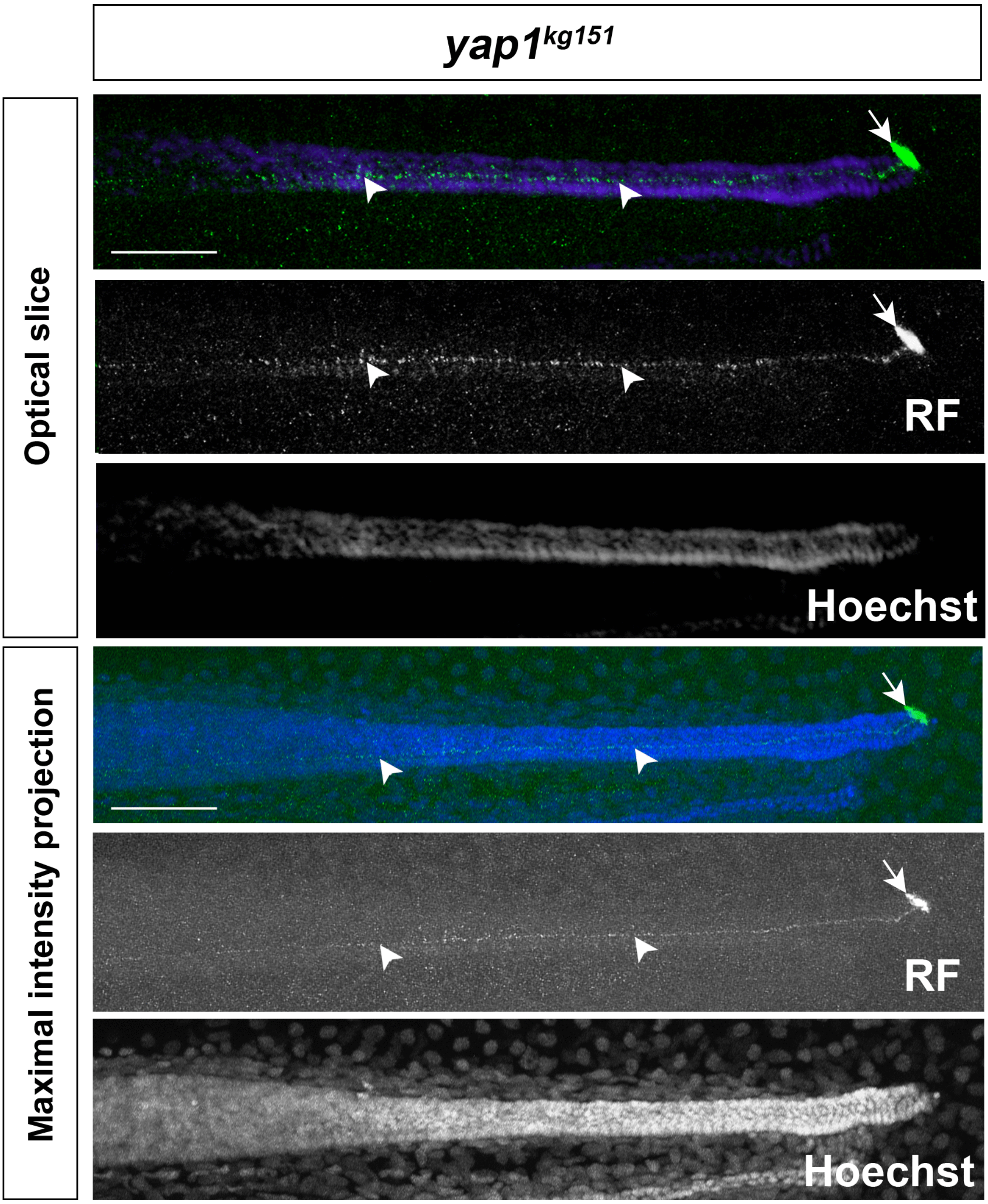

S10 Fig. Reissner fibre is present in *yap1^{kg151}* mutant.

Immunofluorescent detection of AFRU Reissner's fibre antigen (green, arrowheads) and nuclei (blue) in the posterior spinal canal at the 5 dpf-equivalent stage in single optical slices (top) or maximum intensity projection in a genotyped *yap^{kg151}* mutant. Single channels are shown beneath in grayscale. Note the intense AFRU signal at the posterior tip of the spinal cord (arrows). Bar = 50 μm.
