# Supplementary material for: Yap1 regulates motility and vertebral development and prevents kyphoscoliosis in zebrafish
Source: PLoS Genet. 2026 May 28;22(5):e1012172. doi: 10.1371/journal.pgen.1012172 (PMC13349305; doi:10.1371/journal.pgen.1012172)
Supplement: S11 Fig — In situ mRNA hybridisation for col8a1a mRNA in additional sibling 36 hpf embryos to those shown in Fig 6B. (A) Mutants show patchy axial distribution in hypochord (arrowheads), but more even signal in notochord (arrows). Hypochord signal is absent for one or more whole somite lengths in mutants. (B,C) In contrast, heterozygous (B) and wild type (C) siblings show continuous hypochord signal throughout the axis at higher level than that in notochord. Bar = 100 μm. (PDF) [file pgen.1012172.s011.pdf]

S11 Fig

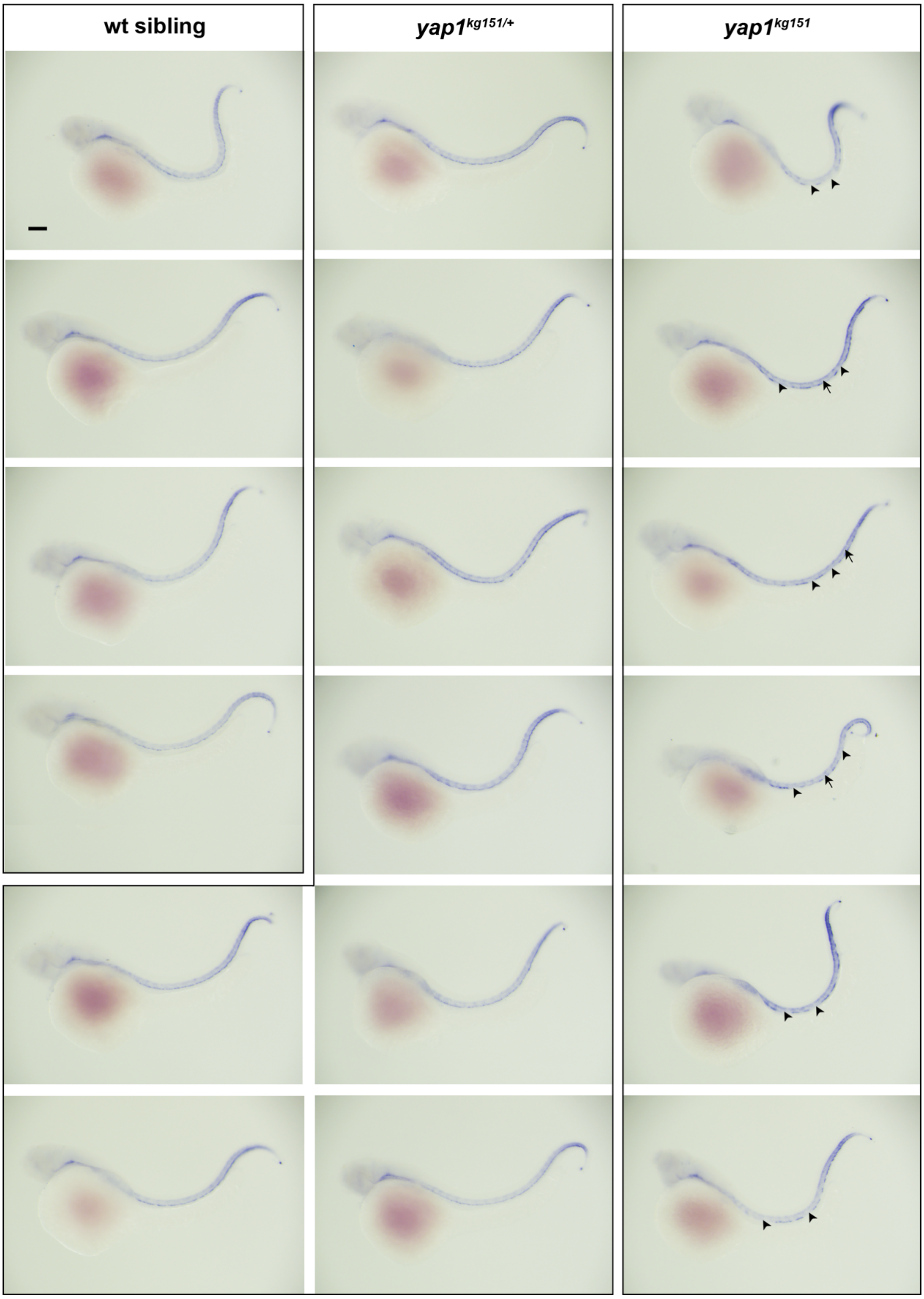

S11 Fig. Defective distribution of *col8a1a* mRNA in hypochord of *yap1^{kg151}* mutants.

In situ mRNA hybridisation for *col8a1a* mRNA in additional sibling 36 hpf embryos to those shown in Fig 6B. **(A)** Mutants show patchy axial distribution in hypochord (arrowheads), but even signal in notochord (arrows). Hypochord signal is absent for one or more whole somite lengths in mutants. **(B,C)** In contrast, heterozygous (B) and wild type (C) siblings show continuous hypochord signal throughout the axis at higher level than that in notochord. Bar = 100 μm.
