# Supplementary material for: Yap1 regulates motility and vertebral development and prevents kyphoscoliosis in zebrafish
Source: PLoS Genet. 2026 May 28;22(5):e1012172. doi: 10.1371/journal.pgen.1012172 (PMC13349305; doi:10.1371/journal.pgen.1012172)
Supplement: S1 Table — (PDF) [file pgen.1012172.s013.pdf]

S1 Table. Staging of fish reared at 28.5ºC until 70% epiboly and at 20.5ºC thereafter.

Age in days	Equivalent @ 28.5ºC	Length (mm)
1	10 som	
2	prim 6	2.0
3	prim 20	2.6
4	high-pec	2.9
5	pec-fin	3.3
6		3.6
7	Swim bladder inflated	3.8
