# Supplementary material for: Yap1 regulates motility and vertebral development and prevents kyphoscoliosis in zebrafish
Source: PLoS Genet. 2026 May 28;22(5):e1012172. doi: 10.1371/journal.pgen.1012172 (PMC13349305; doi:10.1371/journal.pgen.1012172)
Supplement: S2 Table — (PDF) [file pgen.1012172.s014.pdf]

S2 Table. Primers used in qRT-PCR and to make probes for in situ mRNA hybridisation.

Table S2A				
Gene	Forward	Reverse	Size (bp)	location
yap1	CCTCTTACGAGATACCTGAT	TCGTCTGGTCATTATGGTTT	102	exons 2-3
wwtr1	CCAGAAGTACTTTCTCAATCAC	TTGGAGACCTGATTATGGAG	108	exons 1-2
ef1a	AGCAGCAGCTGAGGAGTGAT	CCGCATTTGTAGATCAGATGG	137	exons 1-2

Table S2B				
Gene	Forward	Reverse	Size (bp)	location
yap1	TAATACGACTCACTATAGGGAGAACGCCGCCAGAGCCAAAGTCC	GGATCCATTAACCCTCACTAAAGGGAAGTGCCGCCATCCTGCTCCAT	728	exons 1-6
wwtr1	TAATACGACTCACTATAGGGAGAGGACAACATGACAAACGCCT	GGATCCATTAACCCTCACTAAAGGGAACAGCTTTAGGTCCCCACTGA	1576	5'UTR-3'UTR
col8a1a	TAATACGACTCACTATAGGGAGATGGCTTGCCAGGAATAGGAA	GGATCCATTAACCCTCACTAAAGGGAAAAAAACCCGGAGAAGGAGGAG	1607	exon 2
col9a1b	TAATACGACTCACTATAGGGAGAATGGGAATGAACAGCTGGGA	GGATCCATTAACCCTCACTAAAGGGAACGTTCACCTTGTTTGCCTGA	1129	exons 4-18
fmyhc1.2	TAATACGACTCACTATAGGGAGAGATCAGCCAGGGTTGACTGT	GGATCCATTAACCCTCACTAAAGGGAATGGCGGCTTACTTCTTACCA	77	5' UTR
fmyhc2.1	TAATACGACTCACTATAGGGAGAAATCATTCATCTGGTCTCAAGGA	GGATCCATTAACCCTCACTAAAGGGAAGCCATCTCCGCGTCAGTA	65	5' UTR
