# Supplementary material for: Yap1 regulates motility and vertebral development and prevents kyphoscoliosis in zebrafish
Source: PLoS Genet. 2026 May 28;22(5):e1012172. doi: 10.1371/journal.pgen.1012172 (PMC13349305; doi:10.1371/journal.pgen.1012172)
Supplement: Attachment — Submitted filename: ResponsetoReviewers260417.docx [file pgen.1012172.s015.docx]

**We appreciate the careful consideration of our manuscript by the Reviewers and Editors. We believe we can effectively answer the points raised and that the manuscript is now significantly improved by taking into account the thoughtful comments of Reviewers 1 and 2. As non-experts in scoliosis, it is very helpful to receive a more ‘insider’ perspective on the issues that preoccupy those in the field.**

**Reviewer #1:** This article investigates the role of Hippo signaling in zebrafish through mutations in yap1 and wwtr1. The authors show that yap1 and wwtr1 are expressed in somatic muscle, and that ablation of each gene individually produces only minor defects in larval trunk movements or mobility, with no obvious defects in muscle development or gross architecture. Functionally, wwtr1 mutants display normal larval swimming velocity, whereas yap mutants exhibit impaired swimming, indicating that yap contributes to functional swimming behaviors. Additionally, yap mutants raised at the restrictive temperature develop scoliosis in some individuals, despite an absence of obvious musculature defects. The onset of abnormal body curvature occurs at late larval stages and is accompanied by subtle morphologic changes in the vertebral bodies in adulthood. Finally, yap is required for normal larval growth (including overall body length) independent of scoliosis. While the study does not reveal a clear mechanism for scoliosis onset, its quantitative approach suggests that small differences in growth trajectories may destabilize tissues required to maintain a straight spine, with yap playing a contributing role. I would recommend acceptance of the manuscript after revisions, as suggested by other reviewers.

Concerns to address:

Introduction: Please re-think the statement that scoliosis reduces quality of life for over 3% of people. This is at face value simply not true. In truth only about 40K surgeries are performed in the US for scoliosis per year so the number are just not correct here. In fact, while some people due experience psychosocial distress at having a curvy spine and may require bracing or surgery, in truth most people with scoliosis have no noticeable effects associated with scoliosis. Moreover, I have met people with scoliosis that have challenged my own language about how this disorder is a ‘burden’, so please be mindful of your language.

**We thank the Reviewer for alerting us to these issues. We have now modified our opening sentence to remove mention of QoL and to include data on the lifetime rate of spinal surgical intervention for scoliosis (~0.1% in high income countries).**

Figure 2: Makes a claim that wwtr1 mutants are ‘defective in early larval movement’. However, swimming velocity is not affected at 5 or 11 dpf? Thus, this claim stands only on the evidence of a reduction in trunk angle measures in larval fish after electrical stimulation. I would question, if this change in trunk angle does not attribute to a functional measure of movement such as swimming velocity, if the conclusion is well supported?

**We accept the Reviewer’s point. We have now repeated the swimming velocity analysis and confirm that there is no difference in swimming velocity at 5 dpf. We have therefore modified the title of Figure 2 and Results text to indicate that differences in what the two assays measure might contribute to the observed difference in observed effect size. We write: *‘Whereas electrically-evoked tail movement likely reflects a high force regime in which movement may be limited by altered trunk structure or extracellular matrix deposition, swimming speed may represent a lower force regime under which a motility defect is not apparent.’***

Figure 3: Makes a claim that yap1 mutants are ‘defective in neuromuscular function’. Again I don’t think this conclusion is supported by the evidence. Instead, I only see changes in trunk angle measure, that are now supported by reduction in larval movement. This suggest that instead yap1 mutants are defective in larval movement, as stated for Figure 2. I think you would need to demonstrate that muscle or the neuromuscular junctions were effected to make the claim above.

**We accept this criticism and have modified the title of the legend to Figure 3 accordingly. We used ‘neuromusculoskeletal function’ to indicate that, at this point in the manuscript, we do not know if there is a muscle-response or a neural-drive problem. But we agree the term is ambiguous because many specialists immediately think of a NMJ defect when such a phrase is used.**

Line 285-286: ‘yap1 and wwtr1 have significant motility defects’ As I argued for Figure 2 , I don’t think an issue with trunk angle measures can translate to significant motility defects for wwtr1 mutants, if they functional are able to swim at a velocity also attributed with wild type animals. How is this conclusion supported by evidence?

**We have now rewritten this sentence to indicate that *yap1* mutant motility defects are more severe than those in *wwtr1* mutants.**

Lines 269 and 279: “staged in days, not dpf” Can you explain your metrics for staging? Standard length, myotome number, ect? It is very important to be concise about this so that others can learn what you did and perhaps do the same one day if they needed to.

**Good point. We have now added further explanation in the M+M and Table S1, which shows the staging of embryos/larvae reared at 28.5ºC until 70% epiboly and then at 20.5ºC thereafter until 7 days (5 dpf-equivalent).**

Given that Yap1 has hydrocephalus in mice and that central canal cilia defects are associated with scoliosis in zebrafish. I am not sure that these cilia are not contributing to the larval onset of scoliosis, potentially destabilizing the Reissner fiber and causing scoliosis. IT is not the same to look at multiciliate cells in the brain as these cilia do not even form in zebrafish until much later than 13dpf (PMID: 26991819). Plus, the more phenotypic yap1 mutant embryos do display phenotypes associated with cilia defects, including edema and curved bodies. It may be interesting to look at the central canal motile cilia in these mutants.

**These are helpful points.** **We have now obtained antibodies to the Reissner’s fibre and find that it is still immunodetectable as a thin thread in 5 dpf *yap1* mutant, which we show in a new S10 Fig. We have been unable to examine cilia in the spinal canal as suggested owing to the lack of a suitable high sped camera/microscope set-up on our campus. We now discuss this new data and highlight that we have not looked a spinal canal cilia in the paragraphs on lines 422-427 and 711-717.**

Figure 4: The cartoon schematic is difficult to read. Please use white lettering or pull them out from the blue vertebral body background to make them easily seen. I would also suggest a bracket outside the vertebral body to represent the posterior height metric.

**Done.**

**Reviewer #2:** The manuscript by Williams-Ward et al Williams-Ward et. al unravels how YAP1 determines the motility and development of kyphoscoliosis in zebrafish. Scoliosis affects over 3% of people and is influenced by genetic, environmental, and mechanical factors. This study investigated the role of the mechanosensitive Hippo pathway genes yap1 and wwtr1 in zebrafish. The Authors found that loss of yap1 impaired growth, caused early motility defects, and led to vertebral abnormalities. Although initial defects were mild, many mutants later developed kyphoscoliosis, likely due to a positive mechanical feedback process. The findings suggest that Yap1 plays a key role in mechanosensory feedback between muscle, bone, and tendon to regulate vertebral growth and prevent spinal deformities.
The manuscript is interesting and addresses the role of the Yap1 and Wwtr1 in scoliosis. The Authors do provide some intriguing findings, although the mechanistic aspects are weak. Overall, the manuscript may be revised to address the specific comments below and reconsidered.

**We appreciate the careful reading and analysis of our manuscript, and the recognition that, although our mechanistic understanding is limited, the fundamental discovery of a Yap1 impact on the enigmatic origins of scoliosis may be of scientific and clinical importance and utility.**

Major comments
1. The representative data for the wwtr1kg133/+ genotype should be included in Figure 2A. Are the gaps between muscle fibers increased in the wwtr1kg133 genotype at 3 and 5 dpf?

**We now include heterozygote images. The apparent gaps in the mutant simply reflect the animal-to-animal variation in mosaicism of the membrane-EGFP reporter; we now explain this in the legend.**

2. At the functional level, YAP and TAZ are effectors of the Hippo pathway, leading to binding to TEAD and target gene activation; are the effects shown here mediated by TEAD?

**Yap1 and Taz/Wwtr1 interact with various transcription factors, including Runx and p73, not only TEADs (Kim et al 2018). There are five TEADs in zebrafish (1a, 1b, 3a, 3b and 4), at least three of which are expressed during early somitic myogenesis (Tead 1a, 3a and 3b). We have previously analysed the expression of Tead3a in muscle (Mann et al 2007). Tead1a or Tead3a morpholino knockdown apparently affects cilia in Kupffer’s vesicle (Fillatre et la 2019). Tead1b morphants show reduced eye pigment (Ha et al 2023), similar to that in Yap1 knockdown (Miesfeld et al 2015). Crispant analysis with Tead1a and Tead1b sgRNAs show RPE reduction (Buono et la 2021) and hindbrain patterning defects (Cayuso et al 2019). However, for none of these genes was a clean genetic analysis been reported until last year, when a viable *tead1a* mutant was ‘sicker and slimmer’, neutropaenic and showed reduced long term survival, but scoliosis was not reported (Yiqin et al 2025). Moreover, TEAD proteins are known to be regulated in many ways other than by the Yap1/Wwtr1/Hippo pathway, for example by Wnt, TGF-ß and EGFR pathways and through interactions with Vgll, Mef2, Srf and Max transcription factors (reviewed by Huh et al 2019; Currey et al 21). We therefore believe that functional studies on each individual Tead go beyond what can reasonably be expected in the current manuscript – particularly given the late-stage scoliotic phenotype that is our primary interest. We now briefly highlight the TEAD issue in the section of our manuscript discussing the cell autonomy of Yap1 action**

3. Have the Authors checked if Yap1 expression changes in the Wwtr1 mutant and vice versa?

**We have, and they do not. This is now shown in Fig S2H and mentioned in Results lines 253 and 308.**

4. The authors mention that yap1kg151 mutants show no possible defects in the notochord formation. However, are the vacuolated and epithelial cells able to secrete collagen and ECM comparable to wild types. Can authors comment on the expansion of these cells?

**We did/do not rule out the possibility of a notochord defect. We have now examined notochord morphology and expansion in more detail and can detect no defects at early stages. This is now explicitly stated on line 402. We have also performed mRNA in situ hybridisation on young mutants for various collagens (new Fig 6). *Col9a1b* mRNA, which accumulates strongly in notochord, revealed no difference from wild type siblings (Fig 6A). In marked contrast, *col8a1a* mRNA, which is expressed in chordoneural hinge, notochord and more strongly in floor plate and hypochord, revealed normal notochord expression but a striking patchy expression in the hypochord domain at 36 hpf stage (Fig 6B). This important novel finding suggests that *yap1* mutation may lead to local defects in hypochord-dependent signalling that could affect local vascular development and the oedema phenotype of mutants. We now present these findings in Fig 6 and S11 Fig and a new section of the Results (lines 428-438) and discuss them in lines 532-538. Whether an early hypochord defect could also predispose the larva to local scoliosis at later stages is unclear. While a fuller analysis of this phenotype goes beyond what is possible in the current manuscript, we now discuss how this important and apparently fully-penetrant finding may provide insight into the aetiology of focal scoliosis in a subset of mutant individuals in our Discussion (lines 644-650).**

5. Is the shortening of the embryonic axis dependent on Dstyk (dual serine/threonine and tyrosine protein kinase) activity?

**We presume this comment refers to the work of Sun et al (2020) in which Dstyk mutation causes early axis shortening and subsequent scoliosis. There are a number of differences between *yap1* and *dstyk* mutants, most prominent are the early shortening of the axis at 2 dpf onwards and the kinking of the tail from 7 dpf in *dstyk* mutants, two phenotypes we do not observe. Moreover, Sun et al propose that Dstyk works in notochord by inhibiting TORC1 and leading to defective notochord vacuolation and they show downregulation of *col9a1b* and *col8a1a* mRNAs in notochord. We have done experiments manipulating TORC1 activity in the zebrafish larva and have not observed notochord defects, even in conditions in which muscle growth is affected (Yogev e al 2013; Kelu et al 2020; Kelu and Hughes 2025). We did not observe altered vacuolation in *yap1* mutant notochords (Figs 2A and S8). Thus, there is no indication that our later shortening (arising at 13-17 days depending on rearing conditions) arises from effects of Yap1 mutation on Dstyk. Moreover, our novel phenotype in *col8a1a* mRNA in hypochord appears distinct from the global downregulation of both *col8a1a* and *col9a1b* observed with *dstyk* mutants. We have now added a specific discussion of Dstyk and other mutations that affect early notochord formation and cause scoliosis at lines 651-658.**

6. The authors have shown the expression profiles of myod and myog in the flat mounts (Fig S5B). Are the expression profiles of myosin heavy chain genes affected in the yap1kg151 mutants?

**Myh gene expression in zebrafish fast muscle is not very well characterised. We have not observed changes in antibody reactivity with the commonly-used fast and slow-specific monoclonals such as S58, F59, EB165. Moreover, there is no identified orthologue to Myh3 in zebrafish. We have now done ISH with unique 5’UTR probes to two fast cluster *fmyhc* genes that have interesting axial regionality and observe no differences in *yap1* mutants (Fig. 6C,D).**

7. Previous studies have shown the importance of Reisnerr’s fibers (RF) in axial development (H Lu, 2020; Troutwine BR, 2020). Is the formation of RF normal in the mutants?

**We have now obtained antibodies to the RF and can detect no defect in *yap1* mutant larvae. The data are now shown in Fig S10, described on lines 422-427, and discussed on lines 711-718.**

8. Is the sarcomere organisation defective in yap1kg151 mutants?

**No. This is now presented in Fig S4 at the stage we see motility defects. We have examined sarcomeres in detail using live lays marked with muscle-driven LIFEACT-EGFP and membrane mCherryCAAX, and in fixed larvae stained for alpha-actinin in Z-line. In both cases, larvae were imaged blind, photographed, and then genotyped retrospectively.**

Minor comment:
I would highly recommend authors to change the arrangement of figures to sequential manner.

**We take this to mean to make our order of presentation of data sequential in terms of animal age. We struggled with this, and would have liked to do it, as it is clearly the most logical order. However, it is highly desirable to compare and contrast *yap1* and *wwtr1* mutants and as many experiments were not done on *wwtr1* (owing to the milder phenotype), we found that the motility and survival data are best presented together at the start, before we go more deeply into the *yap1.* Moreover, the temperature sensitivity of *yap1* mutants and the need to compare experiments at different temperatures where they have been done also constrained the order. Lastly*,* because the early stage observations are not clearly causal for later ones, we feel such a neat order could be misleading. For all these reasons, we elect to retain the present order.**

**Reviewer #3:** In their study, Williams-Ward and colleagues have used global deletion mutants of yap1 and wwtr1 to describe a kyphoscoliosis phenotype in adults (most pronounced in yap1 mutants). Compound genetic analysis confirm existing data that these factors have some degree of redundant function. While the manuscript is well written and the data are high quality, the study falls short of making a significant contribution to understanding the cell types that require yap1/wwtr1 for proper spine development. Given the less than full penetrance of the yap1 spine phenotype, the authors conclude non-genetic factors impact the manifestation. Solid ideas are proposed in the discussion (such as altered mechanical forces - perhaps impacted by ECM production or muscle cell function). Evaluating those issues as well as the cell autonomy would go a long way to increase the impact of this study. As it stands, this work might be better suited for a more specialized journal.

**We appreciate the positive comments on our progress so far.**

**Like the Reviewer, we considered cell autonomy experiments, but decided that these were impractical given that a) the phenotype only arises in 30% of mutants, b) the cell/tissue-level phenotype only arises in late larvae and c) we predict that it will require a rather large area of mosaicism (i.e. a big patch of tissue of the transplanted genotype) to see either a defect in a wild type background or a rescue in a mutant background.**

**We have indeed shown there is a motility defect in both future scoliotic and non-scoliotic *yap1* mutant larvae. However, the key mechanistic step is to understand why some individuals develop scoliosis. In our view, showing a slightly earlier ECM defect in 30% of mutants would not really address that question – it would simply show that before there is frank scoliosis there is an ECM change, which is to be expected given that the bones are made of ECM. What is needed (and we do not have) is a molecular link between known Yap1 target genes and the scoliotic phenotype, so that rescue experiments could be performed. We believe, with the other Reviewers, that this is a step too much to ask at this stage.**

**However, we note that we have now added, in Fig. 6, important new data regarding an ECM defect. As there seems to be a fully penetrant early defect in hypochordal *col8a1a* mRNA accumulation in *yap1* mutants reared at the non-permissive temperature, it may be that even after rearing at the permissive temperature, some individuals with a local hypochord defect go on to develop focal scoliosis at later stages. Thus, a key future step will be to determine whether this defect causes the subsequent scoliosis, or whether Yap1 function elsewhere, or at another stage, is independently triggering scoliosis.**
