# Supplementary material for: Yap1 regulates motility and vertebral development and prevents kyphoscoliosis in zebrafish
Source: PLoS Genet. 2026 May 28;22(5):e1012172. doi: 10.1371/journal.pgen.1012172 (PMC13349305; doi:10.1371/journal.pgen.1012172)
Supplement: Peer review history

# Author comment: Author response to Decision Letter 0

**DOI:** 10.1371/journal.pgen.1012172.r001

**Transfer Alert**

This paper was transferred from another journal. As a result, its full editorial history (including decision letters, peer reviews and author responses) may not be present.

22 Jan 2026

# Peer review: Decision Letter 0

**DOI:** 10.1371/journal.pgen.1012172.r002

**Authors:** Pablo Wappner

26 Feb 2026

PGENETICS-D-26-00018

Yap1 regulates motility and vertebral development and prevents kyphoscoliosis in zebrafish

PLOS Genetics

Dear Dr. Hughes,

Thank you for submitting your manuscript to PLOS Genetics. After careful consideration, we feel that it has merit but does not fully meet PLOS Genetics's publication criteria as it currently stands. Therefore, we invite you to submit a revised version of the manuscript that addresses the points raised during the review process.

Please submit your revised manuscript within by Mar 28 2026 11:59PM. If you will need more time than this to complete your revisions, please reply to this message or contact the journal office at plosgenetics@plos.org. When you're ready to submit your revision, log on to https://www.editorialmanager.com/pgenetics/ and select the 'Submissions Needing Revision' folder to locate your manuscript file.

Please include the following items when submitting your revised manuscript:

* A letter that responds to each point raised by the editor and reviewer(s). You should upload this letter as a separate file labeled 'Response to Reviewers'. This file does not need to include responses to formatting updates and technical items listed in the 'Journal Requirements' section below.

* A marked-up copy of your manuscript that highlights changes made to the original version. You should upload this as a separate file labeled 'Revised Manuscript with Track Changes'.

* An unmarked version of your revised paper without tracked changes. You should upload this as a separate file labeled 'Manuscript'.

If you would like to make changes to your financial disclosure, competing interests statement, or data availability statement, please make these updates within the submission form at the time of resubmission. Guidelines for resubmitting your figure files are available below the reviewer comments at the end of this letter.

We look forward to receiving your revised manuscript.

Kind regards,

Cecilia Moens

Academic Editor

PLOS Genetics

Pablo Wappner

Section Editor

PLOS Genetics

Aimée Dudley

Editor-in-Chief

PLOS Genetics

Anne Goriely

Editor-in-Chief

PLOS Genetics

**Journal Requirements:**

1) Please provide an Author Summary. This should appear in your manuscript between the Abstract (if applicable) and the Introduction, and should be 150-200 words long. The aim should be to make your findings accessible to a wide audience that includes both scientists and non-scientists. Sample summaries can be found on our website under Submission Guidelines:

https://journals.plos.org/plosgenetics/s/submission-guidelines#loc-parts-of-a-submission

2) Please upload all main figures as separate Figure files in .tif or .eps format. For more information about how to convert and format your figure files please see our guidelines:

https://journals.plos.org/plosgenetics/s/figures

3) Some material included in your submission may be copyrighted. According to PLOSu2019s copyright policy, authors who use figures or other material (e.g., graphics, clipart, maps) from another author or copyright holder must demonstrate or obtain permission to publish this material under the Creative Commons Attribution 4.0 International (CC BY 4.0) License used by PLOS journals. Please closely review the details of PLOSu2019s copyright requirements here: PLOS Licenses and Copyright. If you need to request permissions from a copyright holder, you may use PLOS's Copyright Content Permission form.

Please respond directly to this email and provide any known details concerning your material's license terms and permissions required for reuse, even if you have not yet obtained copyright permissions or are unsure of your material's copyright compatibility. Once you have responded and addressed all other outstanding technical requirements, you may resubmit your manuscript within Editorial Manager.

Potential Copyright Issues:

- Please confirm (a) that you are the photographer of Figures 5A and S3A., or (b) provide written permission from the photographer to publish the photo(s) under our CC BY 4.0 license.

4) Please amend your detailed Financial Disclosure statement. This is published with the article. It must therefore be completed in full sentences and contain the exact wording you wish to be published.

- State the initials, alongside each funding source, of each author to receive each grant. For example: "This work was supported by the National Institutes of Health (####### to AM; ###### to CJ) and the National Science Foundation (###### to AM)."

- State what role the funders took in the study. If the funders had no role in your study, please state: "The funders had no role in study design, data collection and analysis, decision to publish, or preparation of the manuscript.".

If you did not receive any funding for this study, please simply state: u201cThe authors received no specific funding for this work.u201d

**Reviewers' comments:**

Reviewer's Responses to Questions

**Comments to the Authors:**

**Please note here if the review is uploaded as an attachment.**

Reviewer #1: This article investigates the role of Hippo signaling in zebrafish through mutations in yap1 and wwtr1. The authors show that yap1 and wwtr1 are expressed in somatic muscle, and that ablation of each gene individually produces only minor defects in larval trunk movements or mobility, with no obvious defects in muscle development or gross architecture. Functionally, wwtr1 mutants display normal larval swimming velocity, whereas yap mutants exhibit impaired swimming, indicating that yap contributes to functional swimming behaviors. Additionally, yap mutants raised at the restrictive temperature develop scoliosis in some individuals, despite an absence of obvious musculature defects. The onset of abnormal body curvature occurs at late larval stages and is accompanied by subtle morphologic changes in the vertebral bodies in adulthood. Finally, yap is required for normal larval growth (including overall body length) independent of scoliosis. While the study does not reveal a clear mechanism for scoliosis onset, its quantitative approach suggests that small differences in growth trajectories may destabilize tissues required to maintain a straight spine, with yap playing a contributing role. I would recommend acceptance of the manuscript after revisions, as suggested by other reviewers.

Concerns to address:

Introduction: Please re-think the statement that scoliosis reduces quality of life for over 3% of people. This is at face value simply not true. In truth only about 40K surgeries are performed in the US for scoliosis per year so the number are just not correct here. In fact, while some people due experience psychosocial distress at having a curvy spine and may require bracing or surgery, in truth most people with scoliosis have no noticeable effects associated with scoliosis. Moreover, I have met people with scoliosis that have challenged my own language about how this disorder is a ‘burden’, so please be mindful of your language.

Figure 2: Makes a claim that wwtr1 mutants are ‘defective in early larval movement’. However, swimming velocity is not affected at 5 or 11 dpf? Thus, this claim stands only on the evidence of a reduction in trunk angle measures in larval fish after electrical stimulation. I would question, if this change in trunk angle does not attribute to a functional measure of movement such as swimming velocity, if the conclusion is well supported?

Figure 3: Makes a claim that yap1 mutants are ‘defective in neuromuscular function’. Again I don’t think this conclusion is supported by the evidence. Instead, I only see changes in trunk angle measure, that are now supported by reduction in larval movement. This suggest that instead yap1 mutants are defective in larval movement, as stated for Figure 2. I think you would need to demonstrate that muscle or the neuromuscular junctions were effected to make the claim above.

Line 285-286: ‘yap1 and wwtr1 have significant motility defects’ As I argued for Figure 2 , I don’t think an issue with trunk angle measures can translate to significant motility defects for wwtr1 mutants, if they functional are able to swim at a velocity also attributed with wild type animals. How is this conclusion supported by evidence?

Lines 269 and 279: “staged in days, not dpf” Can you explain your metrics for staging? Standard length, myotome number, ect? It is very important to be concise about this so that others can learn what you did and perhaps do the same one day if they needed to.

Given that Yap1 has hydrocephalus in mice and that central canal cilia defects are associated with scoliosis in zebrafish. I am not sure that these cilia are not contributing to the larval onset of scoliosis, potentially destabilizing the Reissner fiber and causing scoliosis. IT is not the same to look at multiciliate cells in the brain as these cilia do not even form in zebrafish until much later than 13dpf (PMID: 26991819). Plus, the more phenotypic yap1 mutant embryos do display phenotypes associated with cilia defects, including edema and curved bodies. It may be interesting to look at the central canal motile cilia in these mutants.

Figure 4: The cartoon schematic is difficult to read. Please use white lettering or pull them out from the blue vertebral body background to make them easily seen. I would also suggest a bracket outside the vertebral body to represent the posterior height metric.

Reviewer #2: The manuscript by Williams-Ward et al Williams-Ward et. al unravels how YAP1 determines the motility and development of kyphoscoliosis in zebrafish. Scoliosis affects over 3% of people and is influenced by genetic, environmental, and mechanical factors. This study investigated the role of the mechanosensitive Hippo pathway genes yap1 and wwtr1 in zebrafish. The Authors found that loss of yap1 impaired growth, caused early motility defects, and led to vertebral abnormalities. Although initial defects were mild, many mutants later developed kyphoscoliosis, likely due to a positive mechanical feedback process. The findings suggest that Yap1 plays a key role in mechanosensory feedback between muscle, bone, and tendon to regulate vertebral growth and prevent spinal deformities.

The manuscript is interesting and addresses the role of the Yap1 and Wwtr1 in scoliosis. The Authors do provide some intriguing findings, although the mechanistic aspects are weak. Overall, the manuscript may be revised to address the specific comments below and reconsidered.

Major comments

1. The representative data for the wwtr1kg133/+ genotype should be included in Figure 2A. Are the gaps between muscle fibers increased in the wwtr1kg133 genotype at 3 and 5 dpf?

2. At the functional level, YAP and TAZ are effectors of the Hippo pathway, leading to binding to TEAD and target gene activation; are the effects shown here mediated by TEAD?

3. Have the Authors checked if Yap1 expression changes in the Wwtr1 mutant and vice versa?

4. The authors mention that yap1kg151 mutants show no possible defects in the notochord formation. However, are the vacuolated and epithelial cells able to secrete collagen and ECM comparable to wild types. Can authors comment on the expansion of these cells?

5. Is the shortening of the embryonic axis dependent on Dstyk (dual serine/threonine and tyrosine protein kinase) activity?

6. The authors have shown the expression profiles of myod and myog in the flat mounts (Fig S5B). Are the expression profiles of myosin heavy chain genes affected in the yap1kg151 mutants?

7. Previous studies have shown the importance of Reisnerr’s fibers (RF) in axial development (H Lu, 2020; Troutwine BR, 2020). Is the formation of RF normal in the mutants?

8. Is the sarcomere organisation defective in yap1kg151 mutants?

Minor comment:

I would highly recommend authors to change the arrangement of figures to sequential manner.

Reviewer #3: In their study, Williams-Ward and colleagues have used global deletion mutants of yap1 and wwtr1 to describe a kyphoscoliosis phenotype in adults (most pronounced in yap1 mutants). Compound genetic analysis confirm existing data that these factors have some degree of redundant function. While the manuscript is well written and the data are high quality, the study falls short of making a significant contribution to understanding the cell types that require yap1/wwtr1 for proper spine development. Given the less than full penetrance of the yap1 spine phenotype, the authors conclude non-genetic factors impact the manifestation. Solid ideas are proposed in the discussion (such as altered mechanical forces - perhaps impacted by ECM production or muscle cell function). Evaluating those issues as well as the cell autonomy would go a long way to increase the impact of this study. As it stands, this work might be better suited for a more specialized journal.

**********

**Have all data underlying the figures and results presented in the manuscript been provided?**

Large-scale datasets should be made available via a public repository as described in the *PLOS Genetics*  data availability policy, and numerical data that underlies graphs or summary statistics should be provided in spreadsheet form as supporting information.

Reviewer #1: Yes

Reviewer #2: Yes

Reviewer #3: Yes

**********

PLOS authors have the option to publish the peer review history of their article (what does this mean?). If published, this will include your full peer review and any attached files.

If you choose “no”, your identity will remain anonymous but your review may still be made public.

**Do you want your identity to be public for this peer review?**  For information about this choice, including consent withdrawal, please see our Privacy Policy.

Reviewer #1: No

Reviewer #2: **Yes:** Sam J Mathew

Reviewer #3: No

[NOTE: If reviewer comments were submitted as an attachment file, they will be attached to this email and accessible via the submission site. Please log into your account, locate the manuscript record, and check for the action link "View Attachments". If this link does not appear, there are no attachment files.]

**Figure resubmission:**

-->While revising your submission, we strongly recommend that you use PLOS’s NAAS tool (https://ngplosjournals.pagemajik.ai/artanalysis) to test your figure files. NAAS can convert your figure files to the TIFF file type and meet basic requirements (such as print size, resolution), or provide you with a report on issues that do not meet our requirements and that NAAS cannot fix.

After uploading your figures to PLOS’s NAAS tool - https://ngplosjournals.pagemajik.ai/artanalysis, NAAS will process the files provided and display the results in the "Uploaded Files" section of the page as the processing is complete. If the uploaded figures meet our requirements (or NAAS is able to fix the files to meet our requirements), the figure will be marked as "fixed" above. If NAAS is unable to fix the files, a red "failed" label will appear above. When NAAS has confirmed that the figure files meet our requirements, please download the file via the download option, and include these NAAS processed figure files when submitting your revised manuscript.

**Reproducibility:**

To enhance the reproducibility of your results, we recommend that authors deposit laboratory protocols in protocols.io, where a protocol can be assigned its own identifier (DOI) such that it can be cited independently in the future. Additionally, PLOS ONE offers an option to publish peer-reviewed clinical study protocols. Read more information on sharing protocols at https://plos.org/protocols?utm_medium=editorial-email&utm_source=authorletters&utm_campaign=protocols

# Author comment: Author response to Decision Letter 1

**DOI:** 10.1371/journal.pgen.1012172.r003

20 Apr 2026

Attachment Submitted filename: ResponsetoReviewers260417.docx

# Editor report: Decision Letter 1

**DOI:** 10.1371/journal.pgen.1012172.r004

**Authors:** Pablo Wappner

18 May 2026

Dear Simon,

We are pleased to inform you that your manuscript entitled "Yap1 regulates motility and vertebral development and prevents kyphoscoliosis in zebrafish" has been editorially accepted for publication in PLOS Genetics. Congratulations! Thank you for carefully considering the reviewer's comments and for adding interesting further data to the manuscript.

Before your submission can be formally accepted and sent to production you will need to complete our formatting changes, which you will receive in a follow up email. Please be aware that it may take several days for you to receive this email; during this time no action is required by you. Please note: the accept date on your published article will reflect the date of this provisional acceptance, but your manuscript will not be scheduled for publication until the required changes have been made.

Once your paper is formally accepted, an uncorrected proof of your manuscript will be published online ahead of the final version, unless you’ve already opted out via the online submission form. If, for any reason, you do not want an earlier version of your manuscript published online or are unsure if you have already indicated as such, please let the journal staff know immediately at plosgenetics@plos.org.

In the meantime, please log into Editorial Manager at https://www.editorialmanager.com/pgenetics/, click the "Update My Information" link at the top of the page, and update your user information to ensure an efficient production and billing process. Note that PLOS requires an ORCID iD for all corresponding authors. Therefore, please ensure that you have an ORCID iD and that it is validated in Editorial Manager. To do this, go to ‘Update my Information’ (in the upper left-hand corner of the main menu), and click on the Fetch/Validate link next to the ORCID field.  This will take you to the ORCID site and allow you to create a new iD or authenticate a pre-existing iD in Editorial Manager.

If you have a press-related query, or would like to know about making your underlying data available (as you will be aware, this is required for publication), please see the end of this email. If your institution or institutions have a press office, please notify them about your upcoming article at this point, to enable them to help maximise its impact. Inform journal staff as soon as possible if you are preparing a press release for your article and need a publication date.

Thank you again for supporting open-access publishing; we are looking forward to publishing your work in PLOS Genetics!

Yours sincerely,

Cecilia Moens

Academic Editor

PLOS Genetics

Pablo Wappner

Section Editor

PLOS Genetics

Aimée Dudley

Editor-in-Chief

PLOS Genetics

Anne Goriely

Editor-in-Chief

PLOS Genetics

www.plosgenetics.org

BlueSky: @plos.bsky.social

----------------------------------------------------

Comments from the reviewers (if applicable):

----------------------------------------------------

**Data Deposition**

If you have submitted a Research Article or Front Matter that has associated data that are not suitable for deposition in a subject-specific public repository (such as GenBank or ArrayExpress), one way to make that data available is to deposit it in the Dryad Digital Repository. As you may recall, we ask all authors to agree to make data available; this is one way to achieve that. A full list of recommended repositories can be found on our website.

The following link will take you to the Dryad record for your article, so you won't have to re‐enter its bibliographic information, and can upload your files directly:

http://datadryad.org/submit?journalID=pgenetics&manu=PGENETICS-D-26-00018R1

More information about depositing data in Dryad is available at http://www.datadryad.org/depositing. If you experience any difficulties in submitting your data, please contact help@datadryad.org for support.

Additionally, please be aware that our data availability policy requires that all numerical data underlying display items are included with the submission, and you will need to provide this before we can formally accept your manuscript, if not already present.

----------------------------------------------------

**Press Queries**

If you or your institution will be preparing press materials for this manuscript, or if you need to know your paper's publication date for media purposes, please inform the journal staff as soon as possible so that your submission can be scheduled accordingly. Your manuscript will remain under a strict press embargo until the publication date and time. This means an early version of your manuscript will not be published ahead of your final version. PLOS Genetics may also choose to issue a press release for your article. If there's anything the journal should know or you'd like more information, please get in touch via plosgenetics@plos.org.

# Editor report: Acceptance letter

**DOI:** 10.1371/journal.pgen.1012172.r005

**Authors:** Pablo Wappner

PGENETICS-D-26-00018R1

Yap1 regulates motility and vertebral development and prevents kyphoscoliosis in zebrafish

Dear Dr Hughes,

We are pleased to inform you that your manuscript entitled "Yap1 regulates motility and vertebral development and prevents kyphoscoliosis in zebrafish" has been formally accepted for publication in PLOS Genetics! Your manuscript is now with our production department and you will be notified of the publication date in due course.

The corresponding author will soon be receiving a typeset proof for review, to ensure errors have not been introduced during production. Please review the PDF proof of your manuscript carefully, as this is the last chance to correct any errors. Please note that major changes, or those which affect the scientific understanding of the work, will likely cause delays to the publication date of your manuscript.

Soon after your final files are uploaded, unless you have opted out or your manuscript is a front-matter piece, the early version of your manuscript will be published online. The date of the early version will be your article's publication date. The final article will be published to the same URL, and all versions of the paper will be accessible to readers.

For Research Articles, you will receive an invoice from PLOS for your publication fee after your manuscript has reached the completed accept phase. If you receive an email requesting payment before acceptance or for any other service, this may be a phishing scheme. Learn how to identify phishing emails and protect your accounts at https://explore.plos.org/phishing.

Thank you again for supporting PLOS Genetics and open-access publishing. We are looking forward to publishing your work!

With kind regards,

Anita Estes

PLOS Genetics

On behalf of:

The PLOS Genetics Team

Carlyle House, Carlyle Road, Cambridge CB4 3DN | United Kingdom

plosgenetics@plos.org | +44 (0) 1223-442823

plosgenetics.org | Twitter: @PLOSGenetics
